# Protein stabilization by tuning the steric restraint at the reverse turn†
†Electronic supplementary information (ESI) available. See DOI: 10.1039/c7sc05163h


**DOI:** 10.1039/c7sc05163h

**Published:** 2018-04-24

**Authors:** Priyanka Lahiri, Hitesh Verma, Ashraya Ravikumar, Jayanta Chatterjee

**Affiliations:** a Molecular Biophysics Unit , Indian Institute of Science , Bangalore 560012 , India . Email: jayanta@iisc.ac.in

## Abstract

Reverse turns are solvent-exposed motifs in proteins that are crucial in nucleating β-sheets and drive the protein folding. The solvent-exposed nature makes reverse turns more amenable to chemical modifications than α-helices or β-sheets towards modulating the stability of re-engineered proteins. Here, we utilize van der Waals repulsive forces in tuning the steric restraint at the reverse turn. The steric restraint induced upon *N*-methylation of the *i*+1–*i*+2 amide bond at the reverse turn results in well-folded and stable β-sheets in aqueous solution at room temperature. The developed superactive turn inducing motif is tolerant to a wide variety of functional groups present on coded amino acids making the designed turn fully compatible with bioactive loops in proteins. We demonstrate that the steric restraint and the functional groups at the reverse turn act in synergy to modulate the folding of re-engineered β-sheets. Introduction of the turn motifs onto a three-stranded β-sheet protein, Pin 1 WW domain, resulted in various analogs showing a cooperative two-state transition with thermal stability (*T*_M_) ranging from 62 °C to 82 °C. Despite modulating the stability of Pin 1 variants by ∼2.8 kcal mol^–1^ (ΔΔ*G*_f_), the native fold in all the protein variants was found to be unperturbed. This structural stability is brought about by conformational preorganization at the engineered reverse turn that results in strong intramolecular hydrogen bonds along the three dimensional structure of the protein. Thus, this simple loop engineering strategy *via* two amino acid substitution provides us a “toolkit” to modulate the stability of β-sheet containing peptides and proteins in aqueous solution that will greatly expand the scope of *de novo* protein and foldamer design.

## Abstract 2

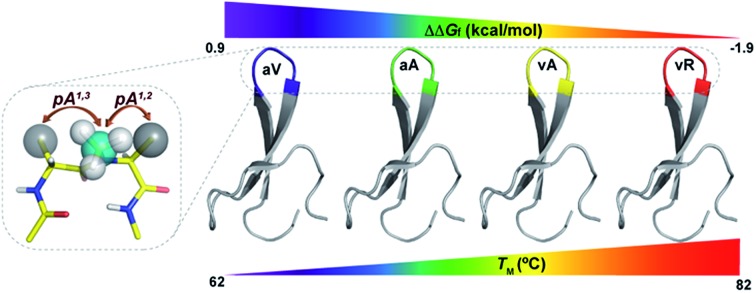
The incorporation of pseudoallylic strain by *N*-methylation at the solvent exposed loop in proteins leads to a stark increase in their thermodynamic stability that can be tuned by altering the amino acid composition.

## Introduction

An elegant combination of various secondary structure motifs, *i.e.*, α-helices, β-strands and reverse turns acts as a framework for any protein structure.^1^ Recapitulation of such secondary structure elements in isolation from the protein environment is a challenging task and often requires covalent/non-covalent constraints to recover their native conformational and functional properties.^2^ Nevertheless, this approach has found tremendous utility in delivering small to medium sized constrained peptides and proteins and has revolutionized the development of peptide based therapeutics2*c* and the generation of novel non-antibody based scaffolds.^3^

Utilizing such covalent and non-covalent constraints to engineer proteins and enzymes with improved thermodynamic stability has been another desirable area of interest. Tight packing in the hydrophobic core of a protein by optimizing the van der Waals interaction *via* substitution with non-polar amino acids is one of the most common strategies to enhance the protein stability.^4^ However, it was observed that if the volume of amino acid substitution is above the tolerance limit, the local increase in strain energy leads to de-stabilization of re-engineered proteins.^5^ Likewise, covalent tethering of protein secondary structures by disulfide bridge formation between canonical and noncanonical amino acid pairs and recently by thioether stapling has also found great success in enhancing the protein and enzyme stability.^6^

Free peptide bond rotation is a major driving force for the unfolding of a protein.^7^ Thus, the restriction of such free bond rotation at a site that is important for the folding of a protein would have a significant contribution towards reducing the conformational entropy of the unfolded state. β-Turns are such regions in a protein which are known to drive the protein folding process by nucleating the formation of β-sheets.^8^ Moreover, unlike α-helices and β-sheets the distinguishing feature of β-turns is their occurrence at solvent-exposed sites in globular proteins. Thus, in the context of protein engineering, β-turns are more amenable to chemical modifications, which would minimally perturb the native structure of a protein. A few turn-mimetics like *R*-nipecotic acid-*S*-nipecotic acid,^9^ charged dibenzofuran,^10^ Hot = Tap (derived from fusion of γ-hydroxythreonine and 4-thiaproline)^11^ and tetrasubstituted alkenes^12^ have been successfully utilized to re-engineer the conformational stability of (semi)synthetic proteins. However, these turn mimetics require elaborate chemical synthesis and lack the functional group diversity found in reverse turns of bioactive proteins. Therefore, we are particularly keen to develop super-active turn-inducing motifs8*e* bearing a diverse set of amino acids with different functional groups that can be easily and site-selectively incorporated into proteins *via* synthetic and semi-synthetic methods.

**Fig. 1:**
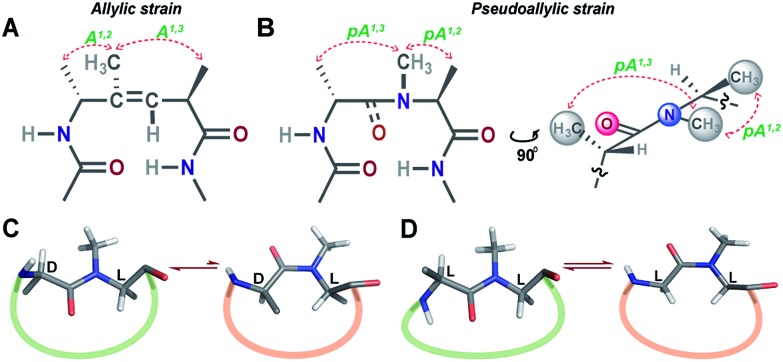
(A) The trisubstituted alkene isostere with the *A*^*1,2*^ and *A*^*1,3*^ allylic strain; note the hydrophobic nature of the reverse turn. (B) *N*-Methylated *i*+2 residue at the reverse turn displaying the pseudoallylic strain *pA*^*1,3*^ and *pA*^*1,2*^ between heterochiral residues. A side view representation of pseudoallylic strain *pA*^*1,3*^ and *pA*^*1,2*^. (C) In cyclic peptides with *N*-methylated heterochiral residues, the *trans* conformation of the *N*-methylated amide bond is energetically more favorable than the *cis* conformation. (D) While, in cyclic peptides with *N*-methylated homochiral residues, both *cis* and *trans* conformations are energetically favorable, as observed from the solution NMR studies (ESI†).

Unlike most earlier reported studies that utilize a covalent restraint to minimize the conformational entropy at the reverse turn, we chose to adopt a strategy based on the non-covalent van der Waals repulsion to tune the steric interactions at the β-turn. Inspired by our earlier study, we minimally modified the *i*+1–*i*+2 amide bond at the turn by *N*-methylation.^13^ Surprisingly, a single *N*-methylation was found to introduce a favorable pseudoallylic strain between various atoms of the *i*+1 and *i*+2 residues in the β-turn (Fig. 1), thereby minimizing its conformational entropy and imparting thermodynamic stability to the re-engineered proteins. We observed that the pseudoallylic *pA*^*1,3*^ strain is critical in nucleating the formation of a β-sheet in aqueous solution than the pseudoallylic *pA*^*1,2*^ strain. However, the presence of both *pA*^*1,3*^ and *pA*^*1,2*^ strain enhances the foldedness of the peptide. Since this pseudoallylic strain is common to any α-amino acid bearing a C^β^, it allowed us to functionalize the reverse turn with any desired amino acid. Furthermore, our screening led to the identification of several super-active turn inducing motifs that show remarkable thermodynamic stability of the re-engineered three-stranded β-sheet protein, Pin 1 WW domain, without altering its native conformation.

## Results and discussion

### Differential control of *cis*/*trans* isomerism in peptide bonds by pseudoallylic strain

Towards re-engineering the conformational stability of proteins while maintaining the polar functional group diversity observed at the reverse turn, we sought to minimally modify the *i*+1–*i*+2 amide bond. We were inspired by the turn-inducing properties of trisubstituted alkene isosteres, in which the allylic *A*^*1,3*^ and *A*^*1,2*^ strain (Fig. 1A) plays a critical role in folding the polypeptide into a β-sheet conformation.^12^,^14^ However, the loss of electrostatic potential associated with the substitution of the amide bond with *E*-alkene that is devoid of any amino acid functional groups at the *i*+1 and *i*+2 sites^15^ could severely compromise the bioactivity of the re-engineered protein, wherein the molecular recognition is mediated by the amino acid sequence at the reverse turn.

*N*-Methylation of an amide bond induces conformational restriction in a peptide backbone due to the pseudoallylic strain *pA*^*1,3*^ and *pA*^*1,2*^ between _(*i*)_C^NMe^/_(*i*–1)_C^β^ and _(*i*)_C^NMe^/_(*i*)_C^β^, respectively (Fig. 1B).^16^ By analyzing the conformations of various literature reported *N*-methylated model and bioactive cyclic peptides (ESI†), we identified that the *N*-methylation-induced pseudoallylic strain between two heterochiral residues (l-/d- or d-/l-) strictly favors a *trans* peptide conformation, whereas when present between two homochiral residues (l-/l- or d-/d-), there is an equal probability for the *N*-methylated peptide bond to adopt a *cis* or a *trans* conformation (Fig. 1C and D).^17^

This finding encouraged us to evaluate the role of pseudoallylic strain in governing the *cis*/*trans* equilibrium of the *N*-methylated peptide bond in unrestrained linear peptides (Table 1) using NMR in aqueous buffer. We observed that in addition to the *pA*^*1,2*^ strain (**L1**), the introduction of *pA*^*1,3*^ strain increases the *K*_*trans*/*cis*_ in the heterochiral system (**L3**) and significantly decreases the *K*_*trans*/*cis*_ in the homochiral system (**L2**). The reduced *K*_*trans*/*cis*_ in the homochiral system is a result of the unfavorable 1,3-diaxial strain in the *trans* conformation, which is absent in the heterochiral system (ESI†). Furthermore, the *pA*^*1,3*^ strain (**L4**) alone does not provide sufficient conformational restriction to favor the *trans* conformation; however, in combination with the *pA*^*1,2*^ strain (**L3**) it increases the *K*_*trans*/*cis*_. These results clearly indicate that the differential behavior of an *N*-methylated peptide bond depends on the neighboring substituents with the highest propensity to adopt a *trans* conformation when the flanking residues are heterochiral.

**Table 1:** *K*_*trans*/*cis*_ of linear tetrapeptides determined by ^1^H NMR in acetate buffer (pH 3.8) at 25 °C. The lower case indicates d-amino acid

Peptide	Strain	Sequence	*K*_*trans*/*cis*_
**L1**	*pA* ^*1,2*^	Ac-V**G**(*NMe*)**A**F-NH_2_	4.7
**L2**	*pA* ^*1,3*^, *pA*^*1,2*^	Ac-V**A**(*NMe*)**A**F-NH_2_	2.2
**L3**	*pA* ^*1,3*^, *pA*^*1,2*^	Ac-V**a**(*NMe*)**A**F-NH_2_	5.9
**L4**	*pA* ^*1,3*^	Ac-V**a**(*NMe*)**G**F-NH_2_	1.8

### Evidence of conformational restriction imposed by *N*-methylation

**Fig. 2:**
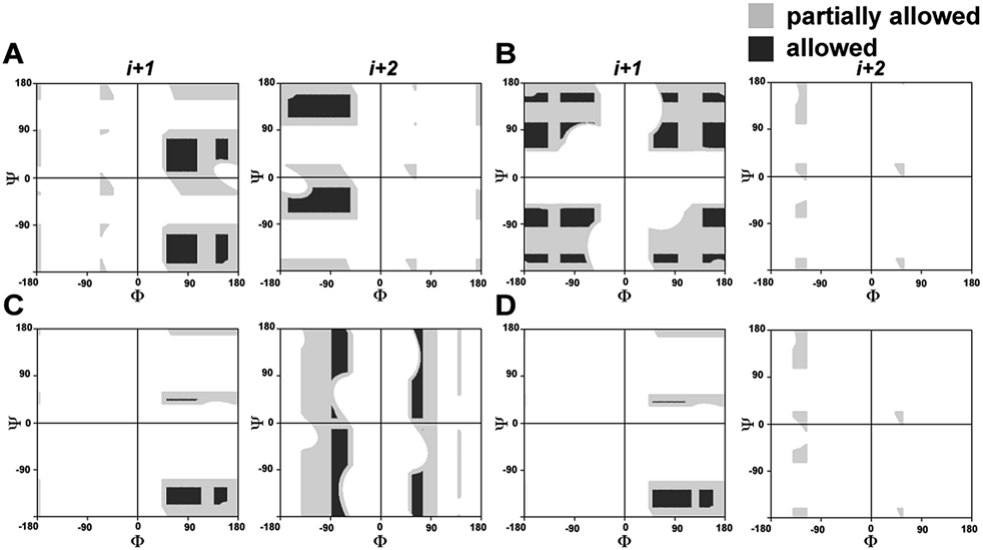
Partially allowed (grey) and allowed (black) torsion angles of the flanking amino acid residues (*i*+1 and *i*+2) about non-*N*-methylated and singly *N*-methylated peptide bonds. (A) Ac-AaAA-NH_2_ (absence of *pA*^*1,3*^ and *pA*^*1,2*^ strain); (B) Ac-AG(*NMe*)AA-NH_2_ (only *pA*^*1,2*^ strain); (C) Ac-Aa(*NMe*)GA-NH_2_ (only *pA*^*1,3*^ strain); (D) Ac-Aa(*NMe*)AA-NH_2_ (both *pA*^*1,3*^ and *pA*^*1,2*^ strain).

In order to understand the conformational restraint imposed by *N*-methylation of the peptide bond, we analyzed the Ramachandran plot of *N*-methylated linear tetrapeptides with varying pseudoallylic strain. We observed that irrespective of the chirality of *N*-methylated residue, *N*-methylation restrains *Ψ* of the preceding residue (compare *Ψ*_*i*+1_ in Fig. 2A with that in Fig. 2C and D). On the other hand, *N*-methylation constrains both *Φ* and *Ψ* of the *N*-methylated residue (Fig. 2B–D). We clearly noted that the introduction of *pA*^*1,3*^ strain (*e.g.*, *i*+1 residue in Fig. 2D as opposed to *i*+1 residue in Fig. 2B) or *pA*^*1,2*^ strain (*e.g.*, *i*+2 residue in Fig. 2D as opposed to *i*+2 residue in Fig. 2C) significantly restricts the allowed conformational space about the *N*-methylated peptide bond. This finding correlates well with the restricted conformational freedom of *N*-methylated residues obtained from conformational energy calculations in *N*-methylated alanine and phenylalanine dipeptide models by Momany.^18^

### Incorporation of pseudoallylic strain yields stable β-hairpins in aqueous solution

To demonstrate the applicability of pseudoallylic strain in inducing stable β-turns, we engineered the *i*+1 and *i*+2 residues in the reverse turn of a water-soluble β-sheet peptide, **pG** from the Gellman laboratory (Table 2).^19^

**Table 2:** Peptide sequences with identical strand residues but varying reverse turn motifs displaying differing pseudoallylic strain

Peptide	Strain	Sequence
**1**	*pA* ^*1,2*^	Ac-RYVEV**G**(*NMe*)**A**KKILQ-NH_2_
**2**	*pA* ^*1,3*^	Ac-RYVEV**a**(*NMe*)**G**KKILQ-NH_2_
**3**	*pA* ^*1,3*^, *pA*^*1,2*^	Ac-RYVEV**a**(*NMe*)**A**KKILQ-NH_2_
**4**	—	Ac-RYVEV**aA**KKILQ-NH_2_
**pG**	—	Ac-RYVEV**pG**KKILQ-NH_2_

**Fig. 3:**
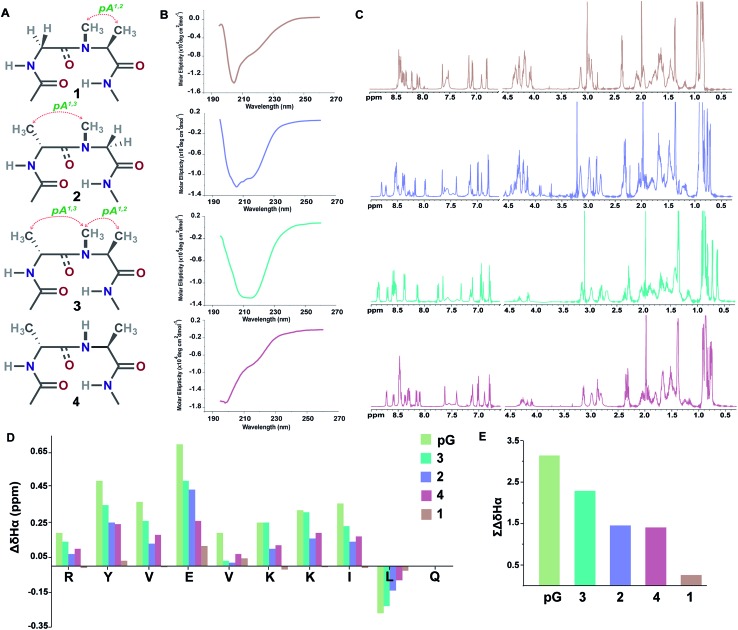
(A) Interplay of pseudoallylic strain (*pA*^*1,2*^ and *pA*^*1,3*^) in the reverse turn to induce the β-sheet folding. Note the spatial positioning of the methyl groups between *i*+1 C^α^ and *i*+2 C^α^ which modulates the folding of the polypeptide in aqueous solution at 25 °C. (B) CD spectrum of the peptides in acetate buffer (pH 3.8). (C) ^1^H NMR in acetate buffer (pH 3.8), clearly indicating the enhanced dispersion of the H^N^ resonance and upfield shifted, distinguishable Leu11 H^δ1/2^ in the well-folded peptides. (D) Secondary chemical shifts obtained from the respective random coil control peptides, where the d-residue is substituted with the l-residue. Leu11 H^α^ shows a negative deviation due to its proximity to the phenyl ring of Tyr2 in the folded analogs. In **1**, the secondary chemical shifts were determined by subtracting the H^α^ chemical shifts from the mean random coil chemical shifts of the respective residues obtained from the random coils of **2** and **3**. The ring constrained reverse turn motif, **pG**, was taken as a reference.^19^ (E) Summation of the secondary chemical shifts; only the absolute values were considered.

Initially, we synthesized four molecules with varying pseudoallylic strain in the reverse turn to analyze the critical role of *N*-methylation in inducing the β-sheet stability (Table 2). We acquired the CD (circular dichroism) spectrum at room temperature (25 °C) and pH 3.8 to obtain qualitative information on the foldedness of each peptide. Comparing the CD spectrum of **1**, **4** and **2**, **3** (Fig. 3B), we observed that only the latter showed the signature of a β-sheet peptide with a broad minimum centered at about 215 nm. Next, to quantitate the folding of each peptide, we resorted to 2D NMR studies at 25 °C (pH 3.8). The ^1^H NMR clearly indicated enhanced dispersion of HN resonance and upfield shift of Leu11 H^δ1/2^ in the order **3** > **2** > **4** > **1** (Fig. 3C) which is not a consequence of the peptide aggregation as determined by the ^1^H NMR dilution experiments (ESI†). This order corroborates well with the H^α^ secondary chemical shifts of the strand residues in the individual peptides, as residues in a β-strand undergo a downfield shift as compared to a random coil (Fig. 3D).^20^ We also determined the summation of H^α^ secondary chemical shifts, which indicates the overall foldedness of the β-sheet peptide (Fig. 3E).^21^ The best folding is observed in **3** with both *pA*^*1,2*^ and *pA*^*1,3*^ followed by **2** with only *pA*^*1,3*^. Thus, *pA*^*1,3*^ is crucial in nucleating a stable reverse turn which is further strengthened by *pA*^*1,2*^, verifying our theoretical findings from the Ramachandran plot. Although, the control peptide **4** indicates that the *i*+1 and *i*+2 C^β^ induces a certain amount of conformational restriction in the reverse turn, the introduction of an additional CH_3_ moiety in the amide bond (**3**), due to van der Waals repulsion, provides added stability to the β-sheet.

Encouraged by this finding, we set out to systematically scan the *i*+1 and *i*+2 residues of the reverse turn in the β-sheet peptide with diverse functional groups, but fixed *N*-methylation at the *i*+1–*i*+2 amide bond.

### Functional group diversity in the reverse turn of β-hairpin peptides

**Fig. 4:**
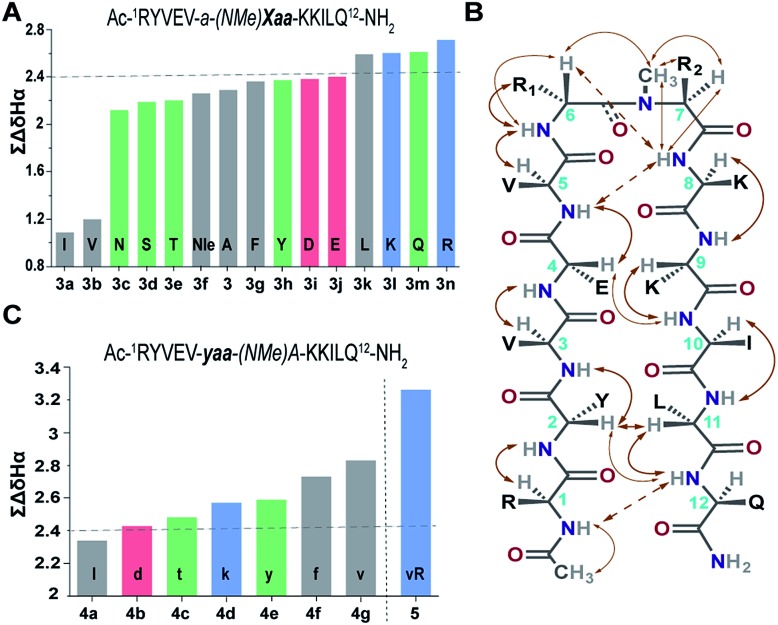
(A) ∑CSD of the primary library with l-amino acid substitutions (**Xaa**) at the *i*+2 site. (B) The characteristic NOEs, where the thick arrows denote strong (1.8–2.1 Å), the thin arrows denote medium (2.2–2.5 Å) and the broken arrows denote weak (>2.6 Å) NOEs. (C) ∑CSD of the secondary library with *i*+1 substitutions (**yaa**) along with **5** derived from the combination of **4g** and **3n**. All the NMR measurements were done in acetate buffer (pH 3.8) at 25 °C.

Our primary library was based on **3** that showed the maximum foldedness. Since the pseudoallylic strain *pA*^*1,3*^ is critical in folding the peptide, keeping the *i*+1 substitution constant, we initially sought to substitute the *i*+2 residue with various l-amino acid side chains and assess their influence on the β-sheet stability using ∑CSD (Fig. 4A).^21^ The characteristic NOEs observed in these compounds (Fig. 4B) indicated the occurrence of a βII′ turn, where the *i*+2 *N*-methyl and the *i*+2 C^β^ are syn-periplanar. Most amino acids were tolerated at the *i*+2 site; however, the unfavorable steric clash (*pA*^*1,2*^ strain) between *i*+2 *N*-methyl and *i*+2 C^γ^ (due to the unrestricted rotation about *χ*_1_ in valine and isoleucine) leads to destabilization of **3b** and **3a**. In contrast, in **3e** the β-branch demonstrates almost a two-fold increase in the ∑CSD resulting from the restricted *χ*_1_ rotation due to a probable intramolecular hydrogen bond between Thr–OH and its C=O (ESI†). Moreover, when the distance between the isopropyl group and the *N*-methyl group increases, *e.g.*, in the γ-branched amino acid leucine (**3k**), we obtained a more than two-fold increase in the ∑CSD compared to that of **3b**. Another notable observation was the increase in the foldedness of the β-sheet by incorporating positive charges, *e.g.*, in **3l** and **3n** at the termini of an unbranched amino acid side chain **3f**. These results show how modulation of local steric interactions and charges at a single site in the β-turn can alter the overall foldedness of the β-sheet.

Next, towards the thermodynamic analysis of *i*+2 substituted linear peptides, we estimated the folded population of selected β-sheets and their free energy of folding (Δ*G*_fold_) in water at 25 °C (Table 3) utilizing the respective fully folded disulfide bridged cyclic variant and unfolded random coil peptides by following the established methods.^19^,^22^ Overall, we observed that the increase in ∑CSD correlates well with the folded population of the β-sheets. We note that the introduction of *pA*^*1,3*^ alone is not sufficient to form an isolated stable β-sheet in water as reflected by the Δ*G*_f_ of 0.17 ± 0.2 kcal mol^–1^ in **2**. However, with the additional incorporation of *pA*^*1,2*^, the equilibrium shifts towards the folded side as observed in **3** (Δ*G*_f_ of –0.48 ± 0.1 kcal mol^–1^). The presence of a hydrophobic β-branch (**3a** and **3b**) is detrimental to the folding of the peptide, whereas a –CH_3_ to –OH substitution dramatically alters the folded population (ΔΔ*G*_f_ ∼ –0.6 kcal mol^–1^ between **3b** and **3e**). Thus, we observe that a combination of *pA*^*1,2*^ and polar functional groups enhances the stability of the β-sheet by –1.0 kcal mol^–1^ (ΔΔ*G*_f_ between **3n** and **2**).

**Table 3:** Folded population of the reporter residues and their thermodynamic analysis deduced from NMR in acetate buffer (pH 3.8) at 25 °C

	Fraction folded^*a*^ (%)				% folded population	Δ*G*_fold_ (kcal mol^–1^)
	Val3	Val5	Lys8	Ile10		
**2**	39	60	36	38	43 ± 10	0.17 ± 0.2
**3**	68	80	68	59	69 ± 7	–0.48 ± 0.1
**3b**	38	63	32	30	41 ± 13	0.22 ± 0.2
**3e**	64	80	67	59	67 ± 8	–0.42 ± 0.2
**3n**	78	99	76	70	81 ± 11	–0.86 ± 0.3
**4g**	84	99	81	75	85 ± 9	–1.03 ± 0.3
**5**	95	99	89	85	92 ± 5	–1.44 ± 0.4
**pG**	81	87	83	76	82 ± 3	–0.90 ± 0.1

^*a*^Fraction folded and Δ*G*_fold_ were calculated from the respective fully folded disulfide bridged cyclic variant and an unfolded random coil by established methods (ESI).

Subsequently, we constructed a secondary library with selected d-amino acid substitutions at the *i*+1 site with *N*-methylated alanine at the *i*+2 site. To our satisfaction, we observed the strong tolerance of various functional groups at the reverse turn (Fig. 4C). Of note, the β-branched amino acid valine, when present at the *i*+2 site (**3b**), by virtue of the unfavorable *pA*^*1,2*^ strain destabilizes the β-sheet, whereas when present at the *i*+1 site, the favorable *pA*^*1,3*^ strain enhances the stability of the β-sheet (**4g**) which is comparable to the stability of the ring constrained reverse turn motif **pG** (Table 3). Thus, the presence of an isopropyl group results in a 0.5–0.7 kcal mol^–1^ destabilization (ΔΔ*G*_f_ between **3** and **3b**) or stabilization (ΔΔ*G*_f_ between **3** and **4g**) when present at the *i*+2 or *i*+1 site, respectively (Table 3).

### *i*+1 and *i*+2 residues induce a concerted effect on β-sheet stability

The enhanced thermodynamic stability of the β-sheet resulting from the conformational restriction in the reverse turn by the isopropyl group at the *i*+1 site led us to re-engineer the most folded analog obtained from the *i*+2 substitution library (**3n**). To our amazement, the introduction of the *i*+1 isopropyl group into **3n** resulted in an additional 0.58 kcal mol^–1^ stabilization (ΔΔ*G*_f_ between **5** and **3n**) yielding a super stable monomeric β-sheet **5**. This result suggests that the *i*+1 and *i*+2 substituents act synergistically to enhance the foldedness of the engineered β-sheet. Additionally, although almost all functional groups are tolerated at the reverse turn, certain amino acids, leucine, lysine, glutamine, and arginine at the *i*+2 site and d-aspartate, d-threonine, d-lysine, d-tyrosine, d-phenylalanine and d-valine at the *i*+1 site, display high propensity to stabilize the β-sheet (demarcated by the dotted line at ∑CSD of 2.4 in Fig. 4A and C).

**Fig. 5:**
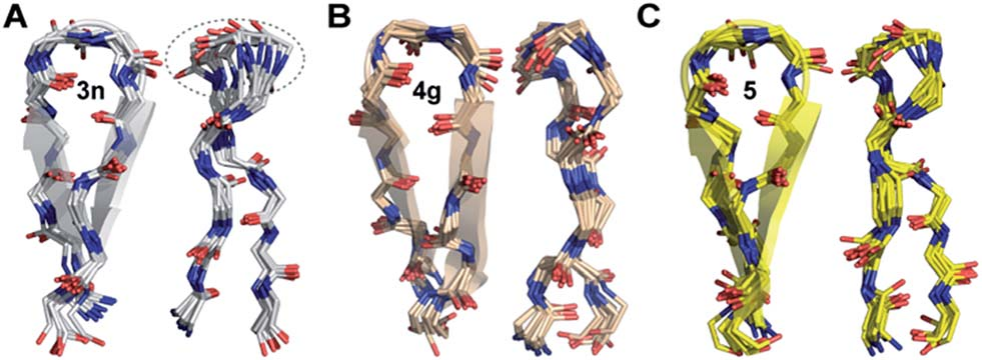
The backbone overlay of an ensemble of ten minimum energy conformations obtained from 10 ns restrained molecular dynamics simulation of (A) **3n**, (B) **4g** and (C) **5** in water. Note the flexibility of the β-turn with a shorter β-strand registry in **3n**. The 90° rotation of the β-sheets clearly depicts the enhanced right-handed twist in **3n** and **5**. The dotted lines indicate the flexibility of the turn in **3n** as opposed to **5** and **4g**. The side chains are omitted for the sake of clarity (ESI†). The NMR spectra of **3n**, **4g** and **5** were acquired in acetate buffer (pH 3.8) at 25 °C.

To gain molecular-level insight into the factors responsible for the enhanced thermodynamic stability of the β-sheets, we determined the high-resolution NMR structures of **3n**, **4g** and **5**, which show an increasing order of stability. The conformation of the three β-sheets revealed that the presence of β-branched d-valine at the *i*+1 residue in **4g** (Fig. 5B) and **5** (Fig. 5C) leads to conformational restriction at the β-turn as opposed to non-β-branched residues as observed in **3n** (Fig. 5A). This conformational restriction was also demonstrated by the narrow conformational space of the β-branched *i*+1 residue as opposed to the non-β-branched *i*+2 residue of **4g** and **5** in the Ramachandran plots of the conformational ensemble (ESI†). We also noted that the *Φ* and *Ψ* values of the turn residues in **3n**, **4g**, and **5** (Table 4) represent a βII′ type turn and beautifully overlap with the theoretically predicted values of these torsion angles by the introduction of *pA*^*1,2*^ and *pA*^*1,3*^ strain in linear tetrapeptides (Fig. 2D).

**Table 4:** Torsion angles at the reverse turn of the NMR derived structures

	*i*+1		*i*+2	
	*Φ*	*Ψ*	*Φ*	*Ψ*
**3n**	59 ± 12	–115 ± 11	–115 ± 10	8 ± 10
**4g**	62 ± 10	–123 ± 9	–101 ± 13	–1 ± 11
**5**	70 ± 9	–131 ± 9	–111 ± 11	13 ± 11
**βII′**	60	–120	–80	0

Thus, this conformational restriction results in a well-formed β-turn with a strong hydrogen bond between Lys8H^N^ and Val5CO, which plays a critical role in the folding transition of a β-hairpin^23^ and eventually dictates the elongated registry of the β-strands in **4g** and **5**. The temperature coefficient of all the amide protons except for Lys8H^N^ in these β-hairpins shows very high temperature dependence indicating the β-turn to be the most persistent structure in the hairpin.^24^ Curiously, the extent of foldedness in these hairpin peptides measured in terms of Δ*G*_fold_ (Table 3) corroborates with the temperature coefficient^25^ of Lys8H^N^ (Δ*δ*/Δ*T* of –3.6 ppb K^–1^ (**3b**) < –2.3 ppb K^–1^ (**2**) < –1.6 ppb K^–1^ (**3**) < –1.0 ppb K^–1^ (**3n**) ≅ –1.0 ppb K^–1^ (**4g**) < –0.6 ppb K^–1^ (**5**)). Thus, it is tempting to speculate that the increased conformational restriction in the β-turn possibly results in an enhanced strength of the intramolecular hydrogen bond stabilizing the β-turn.

Another remarkable feature was the stark right-handed twist observed in **3n** and **5** typical of β-sheet rich proteins^26^ owing to the presence of arginine at the *i*+2 site in the reverse turn. The observed right-handed twist was also indicated by the presence of strong NOEs between Lys9H^β/γ^ and Tyr2H^δ^ in the ROESY spectrum^27^ and a slight red-shift of the CD minimum in **3n** and **5** as opposed to **4g** (ESI†).^28^ Although we failed to fully understand the mechanistic origin of this right-handed twist, the conformation of these two peptides determined *in vacuo* indicates that the guanidinium group in arginine might be involved in hydrogen bonding with the peptide backbone, playing a critical role in governing the right-handed twist. Interestingly, protegrin-1, a potent antimicrobial peptide displaying a right-handed twisted β-hairpin conformation^29^ (PDB ID: ; 1PG1), also shows the involvement of the guanidinium group of arginine at the *i*+2 site in side chain-backbone hydrogen bonding.

### Re-engineering a three-stranded β-sheet protein using the developed turn motifs

The enhanced thermodynamic stability of the monomeric β-hairpins in water is fundamentally attributed to the conformational preorganization of the β-turn attained by different *i*+1 and *i*+2 side-chain functional groups which imposes a favorable steric constraint on the β-turn. Such preorganization of the main chain torsion angles by side-chain substitution confers extraordinary conformational stability to proteins and peptides as was observed in collagen triple helices by substituting proline with 4-fluoroproline and 4-methylproline.^32^

**Fig. 6:**
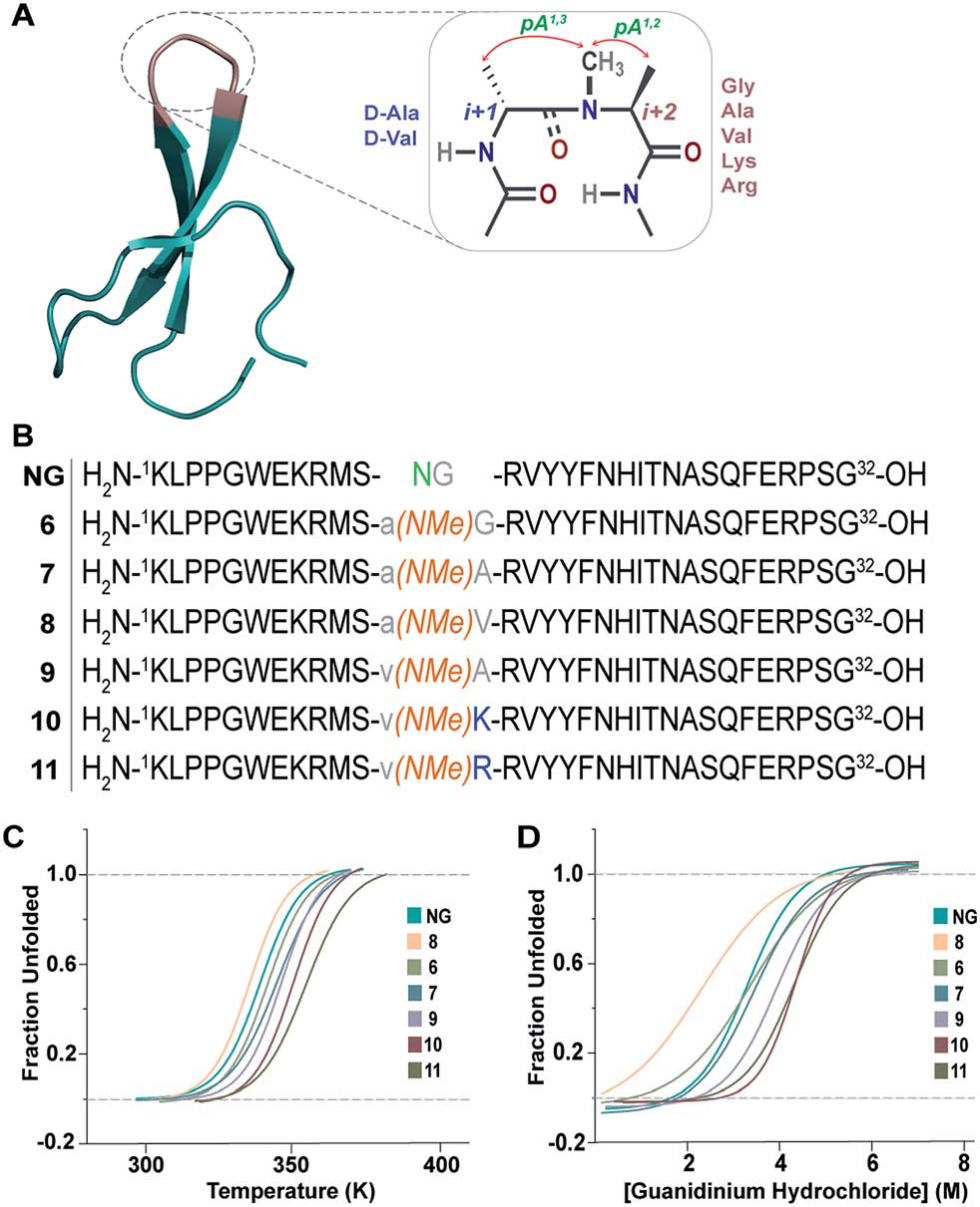
(A) Pin 1 WW domain with 2 residues (–NG–) in loop 1 (**NG**) (PDB ID: ; 1ZCN), which is re-engineered by introducing the pseudoallylic strain by incorporating different amino acids. (B) **NG** and the six-synthesized reverse-turn engineered Pin 1 variants. (C) Thermal unfolding of the Pin 1 variants monitored at 227 nm by variable temperature CD with 10 μM protein concentration in 20 mM sodium phosphate buffer, pH 7.0. The lines represent fits of the data to a two-state folding model.^30^ (D) Fluorescence-monitored guanidine hydrochloride denaturation curve of the Pin 1 variants with 2.5 μM protein concentration. The lines represent fits of the data to a two-state folding model.^31^

This encouraged us to utilize the designed turn motifs with myriad side-chain functional groups to engineer the conformational stability of proteins. Since antiparallel β-sheets are quite prevalent in proteins and have found great utility towards the development of non-antibody based- and hyperstable peptide scaffolds,^33^ we chose to incorporate our β-turn motifs into the three-stranded β-sheet protein, Pin 1 WW domain.^30^,^34^ The folding of the Pin 1 WW domain is rate-limited by the formation of loop 1 35 and it was shown by Kelly *et al.* that the introduction of a type II′ β-turn affords the fastest folding of the WW domain.^12^,^36^ Thus, we re-engineered the loop 1 in the Pin 1 WW domain to demonstrate the role of pseudoallylic strain in modulating the thermodynamic stability of proteins (Fig. 6A and B).

All the Pin 1 variants were chemically synthesized, which showed the characteristic maximum at 227 nm in CD spectra indicating the presence of a folded protein (ESI†). Their thermodynamic stability (Table 5) was assessed by thermal (Fig. 6C) and chemical (Fig. 6D) denaturation studies to obtain the *T*_M_ and free energy of folding (Δ*G*_f_), respectively. Unlike the model 12-mer peptide, the incorporation of *pA*^*1,3*^ strain at the reverse turn in **6** was sufficient to impart reasonable thermodynamic stability (*T*_M_ of 68.7 ± 0.2 °C) to the Pin 1 protein due to multiple hydrophobic interactions along the β-stands in the entire protein. The *T*_M_ values of all the Pin 1 variants studied were independent of the protein concentration over a range of 5–50 μM indicating their monomeric nature (ESI†). Furthermore, the incorporation of both *pA*^*1,3*^ and *pA*^*1,2*^ strain results in additional stabilization of the Pin 1 by ∼1.0 kcal mol^–1^ (ΔΔ*G*_f_) in **7** (Table 5). The introduction of a β-branched amino acid, valine, at the *i*+2 site (**8**) by virtue of the unfavorable *pA*^*1,2*^ strain destabilizes the Pin 1 by ∼0.9 kcal mol^–1^ (ΔΔ*G*_f_). However, when incorporated at the *i*+1 site (**9**), it stabilizes the protein by ∼1.2 kcal mol^–1^ (ΔΔ*G*_f_), further strengthening the critical role of steric interaction at the reverse turn in governing the thermodynamic stability of proteins. Nevertheless, the most exciting observation was the synergism between *i*+1 and *i*+2 residues at the reverse turn in enhancing the thermodynamic stability of the Pin 1 protein as observed in **10** (ΔΔ*G*_f_ of ∼1.6 kcal mol^–1^) and **11** (ΔΔ*G*_f_ of ∼1.9 kcal mol^–1^) with the incorporation of lysine and arginine at the *i*+2 site, respectively, which corroborated our findings in the 12-mer model peptide.

**Table 5:** Thermodynamic parameters of the reverse turn modified Pin 1 WW variants. ΔΔ*G*_f_ is compared across the pseudoallylic strain engineered variants only

	*T*_M_ (°C)	Δ*G*_f_ (kcal mol^–1^)	ΔΔ*G*_f_ (kcal mol^–1^)
**6**	68.7 ± 0.2	1.87 ± 0.04	0
**7**	72.0 ± 0.4	2.93 ± 0.06	–1.06
**8**	62.5 ± 0.4	0.97 ± 0.2	0.90
**9**	74.3 ± 0.3	3.10 ± 0.02	–1.23
**10**	78.4 ± 0.6	3.45 ± 0.01	–1.58
**11**	81.8 ± 0.4	3.77 ± 0.01	–1.90
**NG**	66.9 ± 0.3	1.75 ± 0.2	—

**Fig. 7:**
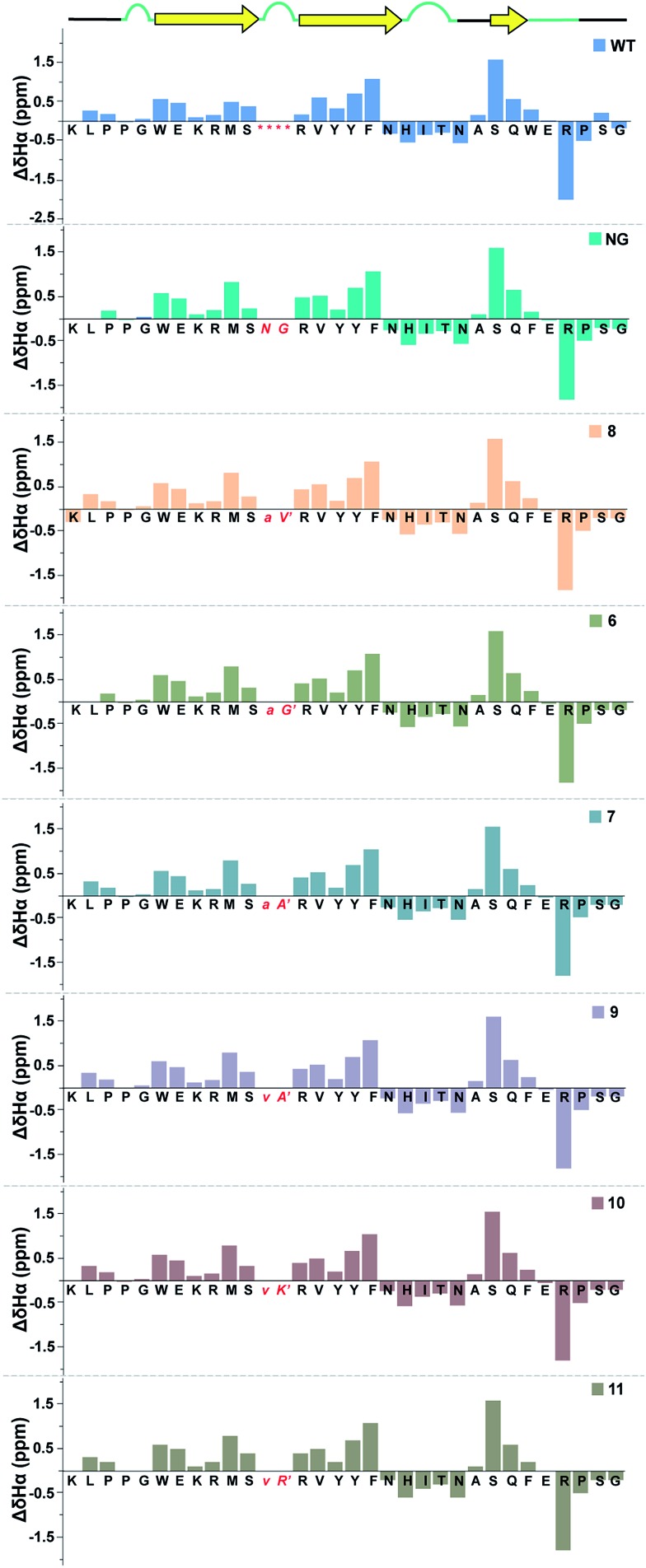
Comparison of backbone H^α^ chemical shift deviation of the apo-Pin 1 WW domain (**WT**) with the βI′ turn (**NG**) and the re-engineered Pin 1 variants deduced from the predicted random coil values,^37^ indicating minimal perturbation in the secondary structure. The –RSSG– motif in loop 1 of **WT** is indicated by an asterisk.

Next, we wondered whether this large change in thermodynamic stability between **8** and **11** (Δ*T*_M_ of ∼20 °C) corresponding to about 2.8 kcal mol^–1^ (ΔΔ*G*_f_) results from significant perturbation in the secondary structure of the re-engineered Pin 1. To identify the structural alterations in the solution conformation of Pin 1 variants, we acquired the ^1^H, TOCSY, and NOESY spectra of each Pin 1 variant (40–60 μM) at 15 °C. On comparing the backbone H^α^ CSD of the re-engineered Pin 1 variants with the wild-type apo-Pin 1 WW domain34*b* and the βI′ turn containing Pin 1 (**NG**)34*c* (Fig. 7), quite unexpectedly we observed that despite such a large change in thermodynamic stability, the native fold in all the re-engineered variants stays intact (ESI†). This was also validated by the characteristic NOEs and the stark upfield-shifted ^1^H resonances (ESI†). Collectively, these observations indicate the potential of the developed turn-inducing motifs in modulating the thermodynamic stability of proteins and enzymes without perturbing their native structure.^38^

### Enhanced global stability correlates with the increased local stability of the intramolecularly hydrogen bonded structure

Finally, we sought to investigate whether the conformational preorganization at the reverse turn eventually affects the global stability of the Pin 1 protein by stabilizing the folded conformation of the protein involved in intramolecular hydrogen bonds. Therefore, to qualitatively estimate the strength of the hydrogen bonds, we measured the amide temperature coefficients of the least and most stable Pin 1 variants **8** and **11**, respectively. It was quite satisfying to note that even at 35 °C, there was minimal change in the H^α^ chemical shift deviation in these variants indicating that the native fold was unperturbed (ESI†). This observation suggests the potential of this loop modification in engineering therapeutic proteins. Moreover, as there were no temperature induced chemical shift changes associated with the loss of the secondary structure of the protein, the amide temperature coefficients in these Pin 1 variants are good indicators of intramolecular hydrogen bonds.^25^,^39^

**Fig. 8:**
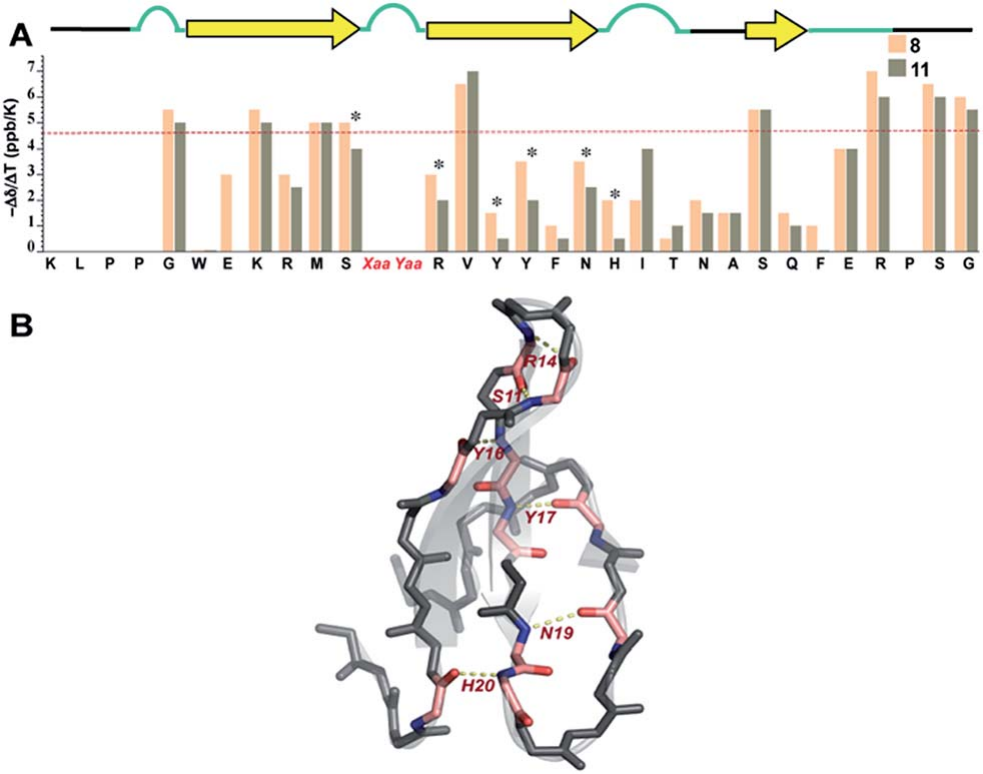
(A) Amide temperature coefficients of the least and most stable Pin 1 analogs **8** and **11**, respectively, obtained by analysing a series of TOCSY spectra acquired from 288 K to 308 K. The asterisks indicate the residues that show a change greater than or equal to 1 ppb K^–1^. The red dotted line marks the barrier at –4.6 ppb K^–1^, above which the values indicate the absence of intramolecular hydrogen bonding. (B) Residues showing a change greater than or equal to 1 ppb K^–1^ are highlighted in pink, which are mapped on Pin 1-NG (PDB ID: ; 1ZCN).

Overall, we observed more positive amide temperature coefficient values in **11** than **8** (Fig. 8A); however, we chose to analyze the amide protons that are involved in intramolecular hydrogen bonding.^40^ Interestingly, the most notable changes (difference in Δ*δ*/Δ*T* ≥ 1 ppb K^–1^ between the identical amino acids in **8** and **11**) were observed in Arg^14^ and Ser^11^ that are directly involved in stabilizing the reverse turn (loop 1) along with Tyr^16^ and Tyr,^17^ which are hydrogen bonded to the residues in N-term and C-term strands, respectively (Fig. 8B). Likewise, Asn^19^ and His^20^ that are hydrogen bonded to C-term and N-term strand residues, respectively, also displayed a stark increase. Thus, we note that most residues of the Pin 1 central strand in **11** show strong hydrogen bonding as compared to **8** to the amino acids of the flanking N-term and C-term strands indicating higher stability of the intramolecularly hydrogen bonded region in **11**.

## Conclusion

In summary, we demonstrate that the non-covalent constraint induced by *N*-methylation of a peptide bond differentially modulates the population of *cis*/*trans* isomers about the tertiary amide bond. This property of an *N*-methylated peptide bond along with its heavily restricted conformational space allowed us to probe the strength of pseudoallylic strain *pA*^*1,2*^ and *pA*^*1,3*^ in engineering reverse turns with different functional groups present on coded amino acids. Utilizing the combination of pseudoallylic strain and the functional groups on coded amino acids, we could impart remarkable conformational stability to isolated β-sheets and proteins in aqueous solution at room temperature. This enhanced conformational stability is presumably achieved by preorganization of the β-turn by the formation of stable hydrogen bonds at the reverse turn in the transition state.^23^,40b Such preorganization is achieved by incorporating diverse side-chain substituents at the *i*+1 and *i*+2 sites of a β-turn, which induces favorable steric constraints upon the main chain. Thus, by modifying the side-chain substituents at the turn, we could successfully tune the stability of β-sheets. Interestingly, a single amino acid substitution at the reverse turn also contributes towards modulating the right-handed twist in isolated β-sheets.

Since reverse turns are solvent exposed, and the pseudoallylic strain provided a basic conformational stability to the reverse turn, it allowed us to probe the effect of various amino acid substitution on the stability of a mini-protein, Pin 1 WW domain. We show that re-engineering the non-repetitive motif like β-turns with non-natural amino acids can have a large contribution to protein stability (ΔΔ*G*_f_ of ∼2.8 kcal mol^–1^ between the least and the most stable Pin 1 analogs). Hence, with the remarkable advancement in tools to generate semi-synthetic proteins^41^ and genetic techniques to incorporate noncanonical amino acids into proteins,^42^ we strongly believe that this protein engineering strategy will find its use in enhancing the thermodynamic stability of proteins,6c,^43^ enzymes^44^ and foldamers.^45^

## Conflicts of interest

There are no conflicts of interest to declare.

## Supplementary Material

Supplementary information
