# Supplementary material for: Protein stabilization by tuning the steric restraint at the reverse turn
Source: Chem Sci. 2018 Apr 24;9(20):4600–9. doi: 10.1039/c7sc05163h (PMC5969505; doi:10.1039/c7sc05163h)
Supplement: Supplementary file 1 [file SC-009-C7SC05163H-s001.pdf]

Electronic Supplementary Material (ESI) for Chemical Science.
This journal is © The Royal Society of Chemistry 2018

Supplementary Information

Protein stabilization by tuning steric restraint at the reverse turn

Priyanka Lahiri, Hitesh Verma, Ashraya Ravikumar, and Jayanta Chatterjee*

Molecular Biophysics Unit, Indian Institute of Science, Bangalore 560012, India

Table of Contents:

1. Materials and Methods …… S1-S7
2. *cis-trans* ratio of reported cyclic peptides …… S7-S11
3. HPLC retention times and HRMS …… S12-S13
4. ^{1}H NMR of L1-L4 …… S14
5. ^{1}H NMR dilution of **1-4** …… S15
6. Chemical shifts of **1-4** and their random coils …… S16-S18
7. ^{1}H NMR dilution of **3a-3n** …… S19-S22
8. ^{1}H NMR of **3a-3n** random coils …… S23
9. Chemical shifts of **3a-3n** and their random coils …… S24-S28
10. ^{1}H NMR dilution of **4a-4g** and **5** …… S29-S30
11. ^{1}H NMR of **4a-4g** and **5** random coils …… S31
12. ^{1}H NMR of fully folded **2, 3, 3a, 3g, 3n, 4a** and **5** …… S31
13. Chemical Shifts of Compound **pG** …… S32
14. Chemical shifts of **4a-4g, 5** and their random coils …… S33-S36
15. Chemical shifts of fully folded **2, 3, 3a, 3g, 3n, 4a** and **5** …… S37-S38
16. Coupling constants of **3n, 4a and 5** …… S38
17. Far-UV CD spectra of compounds **3, 3g, 3n, 4a** and **5**……S39
18. Chemical shift deviation of **3a-3n, 4a-4g** and **5** …… S36
19. Correlation of Amide temperature coefficients with ∑CSD of **3, 3a-3n** and **4a-4g,5**……S40
20. Variable temperature ^{1}H NMR of **2, 3, 3b, 3n, 4g, 5** ……S41-S46
21. ^{1}H NMR comparison of **2** and **4** at 328K …… S47
22. ^{1}H NMR of Pin1-WW variants **NG, 6-11** …… S48
23. ^{1}H NMR overlay of Pin1 variant **8** and **11** at 288 K and 308 K……S49
24. Chemical shifts of **6-11** …… S50-S51
25. Characteristic NOEs of **2, 3, 3a-3n, 4a-4g** and **5** ……S52-S53
26. NMR structure of **3n, 4a, 5a** and their NOE list with violation …… S54-S59
27. Ramachandran plot of *i+1* and *i+2* residues of **3n**, **4a** and **5**……S60
28. CD characterization of **6-11** …… S61
29. Thermal and chemical denaturation of **6-11** and **NG** …… S61-S64
30. Characteristic NOE of Pin1-WW analogs …… S65-S66
31. Chemical shift deviation of Pin1 analogs **8** and **11** at 288 K and 308 K……S66
32. Amide temperature coefficient values of Pin1 analogs **8** and **11**……S67
33. References …… S67

Materials and Methods:

General Information

All the Fmoc and orthogonally protected amino acids, (1[Bis(dimethylamino)methylene]-1*H*-1,2,3-triazolo[4,5-*b*] pyridinium 3-oxid hexafluorophosphate) (HATU), 1-hydroxy-7-azabenzotriazole (HOAt), 1-hydroxybenzotriazole (HOBt) were purchased from GL Biochem, Shanghai, China. 2-Chlorotrityl chloride polystyrene (2Cl-TCP) and Rink Amide AM resin were also purchased from GL Biochem, Shanghai, China. *N,N'*-Diisopropylcarbodiimide (DIC), *N,N*-diisopropylethylamine (DIPEA), Trifluoroacetic acid (TFA), Trifluoroethanol (TFE), Triisopropylsilane (TIPS), Triphenylphosphine, anhyd. tetrahydrofuran (THF), anhyd. methanol, Diisopropylazodicarboxylate (DIAD), 1,8-Diazabicyclo[5.4.0]undec-7-ene (DBU), 2-Mercaptoethanol, Glacial acetic acid, *N,N'*-Dicyclohexylcarbodiimide (DCC), N-methyl-2-pyrrolidone (NMP) , Thionyl Chloride, Calcium hydride, piperidine and Guanidium hydrochloride were purchased from Sigma-Aldrich. All the above reagents were used as commercially supplied. Solvents for RP-HPLC were purchased as HPLC grade and used without further purification. Dichloromethane was dried with Calcium hydride. All the other solvents were used as commercially supplied.

All the reactions were performed in oven-dried glass apparatus. Reactions on solid support were carried out in plastic syringes (10 ml) fitted with a frit column plate.

High-resolution mass spectra were recorded on a BrukerDaltonics ESI Q TOF- (Maxix Impact) with Nano LC (Proxeon easy nLC) mass spectrometer. ESI mass spectra were recorded in positive ion mode on aHCTultra ETD II ion trap spectrometer (PTM Discovery System, BrukerDaltonics, Germany). MALDI mass spectra were recorded on UltrafleXtreme TOF/TOF (BrukerDaltonics, Germany) and the data were processed and analysed using the Flex Analysis 3.1 software.

Nuclear magnetic resonance (NMR) spectra were recorded either on a 700 MHz BrukerAvance spectrometer (Bruker, Karlsruhe, Germany), or a 500 MHz Agilent NMR spectrometer.

Analytical RP-HPLC was performed on a Shimadzu UFLC system equipped with Prominence Diode Array (PDA) UV Detector at 210 nm, 254 nm and 270 nm using an analytical column (Phenomenex C18, 250 mm x 4.6 mm I.D., 5 µm) at a flow rate of 1 mL min^{-1}. Purifications were performed using a semi-preparative column (Phenomenex C18, 250 mm x 10 mm I.D., 5 µm) at a flow rate of 4 mL min^{-1}.

Circular Dichroism (CD) spectra were acquired on a JASCO-715 spectropolarimeter using 0.1cm path length cuvette. The CD spectra were averaged over 3 scans and the baseline correction was done by subtraction of the spectrum with the appropriate blank solution.

Fluorescence spectra were acquired on JASCO FP-6300 Spectrofluorometer using 0.1cm path length cuvette. The spectra were averaged over three scans.

Peptide Synthesis

Peptides **1** to **4, 3a-3n, 4a-4g, 5, pG** and their respective unfolded controls were synthesized on Rink Amide AM resin (0.8 mmolg^{-1}) on 200 mg scale (0.16 mmol) using standard Fmoc-based strategy. The resin was swollen in DMF and deprotected with 20% piperidine in DMF (5 min x 1, 15 min x 1) followed by thorough washing with DMF (3 times). The C-teminal amino acid, Fmoc-Gln(Trt)-OH (3.5 eq.) was loaded onto the resin by using standard coupling reagents (2.5 equiv HOBt, 2.5 equiv DIC) in DMF for 2 hours at room temperature. The entire peptide was assembled with this same protocol as well. The fully folded control of the respective peptides **pG**, **2**, **3**, **3a**, **3g**, **3n**, **4a** and **5** were assembled on Rink Amide AM resin similarly to its linear congeners with exception of the incorporation of Fmoc-Cys(Acm)-OH at both the N- and C-termini. The peptides were cyclized by oxidation using 4 equiv. of iodine in DMF for 2 h.

For Pin1 WW domain analogs, **NG**, **6** to **11** were synthesized on TCP resin (1.3 mmolg^{-1}), using standard Fmoc-based chemistry. The C-terminal amino acid residue, Fmoc-Gly-OH (1.25 equiv) was loaded on to the resin with 2.5 equiv DIPEA in anhydrous DCM (4 mL) at room temperature. After loading the first amino acid, the remaining unreacted trityl chloride groups bound to the solid support were capped using methanol (200 μl/100 mg resin) for 15 min. Next, the resin was thoroughly washed with DCM (3 times), 1:1 DCM-methanol (3 times) and methanol (3 times) and finally dried under vacuum. The loading capacity was estimated from the dry weight of the resin, which ranged from 0.4-0.6 mmolg^{-1}. The elongation of the rest of the peptide was performed on 150 mg (0.09-0.12 mmol) scale with DIC/HOBt as the coupling agents (2.5 equiv). Fmoc deprotections were carried out with 20% piperidine (5 min x 1, 15 min x 1) in DMF.

N-Methylation

A modified protocol for Mitsunobu reaction on the solid support was utilized for selective N-methylation of amino acid residue.[1]

Coupling of the amino acid residue following the N-methylated amino acid

Coupling of Fmoc-Xaa-OH to the free N^{α}-methylamine terminal of the peptides on the resin was carried out using 3 equiv each of HOAt, HATU and Fmoc-Xaa-OH and 6 equiv of DIPEA in DMF at room temperature.[1]

N-terminal Acetylation of the Peptide

After the final Fmoc deprotection of the peptides **1** to **4, 3a-3n, 4a-4g, 5, pG** and their respective unfolded and fully folded controls, the N-terminal was acetylated with acetic anhydride (2.5 equiv) and DIPEA (2.5 equiv) for 5 mins in DCM at room temperature. The resin was then washed thoroughly with DMF (5 times) followed by DCM (2 times).

Global Deprotection and Cleavage from the Resin

Peptides **1** to **4, 3a-3n, 4a-4g, 5, pG** and their respective unfolded and fully folded controls were cleaved off from the resin and globally deprotected by using the cleavage cocktail TFA:DCM:TIPS:H_2O (62.5:32.5:2.5:2.5) for 30 minutes at room temperature.

The cleaved peptide solution was then precipitated in chilled diethyl ether, centrifuged twice and dissolved in water for purification by RP-HPLC.

While Pin1 WW domain analogs **NG**, **6** to **11** were cleaved off from the resin and globally deprotected by using the cleavage cocktail TFA:DCM:TIPS: H_2O (62.5:32.5:2.5:2.5) for 30 minutes at room temperature. The cleaved peptide solution was then precipitated in chilled diethyl ether, centrifuged twice and dissolved in 10% acetonitrile for purification by RP-HPLC.

Purification By RP-HPLC

A suitably adjusted 20-minute gradient of 10% B to 50% B was used for purification of compounds **1** to **4, 3a-3n, 4a-4g, 5, pG** and their respective unfolded and fully folded controls, where solvent A was 0.1% TFA in H_2O and B was 0.1% TFA in acetonitrile. Pin1 WW domain analogs, **NG, 6** to **11** were purified using a 40-minute gradient of 15% B to 55% B, where solvent A was 0.1% TFA in H_2O and B was 0.1% TFA in acetonitrile.

Determination of Cis-Trans Equilibrium of N-methylated Tetrapeptides:

To evaluate the influence of pseudoallylic strain in governing cis-trans equilibrium in unrestrained N-methylated linear peptides, **L1-L4** tetrapeptides (Ac-V-Xaa-*NMe*Yaa-F-$CONH_2$) were synthesized on Rink amide AM resin using the standard Fmoc-based peptide synthesis as described above. N-methylation of these peptides were also carried out by the above described protocol. The final peptides were cleaved off from the resin using cleavage cocktail TFA:DCM:TIPS:H_2O (62.5:32.5:2.5:2.5) and precipitated in chilled water. The crude peptides were purified using a suitably adjusted 20-minute gradient ranging from 15% B to 60% B, where B was acetonitrile solvent with 0.1% TFA. Finally, the conformational dynamics of these unrestrained peptides were investigated by NMR (^1H, TOCSY and ROESY) in 100 mM sodium phosphate buffer (pH=3.8). Two conformations were observed for the peptides **L1-L4**. Population belonging to trans and cis conformations were obtained from the ROESY spectrum. Then, $K_{trans/cis}$ was calculated by integrating the peaks belonging to trans and cis conformation in proton spectrum of each tetrapeptides.

Generation of Ramachandran Maps:

The sterically allowed conformational space for the *i+1* and *i+2* residues in the tetrapeptides were explored by considering two-linked peptide unit systems within the tetrapeptides. A given two-linked peptide system starts from C_α atom of ith residue and ends with C_α atom of $i+2^{th}$ residue, including all the backbone atoms and C_β and H_α atoms attached to the middle C_α atom in case of alanine or two H_α atoms attached to the middle C_α atom in case of glycine. The conformational flexibility of these systems was obtained by computing their corresponding Ramachandran maps. Starting with the initial model structure, coordinates for every possible integral value of φ and ψ torsion angles, ranging from -180° to 180° were computed by performing rotations about the N-C_α bond and C_α-C bond. For every conformation, non-bonded inter-atomic distances between atoms which are at least three bonds away from each other were calculated. These distances were compared against a set of contact criteria given by Ramachandran *et al.*[2] For a given value of φ and ψ, if all the interatomic distances in the conformer are more than the normal limit, it is fully allowed; if one or more distances lie between the

normal and extreme limits, it is partially allowed; if one or more distances is lower than the extreme limit, it is disallowed.

Table S1. Contact criteria:

Type of contact	Normal Limit (in Å)	Extreme Limit (in Å)
H...H	2.0	1.9
H...O	2.4	2.2
H...N	2.4	2.2
H...C	2.4	2.2
O...O	2.7	2.6
O...N	2.7	2.6
O...C	2.8	2.7
N...N	2.7	2.6
N...C	2.9	2.8
C...C	3.0	2.9
C...C(H_3)	3.2	3.0
C(H_3)...C(H_3)	3.2	3.0

Table S1a. Standard distances obtained for N-methylated peptide from CCDC No. 278107.[3]

Bond Lengths

Type of contact	Bond Length (in Å)
N...CH_3	1.5
O=C...N-CH_3	1.4
CH_3 -N...C_α	1.5
C...O	1.2
C_α...C_β	1.5
C_α...H_α	1.0
C_β...H_β	1.0
C^N...H	1.0

Bond Angles

ω	180°

Circular Dichroism Spectroscopy

Far-UV CD spectra for compounds **1** to **4, 3a-3n, 4a-4g, 5, pG** were recorded at 125 μM concentration in 100 mM sodium acetate buffer (pH=3.8) over a wavelength range of 190-260 nm with a scan rate of 100 nm per minute and data pitch of 0.5 nm.

For Pin1 WW domain analogs **NG, 6** to **11**, Far-UV CD spectra were recorded at 10 μM concentration in 20 mM sodium phosphate buffer (pH=7) over a wavelength range of 190-260 nm with a scan rate of 100 nm per minute and data pitch of 0.5 nm.

Thermal stability of Pin1 WW variants (10 μM each) were assessed by monitoring the CD signal at 227 nm over a temperature range of 2-110°C with 0.5°C interval in 20 mM sodium phosphate buffer (pH=7). The samples were equilibrated for 2 minutes at each temperature and the data obtained was an average of three independent replicates. To monitor aggregation, thermal unfolding of the compounds were also recorded at two other concentrations, 5 μM and 50 μM. Values for T_M was extracted by nonlinear least square fit of thermal denaturation curve, assuming a two-state model as previously described.[4]

Fluorescence

Stability of Pin1 WW variants were assessed by chaotrope denaturation, where different concentrations of guanidinium hydrochloride (0-8 M) were titrated in 20 mM sodium phosphate buffer (pH=7.4) solution having equal protein concentration of 2.5 μM. The samples were equilibrated for 1.5 hours at 4°C and the fluorescence intensity were recorded. Emission spectra were recorded from 310 nm to 410 nm in 1 nm steps with excitation at 284 nm. Chemical denaturation curves were obtained by monitoring the maximum tryptophan fluorescence intensity at 338 nm as a function of guanidinium hydrochloride concentration. The data obtained were fit to a two-state model as previously described.[5]

Thermodynamic Determination of Compounds pG, 2, 3, 3a, 3g, 3n, 4a and 5:

The percentage of beta sheet population and ΔG_{fold} were calculated from the H_α chemical shifts for each peptide at the reporter residues (Val3, Val5, Lys8 and Ile10). These reporter residues are located at the hydrogen-bonded position which allows accurate determination of beta-sheet folded fraction. Fraction folded at each residue were calculated by the following equation;

$$\text{Percentage of Fraction Folded} = [(\delta_{obs}-\delta_U)/(\delta_F-\delta_U)]*100 \qquad \text{(Equation 1)}$$

Where, δ_{obs} is the H_α chemical shift of the peptide of interest, δ_U is the H_α chemical shift of its unfolded control in which the D-amino acid is replaced with the L-amino acid and δ_F is the H_α chemical shift of its fully folded control obtained by cyclizing the peptide through disulfide bond formation. The error in chemical shift assignment was considered as 0.01 ppm and was incorporated in the calculation by using the error propagation method.[6]

The equilibrium constant was calculated using Equation 2:

$$K = (\textit{Fraction Folded})/(1- \textit{Fraction Folded})$$

Finally, the ΔG_{fold} was calculated using Equation 3:

$\Delta G_{fold} = -RTlnK$

The ΔG_{fold} was calculated at each reporter positions and the average of the four values have been reported.

NMR Acquisition

For compounds **1** to **4, 3a-3n, 4a-4g, 5**, **pG,** the compounds were dissolved in 100 mM sodium acetate buffer (pH 3.8) H_2O:D_2O (9:1). In case of Pin1 WW analogs **6** to **11**, the compounds were dissolved in 20 mM sodium phosphate buffer (pH=7.4) having 10% D_2O. In all the compounds, 0.1% TMSP was used as an internal standard ($\delta = 0$ ppm). Standard Bruker pulse sequences zgesgp for ^1H, mlevesgpph/dipsi2rcesgpph (60 ms mixing time) for TOCSY, roesyesgpph (100 ms mixing time) for ROESY and noesyesgpph (200 ms mixing time) for NOESY were used to acquire the NMR data. Two-dimensional data were obtained using 2048 data points in the direct dimension and 512 data points in the indirect dimension

The NMR of compounds **1** to **4**, **3a-3n**, **4a-4g**, **5** and **pG** were obtained using concentration of 1-3 mM at room temperature (25°C). For Pin1 WW analogs **NG**, **6** to **11**, the NMR were obtained using a concentration of 40-60 μM at 15°C.

All NMR data were processed using iNMR (www.inmr.net), and the 2D NMR data were analyzed with SPARKY.[7] The chemical shift tables were generated from TOCSY and ^1H spectra. The sequential assignments and inter- and intra-residue NOEs were determined through ROESY. The NOEs were then integrated and the integration values were converted to distances using the formula $V=Kd^{-6}$, where V is the integrated peak volume, K is a constant (determined using resolved diastereotopic CH_2 groups from Tyr2 or in some cases Leu11), and d is the distance between the protons.

Determination of amide temperature coefficients of compounds **2**, **3**, **3a**, **3n**, **4**, **4a** and **5** were obtained by acquiring ^1H NMR spectrum at different temperature ranging from 298K to 328K and thereafter, the difference in Lys8 HN chemical shift per degree change in temperature were determined.

To monitor aggregation of compounds **1** to **4**, **3a-3n**, **4a-4g** and **5,** dilution experiments were performed. In this experiment, ^1H NMR spectra were recorded at 1:4 and 1:12 dilutions for each compound to observe any appreciable change in the proton shifts as a consequence of aggregation.

Determination of amide temperature coefficients of Pin1 compounds **8** and **11**, were obtained by acquiring series of TOCSY spectra ranging from 288-308 K with every 5 K increment in 20 mM sodium phosphate buffer, pH=7.4.

Structure Calculation

To calculate the structure of the molecule we have used charmM force field,[8] via the interface of Discovery Studio, for the entire process.

The distance restraints were converted into a charm restraint file using a custom Perl script. The resulting file was then used to define NOE restraints inside the charmM syntax. To the distance, 10% were added or subtracted to define the upper and lower limits respectively. If there were any methyl protons involved in the restraints, an additional 0.4Å per methyl group (pseudoatom correction) were added to the upper limit to compensate for the errors involved.[9]

For the compounds **3n**, **4g** and **5**, an explicit water box was used with water boundary of 16 Å. The linear structure was solvated in the water box. The solvated structure was refined by distance restraints to obtain an initial structure by the simulated annealing protocol. It was further refined by dihedral angle constraints derived from ^{1}H NMR spectra employing Bystrov equation[10] followed by a 10 ns restrained molecular dynamics run. The average over the dynamics run was considered to be the final structure and 10 lowest minimum energy structures were used to generate the ensemble.

Table S2. Cyclic N-methylated Peptides and their cis/trans population from reported literature. The table compiles the conformations of various reported model and bioactive N-methylated cyclic peptides and highlights the trans and cis peptide bonds with thick ‘green’ and ‘red’ bonds respectively. It’s interesting to note that the N-methylation induced pseudoallylic strain between heterochiral residues strictly favor a trans conformation (highlighted in ‘green’) while when present between homochiral residues there is equal probability of cis (highlighted in ‘red’) and trans conformations. The ratio of trans-cis reported here are observed in NMR time-scale.

1. *J. Am. Chem. Soc.* **2006**, *128,* 15164.

** The structures shown here are representative of the major conformers.

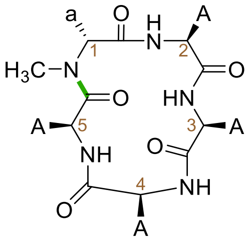	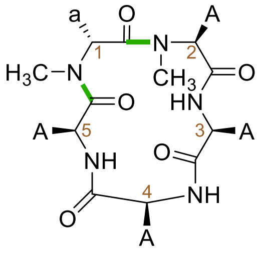	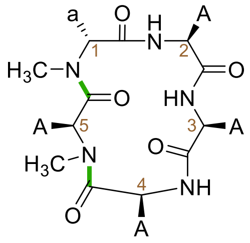	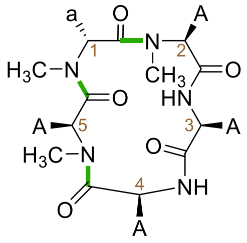
Compound 1 (all trans)	Compound 2 (all trans)	Compound 3 (all trans)	Compound 4 (all trans)
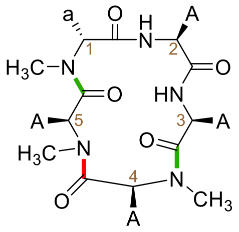	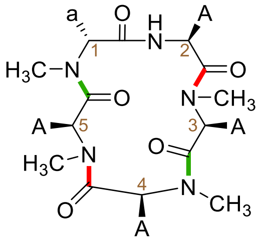	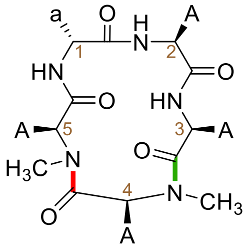	
Compound 5 (98:2)	Compound 6 (98:2)	Compound 7 (98:2)	

2. *Chem. Eur. J.* **2008**, *14,* 1508.

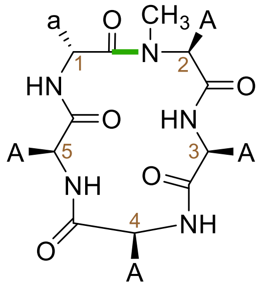	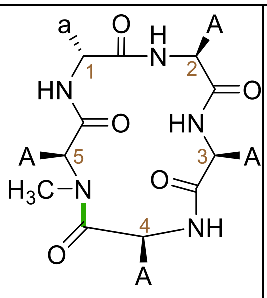	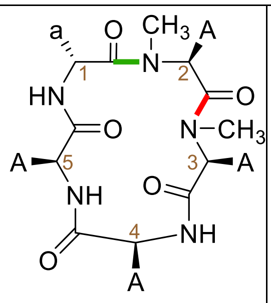	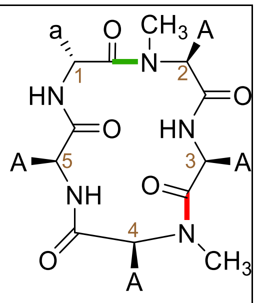
Compound 8 (85:11:4)	Compound 9 (all trans)	Compound 10 (83:12:5)	Compound 11 (84:11:5)
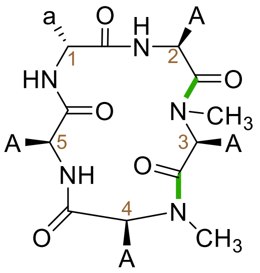	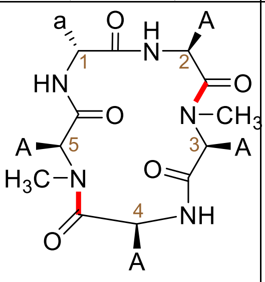	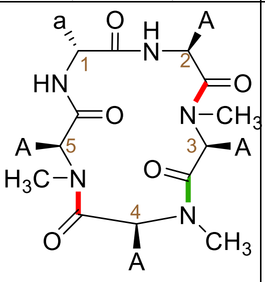	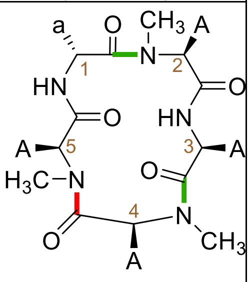
Compound 12 (82:12:6)	Compound 13 (87:10:3)	Compound 14 (85:10:5)	Compound 15 (84:16)
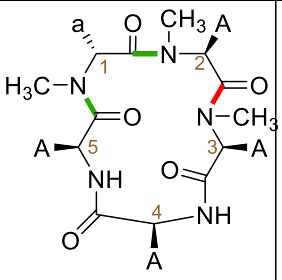			
Compound 16 (82:11:7)			

** The structures shown here are representative of the major conformers.

3. *J. Am. Chem. Soc.* **2012**, *134,* 12125.

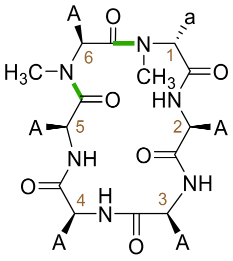	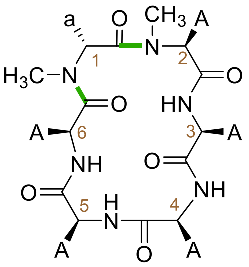	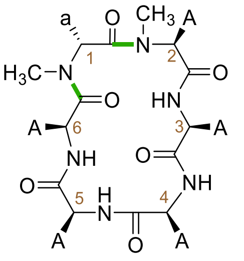	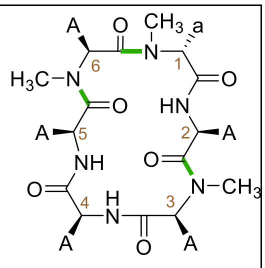
NMe(1,6) (all trans)	NMe(1,2) (all trans)	NMe(1) (all trans)	NMe(1,3,6) (all trans)

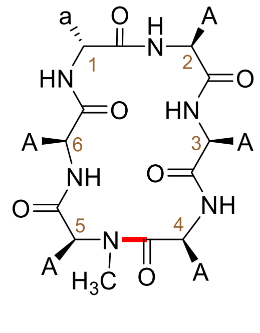	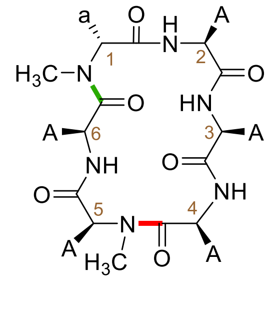	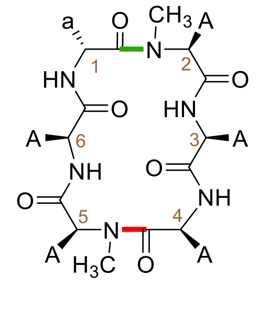	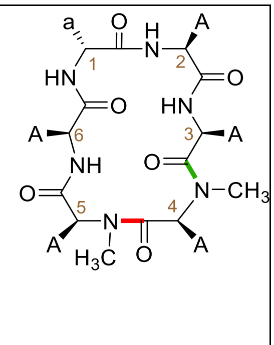
NMe(5)<br>(one cis)	NMe(1,5)<br>(one cis)	NMe(2,5)<br>(one cis)	NMe(4,5)<br>(one cis)
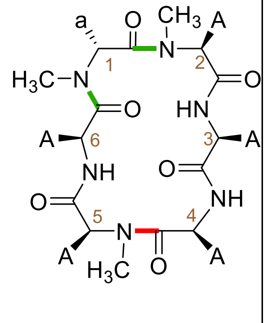	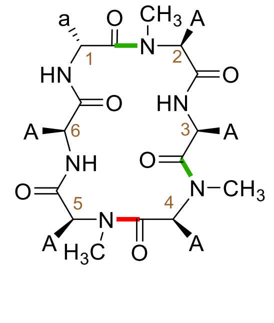	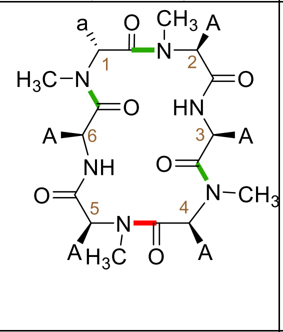	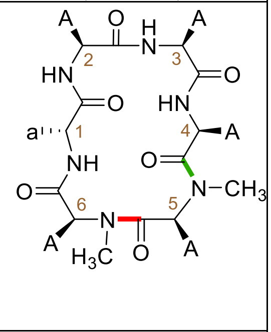
NMe(1,2,5)<br>(one cis)	NMe(2,4,5)<br>(one cis)	NMe(1,2,4,5)<br>(one cis)	NMe(5,6)<br>(58:42)
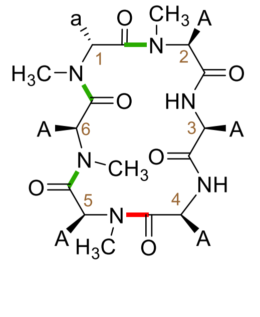	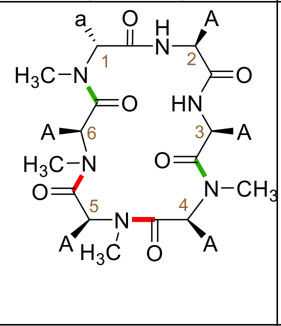	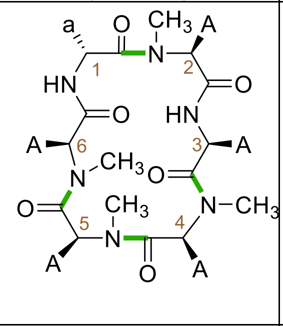	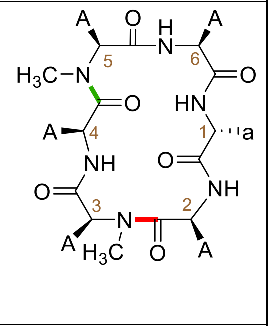
NMe(1,2,5,6)<br>(54:46)	NMe(1,4,5,6)<br>(53:47)	NMe(2,4,5,6)<br>(38:36:26)	NMe(3,5)<br>(cis 2-3: cis 2-3, cis 4-5)

** The structures shown here are representative of the major conformers.

4. *Angew. Chem. Int. Ed.* **2008**, *47,* 2595.

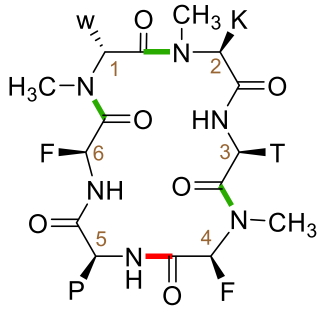
Analogue 8<br>*cyclo*(-PFMewMeKTMeF-)

5. *Proc. Natl. Acad. Sci. U. S. A.* **2014**, *111,* 17504.

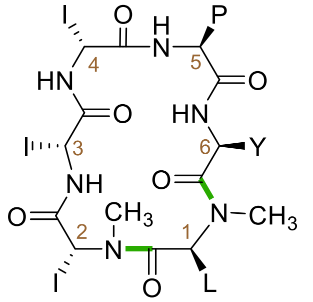

cyclo(-MeLMeIlIPY-)

6. *J. Med. Chem.* **1999**, *42,* 3033.

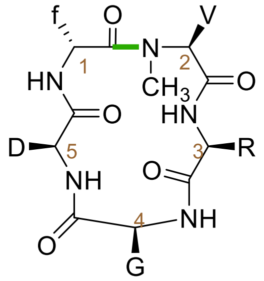

cyclo(-RGDfMeV-)

7. *J. Med. Chem.* **2007**, *50,* 5878.

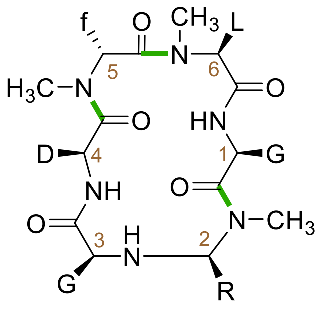

cyclo(-GMeRDMefMeL-)

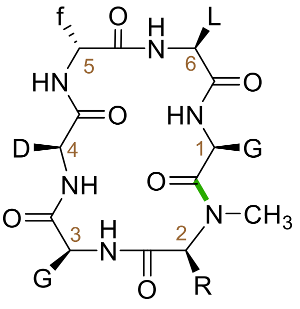

cyclo(-GMeRGDfL-)

8. *J. Am. Chem. Soc.* **2010**, *132,* 8115.

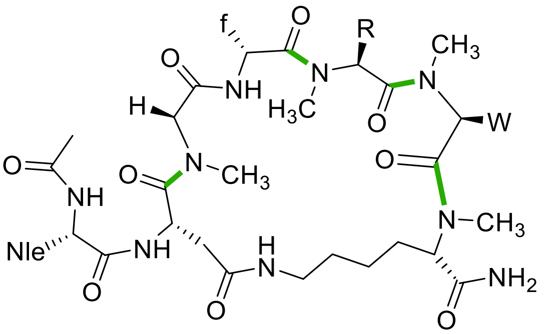

Ac-Nle-*cyclo*(-DMeHfMeRMeWMeK)-NH_2

9. *J. Med. Chem.* **2007**, *50,* 192.

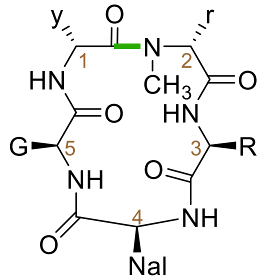

cyclo(-yMerRNalG-)

10. *J. Mex. Chem. Soc.* **2008**, *52,* 201.

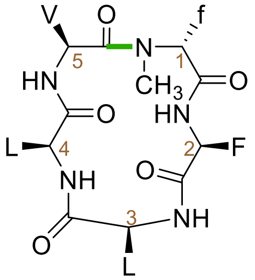

Sansalvamide A analogue 9

11. *J. Am. Chem. Soc.* **2015**, *137*, 315.

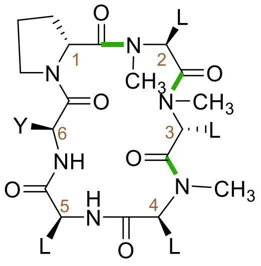

Compound 6.6

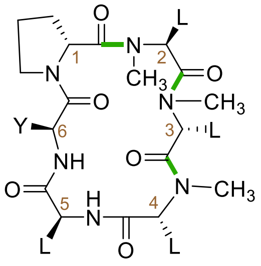

Compound 6.7

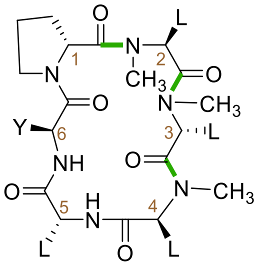

Compound 6.8

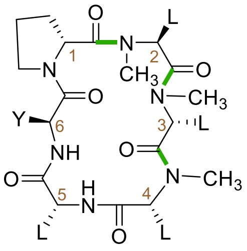

Compound 6.9

Table S3. Characterization of Compounds (HPLC retention time and HRMS).

Compound	Retention time	Expected mass	Observed mass			
	(in min)	[M+H]$^+$	[M+H]$^+$	[M+2H]$^{+2}$	[M+4H]$^{+4}$	[M+5H]$^{+5}$
**L1**	17.2	447.2482	447.2322			
**L2**	16.4	461.2638	461.2478			
**L3**	18.4	461.2638	461.547			
**L4**	16.7	447.2482	447.536			
**1**	15.9	1458.773	1458.9043	730.0015		
**2**	15.2	1458.773	1459.9105	730.5044		
**3**	16	1472.8	1473.9237	737.5094		
**4**	15.3	1458.773	1459.9088	730.5035		
**3a**	19.1	1514.881	1515.9723	758.5074		
**3b**	14.8	1500.854	1500.9519	751.508		
**3c**	14.8	1515.825	1516.9298	758.9864		
**3d**	15	1488.799	1489.9198			
**3e**	15.3	1502.826	1503.9433	752.5004		
**3f**	19.5	1514.881	1515.9784	758.5092		
**3g**	18.2	1548.898	1549.9571	775.494		
**3h**	18.5	1564.897	1565.958	783.484		
**3i**	15	1516.809	1516.9198	758.9824		
**3j**	15.3	1530.836	1531.9312	766.4844		
**3k**	18.7	1514.881	1515.9734	758.5076		
**3l**	14.5	1529.896	1531.3267			
**3m**	19.2	1529.852	1530.9503	765.9936		
**3n**	14.3	1557.91		780.0023		
**4a**	17.5	1514.881		758.4936		
**4b**	15.2	1516.809	1516.9124	759.4791		
**4c**	14.8	1502.826	1503.9365	752.4955		
**4d**	12.5	1529.896	1530.9802	766.0078		
**4e**	12.2	1564.897	1565.9575	783.4909		
**4f**	19.7	1548.898	1549.961	775.4933		
**4g**	17.8	1500.854	1501.9568			
**5**	16	1585.964	1587.028	794.0228		
**pG**	13.1	1471.7840	1471.8380			
**2-Cyc**	13.5	1691.089	1664.9183	832.9709		
**3-Cyc**	13.9	1677.062	1678.9364	839.9811		

**3b-Cyc**	14.7	1705.116	1706.9301	853.9694		
**3e-Cyc**	15.2	1707.088	1708.9457	854.9902		
**3n-Cyc**	15.1	1748.145	1765.0072	883.0266		
**4g-Cyc**	15	1705.116	1706.9679	854.0018		
**5-Cyc**	13.2	1776.199	1792.0316	896.535		
**pG-cyc**	19.4	1634.9930	1635.1240			
**2-Cont**	20	1458.773	1459.7966	730.4011		
**3-Cont**	15.5	1472.8	1473.8119	737.4069		
**3a-Cont**	17.4	1514.881	1515.8525	757.926		
**3b-Cont**	16.4	1500.854	1501.8484	751.4248		
**3c-Cont**	12.5	1515.825	1517.8173	758.9069		
**3d-Cont**	14.2	1488.799	1489.8042	745.4029		
**3e-Cont**	14	1502.826	1502.8156			
**3f-Cont**	18.6	1514.881	1515.8564	758.4288		
**3g-Cont**	20.8	1548.898	1549.8473	775.424		
**3h-Cont**	19.1	1564.897	1565.8277	783.418		
**3i-Cont**	12.6	1516.809	1516.8857			
**3j-Cont**	14.7	1530.836	1531.8162	766.4093		
**3k-Cont**	17.6	1514.881	1515.8564	758.4296		
**3l-Cont**	13.2	1529.896	1530.8721	765.9363		
**3m-Cont**	14.2	1529.852	1530.8362	765.9192		
**3n-Cont**	15.9	1557.91	1558.8792	779.9401		
**4a-Cont**	18	1514.881	1516.8655	758.9336		
**4b-Cont**	11.5	1516.809	1516.8799			
**4c-Cont**	13.2	1502.826	1503.8242	752.4137		
**4d-Cont**	11.9	1529.896	1530.8682	765.9354		
**4e-Cont**	13.2	1564.897	1564.9214			
**4f-Cont**	16.6	1548.898	1549.8411	775.4219		
**4g-Cont**	14.7	1500.854	1501.845	751.4234		
**5-Cont**	11.8	1585.964	1586.8992	793.9487		
**PG-Cont**	15.2	1471.7840	1471.8380			
**6**	20.1	3739.24			935.641	748.6235
**7**	23	3753.267			939.4165	751.7856
**8**	22.7	3781.321			1001.7214	801.635
**9**	23.4	3781.321			946.125	757.2051
**10**	22.4	3810.363			960.4125	768.6925
**11**	19.6	3838.377			967.4856	774.2984
**NG**	17.7	3769.2380			943.164	754.926

Tetra Peptide Library.

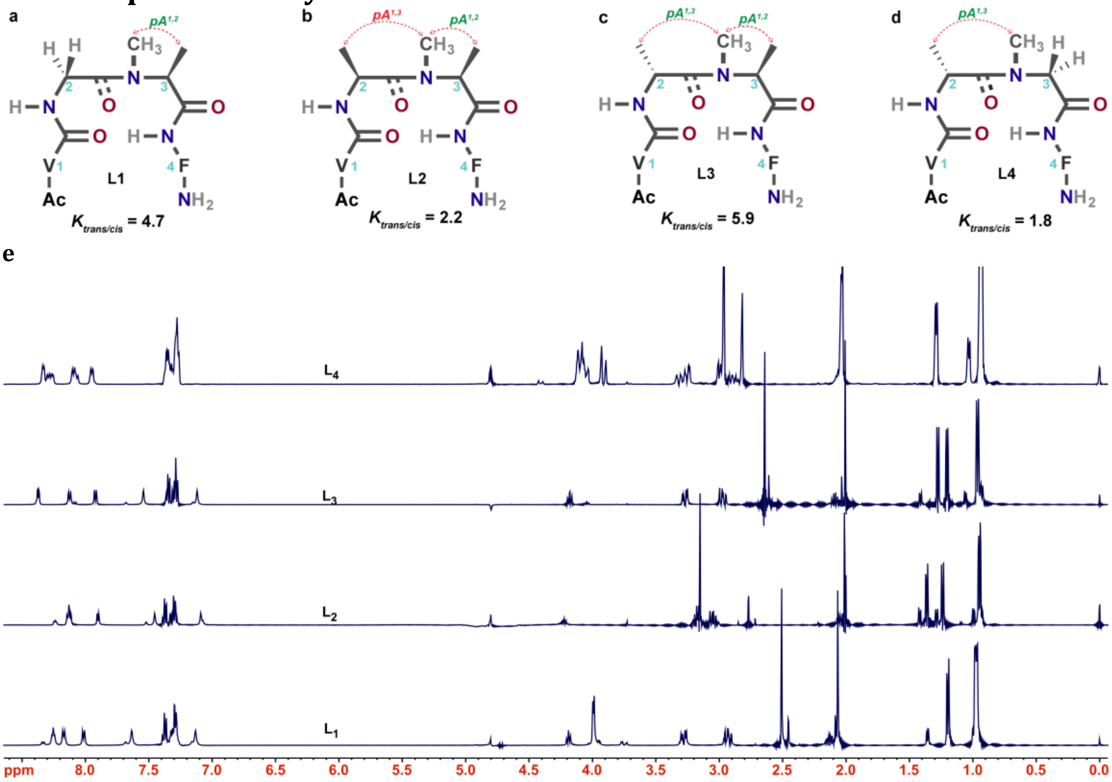

Figure S1. Linear tetrapeptides displaying varying $K_{trans/cis}$ determined by 1H NMR in acetate buffer (pH 3.8) at 25 °C. (a) Ac-V-G-(NMe)A-F-NH2; (b) Ac-V-A-(NMe)A-F-NH2; (c) Ac-V-a-(NMe)A-F-NH2; and (d) Ac-V-a-(NMe)G-F-NH2.
(e) ^{1}H NMR overlay of **L1-L4**.

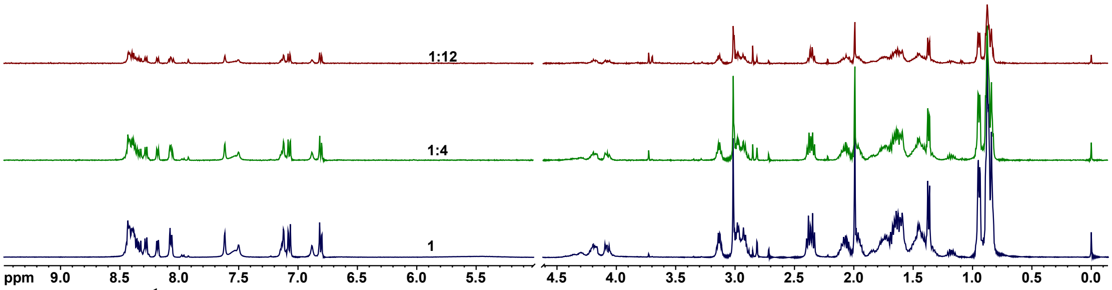

Figure S2. ^{1}H NMR overlay of Compound **1** at three different dilutions in 100 mM Sodium Acetate buffer (pH 3.8) at 298K.

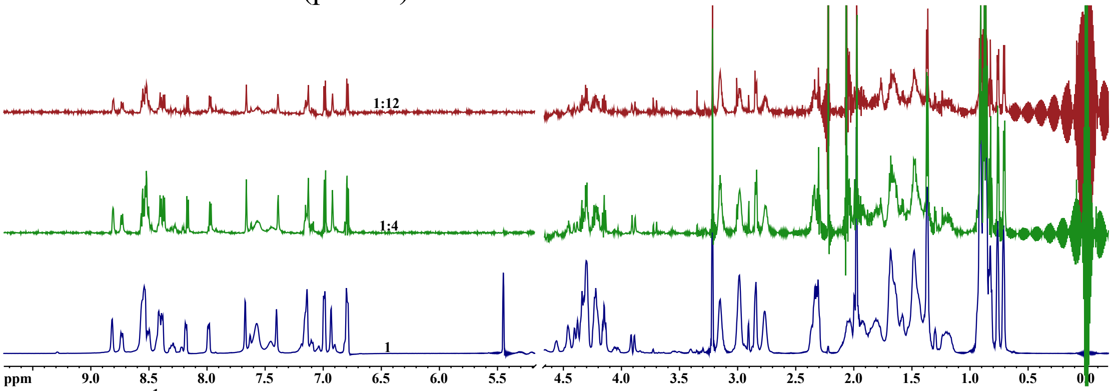

Figure S3. ^{1}H NMR overlay of Compound **2** at three different dilutions in 100 mM Sodium Acetate buffer (pH 3.8) at 298K.

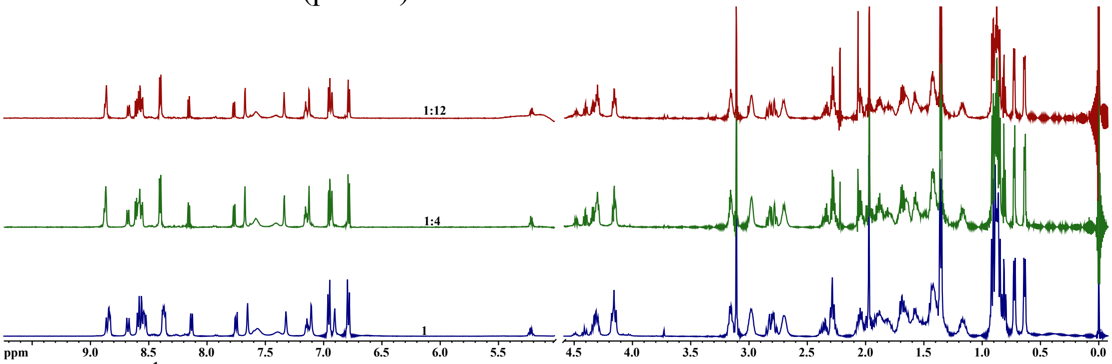

Figure S4. ^{1}H NMR overlay of Compound **3** at three different dilutions in 100mM Sodium Acetate buffer (pH 3.8) at 298K.

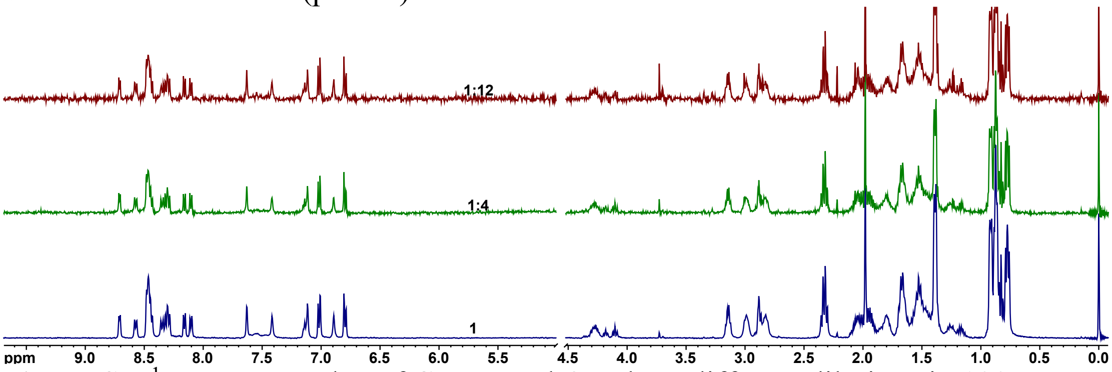

Figure S5. ^{1}H NMR overlay of Compound **4** at three different dilutions in 100mM Sodium Acetate buffer (pH 3.8) at 298K.

Table S4. Chemical Shifts of Compound **1-4** and **1-Cont – 4-Cont.**

			Chemical Shifts of Hairpin Peptides				Chemical Shifts of Control Peptides			
**Residues**			**1**	**2**	**3**	**4**	**1-Cont**	**2-Cont**	**3-Cont**	**4-Cont**
**Arg1**	**HN**		8.2	8.17	8.15	8.16		8.21	8.22	8.22
	**HA**		4.21	4.29	4.35	4.28	4.22	4.23	4.21	4.18
	**HB**	**1**	1.65	1.66						
		**2**			1.68	1.66				
	**HG**	**1**	1.48	1.57						
		**2**		1.5	1.51	1.5				
	**HD**		3.16			3.15				
	**NAc**		1.99	1.98	1.97	1.99				
**Tyr2**	**HN**		8.31	8.38	8.4	8.32		8.31	8.3	8.27
	**HA**		4.68	4.91	5	4.87	4.65	4.65	4.65	4.63
	**HB**	**1**	2.95	2.85	2.79	2.89				
		**2**								
**Val3**	**HN**		8.06	8.5	8.69	8.37		8.11	7.99	7.95
	**HA**		4.07	4.23	4.31	4.19	4.08	4.1	4.05	4.01
	**HB**		1.96		1.98	2.01				
	**HG**	**1**	0.87	0.87	0.86	0.88				
		**2**								
**Glu4**	**HN**		8.45	8.53	8.58	8.48		8.45	8.42	8.38
	**HA**		4.39	4.66	4.82	4.65	4.28	4.22	4.33	4.34
	**HB**	**1**				2.04				
		**2**				1.95				
	**HG**	**1**			2.31	2.33				

		**2**								
**Val5**	**HN**		8.36	8.56	8.59	8.45		8.42	8.3	8.25
	**HA**		4.17	4.14	4.16	4.11	4.13	4.13	4.12	4.04
	**HB**		2.08		1.95	2.01				
	**HG**	**1**	0.93	0.91	0.9	0.99				
		**2**								
**Xaa6**	**HN**		8.46	8.81	8.87	8.72				
	**HA**		4.09	4.76	4.77	4.26				
	**HB**			1.37	1.37	1.4				
**Xaa7**	**NMe/HN**		3.01	3.21	3.11	8.59				
	**HA**			3.91/4.36	5.22	4.31				
	**HB**	**1**	1.37		1.36	1.39				
		**2**								
**Lys8**	**HN**		8.1	7.97	7.76	8.11		8.19	8.12	8.3
	**HA**		4.28	4.47	4.49	4.37	4.3	4.36	4.24	4.25
	**HB**		1.76	1.78	1.81					
	**HG**									
	**HD**		1.41	1.43	1.4					
	**HE**							8.43	8.41	8.24
**Lys9**	**HN**		8.42	8.4	8.4	8.3	4.37	4.4	4.33	4.31
	**HA**		4.36	4.54	4.64	4.5				
	**HB**			1.65	1.62					
	**HG**				1.16					
	**HD**				1.36					
	**HE**									

<table>
<tr><td rowspan="6">Ile10</td><td>HN</td><td></td><td>8.37</td><td>8.73</td><td>8.88</td><td>8.59</td><td></td><td>8.44</td><td>8.35</td><td>8.26</td></tr>
<tr><td>HA</td><td></td><td>4.18</td><td>4.33</td><td>4.41</td><td>4.3</td><td>4.19</td><td>4.2</td><td>4.18</td><td>4.13</td></tr>
<tr><td>HB</td><td></td><td>1.84</td><td>1.87</td><td>1.87</td><td>1.87</td><td></td><td></td><td></td><td></td></tr>
<tr><td rowspan="2">HG</td><td>1</td><td rowspan="2">1.18</td><td rowspan="2">1.19</td><td rowspan="2">1.16</td><td>1.45</td><td></td><td></td><td></td><td></td></tr>
<tr><td>2</td><td>1.18</td><td></td><td></td><td></td><td></td></tr>
<tr><td>HD</td><td></td><td>0.92</td><td>0.9</td><td>0.88</td><td>0.87</td><td></td><td></td><td></td><td></td></tr>
<tr><td rowspan="7">Leu11</td><td>HN</td><td></td><td>8.44</td><td>8.53</td><td>8.56</td><td>8.47</td><td></td><td>8.47</td><td>8.45</td><td>8.38</td></tr>
<tr><td>HA</td><td></td><td>4.34</td><td>4.21</td><td>4.15</td><td>4.29</td><td>4.37</td><td>4.35</td><td>4.38</td><td>4.37</td></tr>
<tr><td rowspan="2">HB</td><td>1</td><td rowspan="2"></td><td>1.57</td><td>1.58</td><td rowspan="2">1.57</td><td></td><td></td><td></td><td></td></tr>
<tr><td>2</td><td>1.5</td><td>1.42</td><td></td><td></td><td></td><td></td></tr>
<tr><td>HG</td><td></td><td></td><td></td><td></td><td></td><td></td><td></td><td></td><td></td></tr>
<tr><td rowspan="2">HD</td><td>1</td><td rowspan="2"></td><td>0.76</td><td>0.72</td><td rowspan="2">0.79</td><td></td><td></td><td></td><td></td></tr>
<tr><td>2</td><td>0.71</td><td>0.62</td><td></td><td></td><td></td><td></td></tr>
<tr><td rowspan="6">Gln12</td><td>HN</td><td></td><td>8.42</td><td>8.56</td><td>8.61</td><td></td><td></td><td>8.05</td><td>8.41</td><td>8.35</td></tr>
<tr><td>HA</td><td></td><td>4.29</td><td>4.3</td><td>4.3</td><td></td><td></td><td></td><td></td><td>4.3</td></tr>
<tr><td rowspan="2">HB</td><td>1</td><td></td><td></td><td></td><td></td><td></td><td></td><td></td><td></td></tr>
<tr><td>2</td><td></td><td></td><td></td><td></td><td></td><td></td><td></td><td></td></tr>
<tr><td rowspan="2">HG</td><td>1</td><td></td><td></td><td></td><td rowspan="2"></td><td></td><td></td><td></td><td></td></tr>
<tr><td>2</td><td></td><td></td><td></td><td></td><td></td><td></td><td></td></tr>
</table>

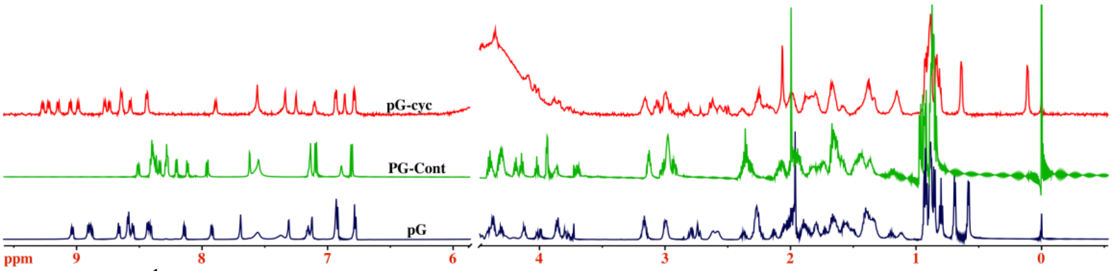

Figure S6. ^{1}H NMR overlay of Compound **pG, PG_Cont** and **pG-cyc** acquired in 100 mM Sodium Acetate buffer (pH 3.8) at 298K.

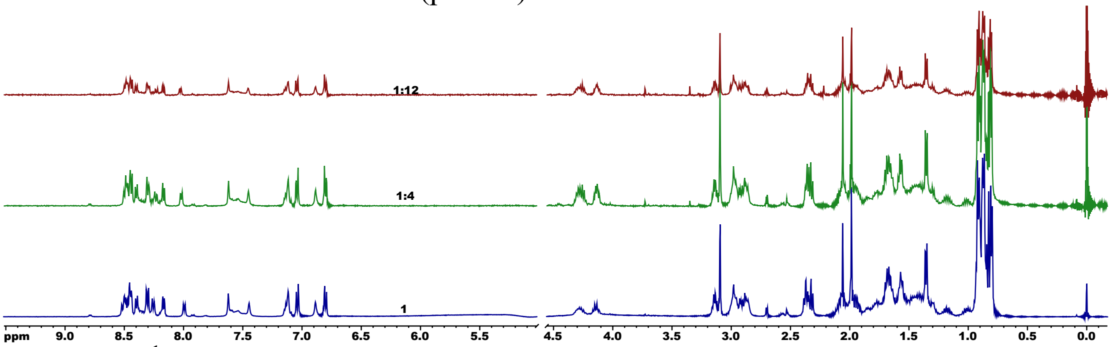

Figure S7. ^{1}H NMR overlay of Compound **3a** at three different dilutions in 100 mM Sodium Acetate buffer (pH 3.8) at 298K.

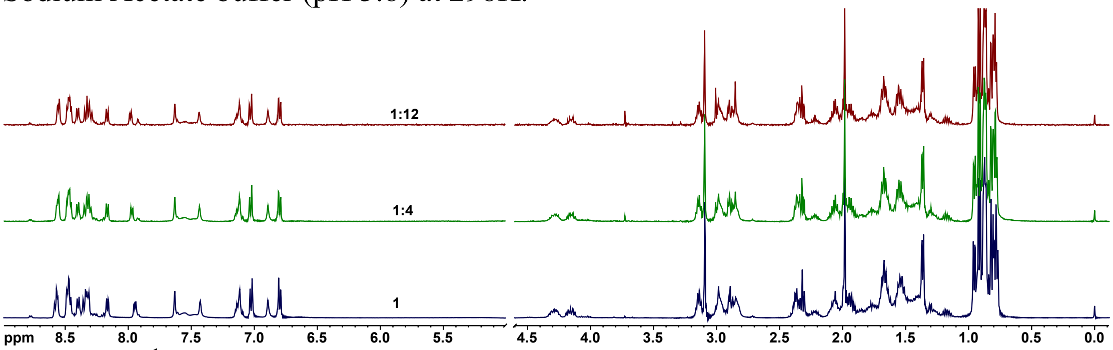

Figure S8. ^{1}H NMR overlay of Compound **3b** at three different dilutions in 100 mM Sodium Acetate buffer (pH 3.8) at 298K.

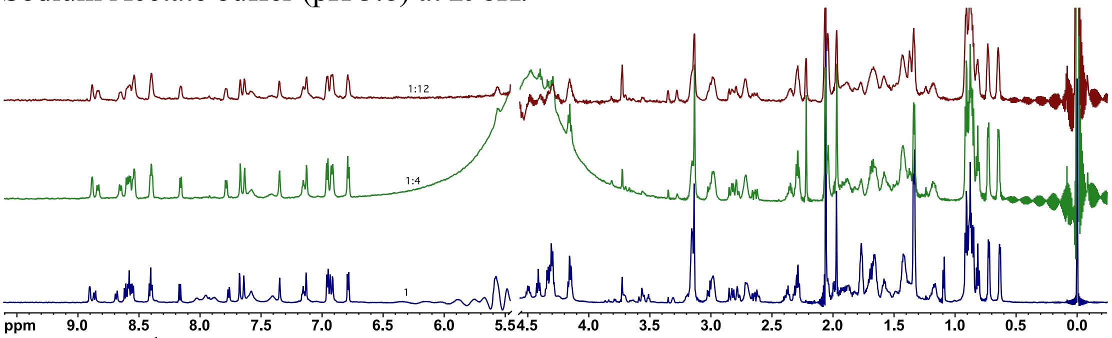

Figure S9. ^{1}H NMR overlay of Compound **3c** at three different dilutions in 100 mM Sodium Acetate buffer (pH 3.8) at 298K.

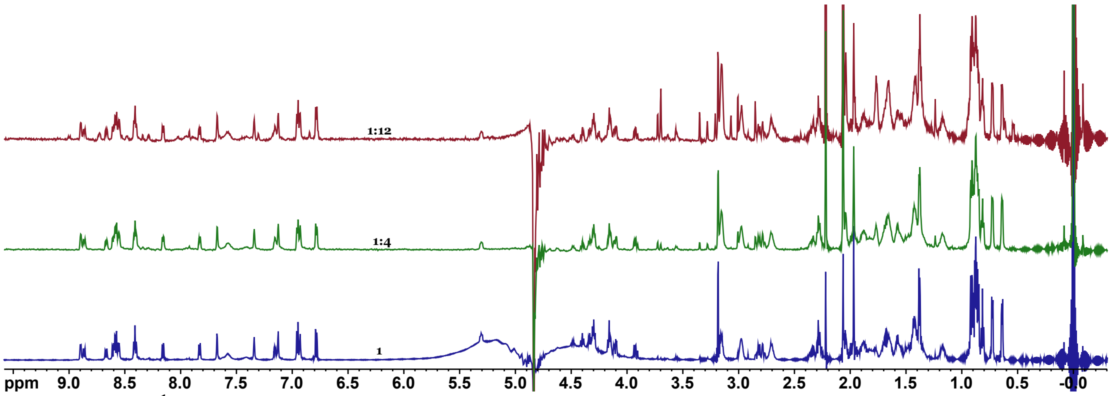

Figure S10.^{1}H NMR overlay of Compound **3d** at three different dilutions in 100 mM Sodium Acetate buffer (pH 3.8) at 298K.

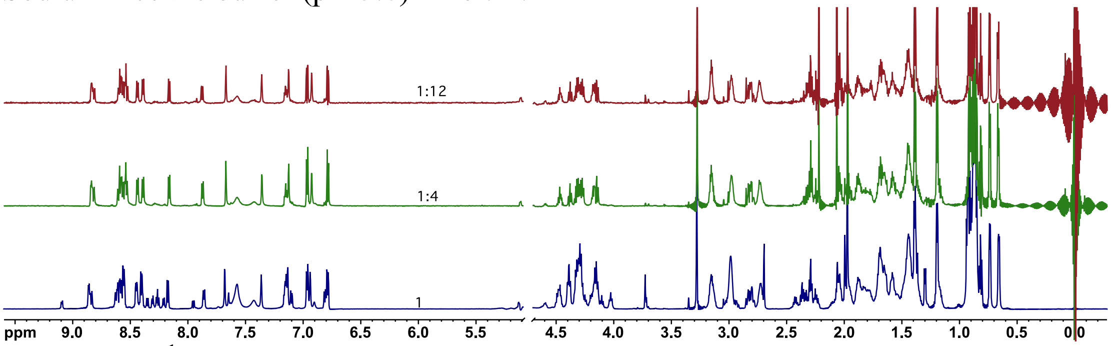

Figure S11. ^{1}H NMR overlay of Compound **3e** at three different dilutions in 100 mM Sodium Acetate buffer (pH 3.8) at 298K.

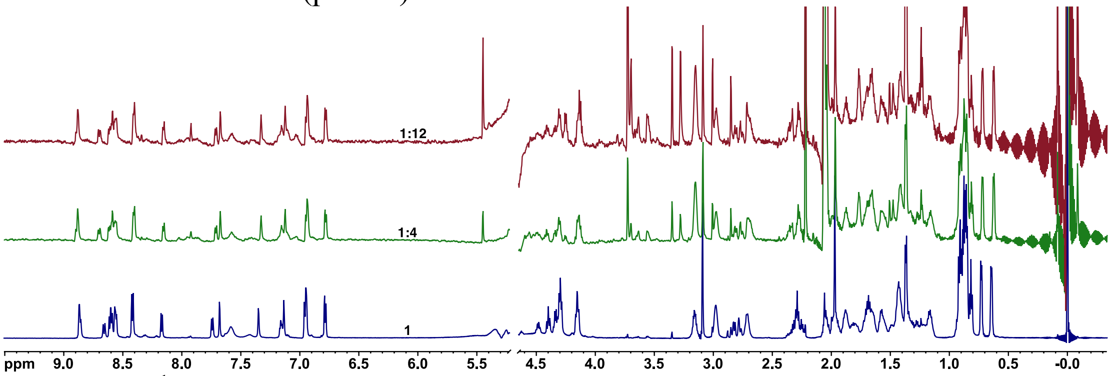

Figure S12.^{1}H NMR overlay of Compound **3f** at three different dilutions in 100 mM Sodium Acetate buffer (pH 3.8) at 298K.

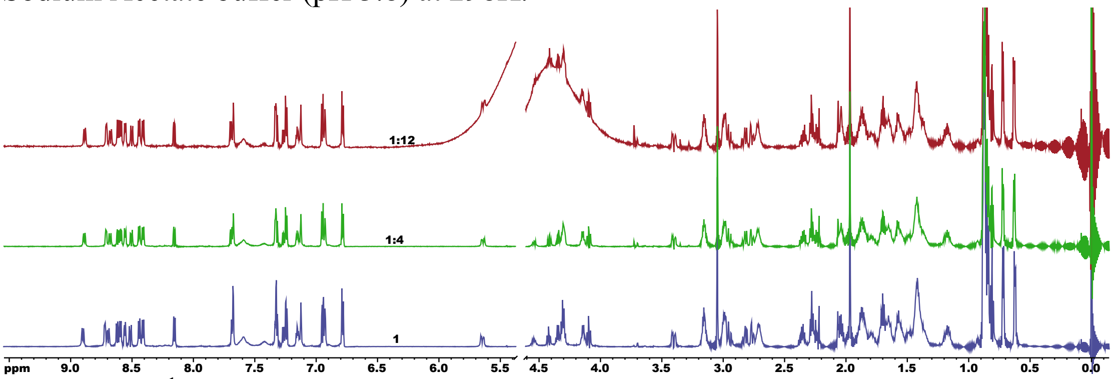

Figure S13. ^{1}H NMR overlay of Compound **3g** at three different dilutions in 100 mM Sodium Acetate buffer (pH 3.8) at 298K.

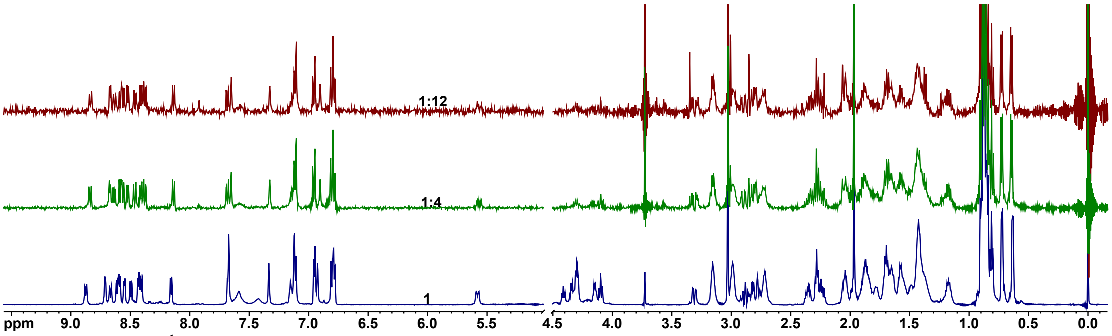

Figure S14. ^{1}H NMR overlay of Compound **3h** at three different dilutions in 100 mM Sodium Acetate buffer (pH 3.8) at 298K.

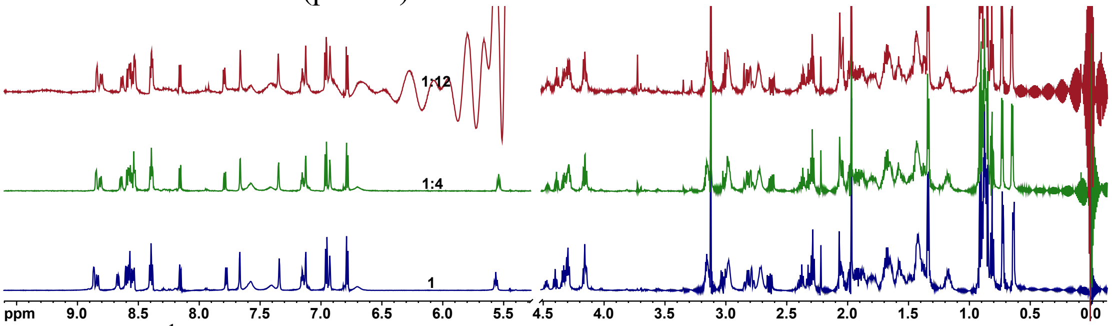

Figure S15. ^{1}H NMR overlay of Compound **3i** at three different dilutions in 100 mM Sodium Acetate buffer (pH 3.8) at 298K.

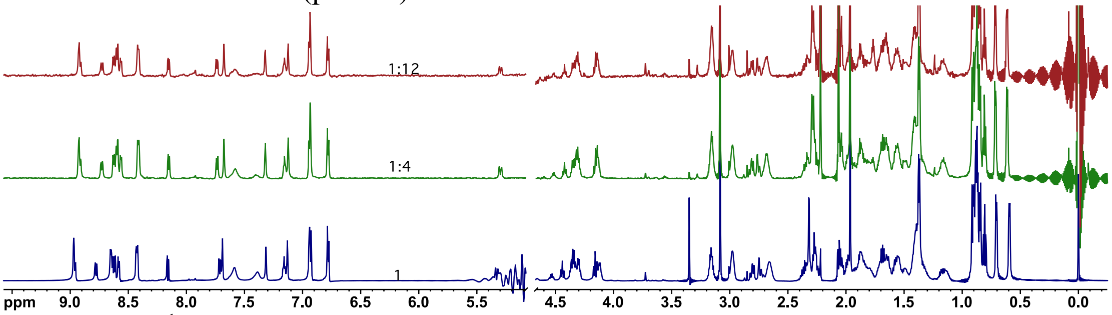

Figure S16. ^{1}H NMR overlay of Compound **3j** at three different dilutions in 100 mM Sodium Acetate buffer (pH 3.8) at 298K.

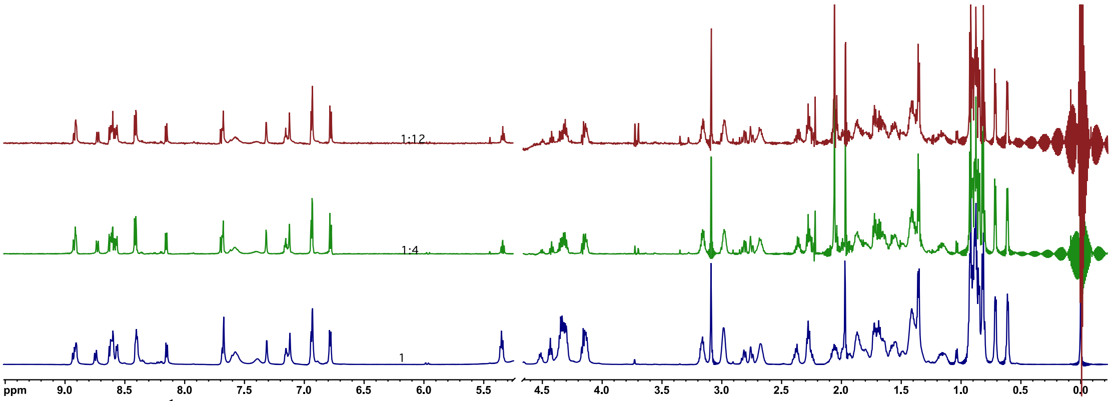

Figure S17. ^{1}H NMR overlay of Compound **3k** at three different dilutions in 100 mM Sodium Acetate buffer (pH 3.8) at 298K.

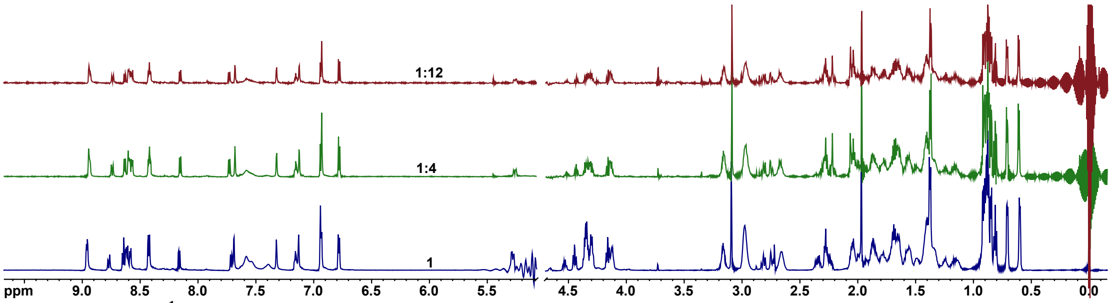

Figure S18. ^{1}H NMR overlay of Compound **3l** at three different dilutions in 100 mM Sodium Acetate buffer (pH 3.8) at 298K.

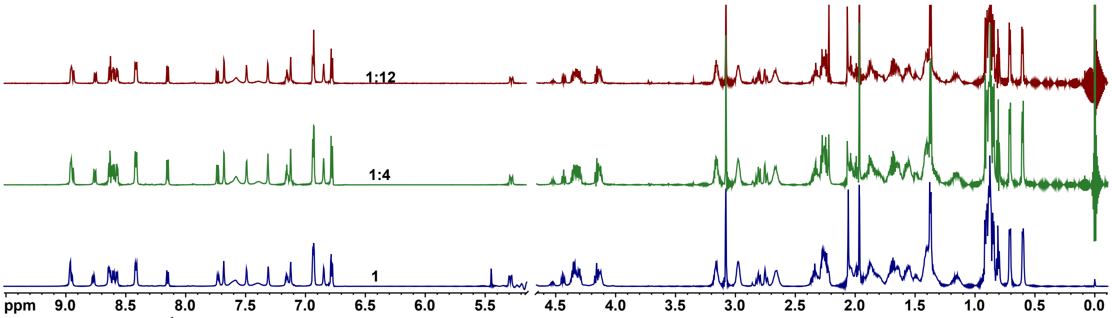

Figure S19. ^{1}H NMR overlay of Compound **3m** at three different dilutions in 100 mM Sodium Acetate buffer (pH 3.8) at 298K.

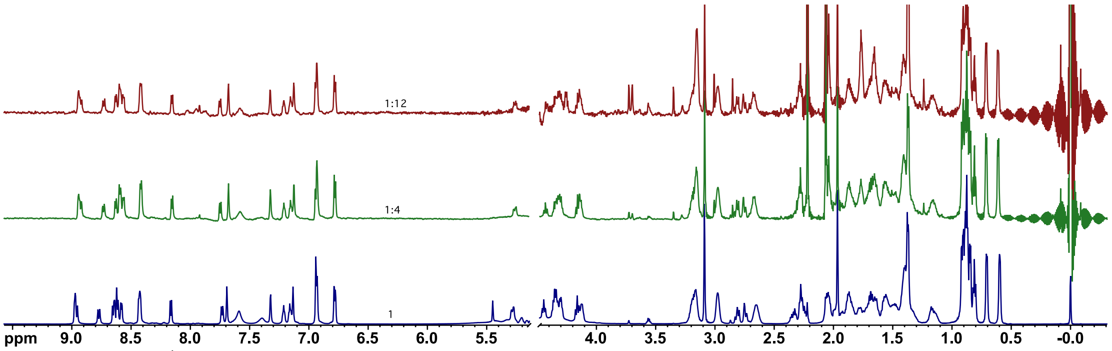

Figure S20. ^{1}H NMR overlay of Compound **3n** at three different dilutions in 100 mM Sodium Acetate buffer (pH 3.8) at 298K.

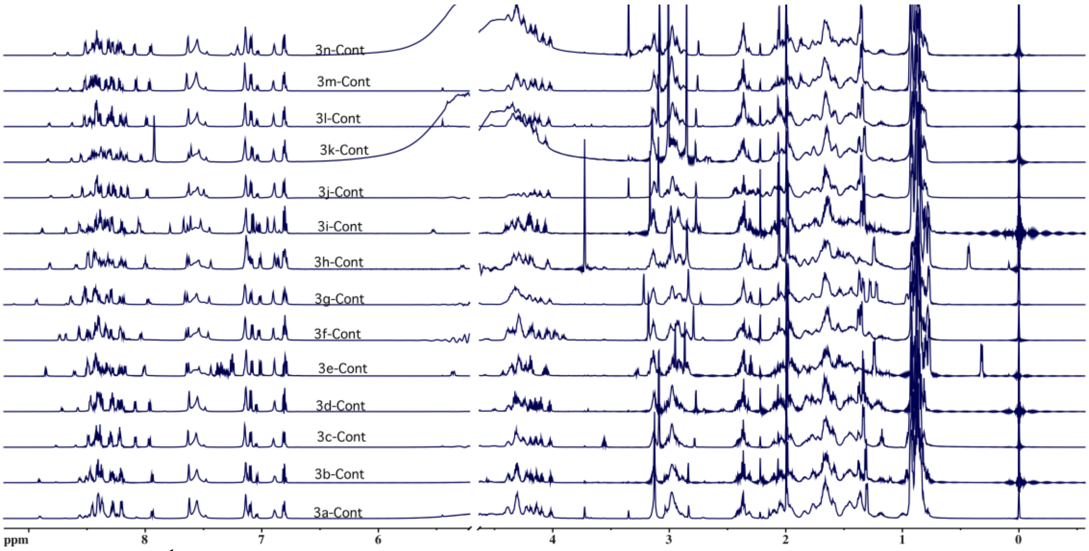

Figure S21. ^{1}H NMR overlay of Compound **3a-Cont – 3n-Cont** in 100mM Sodium Acetate buffer (pH 3.8) at 298K.

Table S5. Chemical Shifts of Compound **3a-3n.**

Residues			3a	3b	3c	3d	3e	3f	3g	3h	3i	3j	3k	3l	3m	3n
Arg1	HN		8.18	8.19	8.17	8.19	8.16	8.17	8.15	8.15	8.16	8.16	8.14	8.15	8.15	8.16
	HA		4.25	4.26	4.33	4.35	4.34	4.35	4.35	4.34	4.36	4.36	4.36	4.36	4.36	4.36
	HB	1	1.65	1.67	1.68	1.69	1.68	1.68	1.68	1.67	1.68	1.69	1.63	1.71	1.69	1.7
		2				1.65								1.64		1.66
	HG	1	1.53	1.5	1.52	1.55	1.52	1.49	1.51	1.51	1.53	1.55		1.54	1.53	1.56
		2				1.49								1.49		1.5
	HD					3.16	3.16	3.16				3.16	3.17	3.15	3.17	3.15
	NAc		1.98	1.98	1.97	1.97	1.97	1.97	1.97	1.97	1.97	1.96	1.97	1.97	1.97	1.96
Tyr2	HN		8.3	8.34	8.4	8.43	8.39	8.42	8.41	8.4	8.41	8.43	8.38	8.41	8.41	8.44
	HA		4.8	4.82	5.01	4.99	4.99	4.99	5	5	5.03	5.05	5.04	5.02	5.06	5.05
	HB	1	2.91	2.91	2.8	2.82	2.81	2.82	2.81	2.81	2.81	2.78	2.8	2.78	2.77	2.75
		2														2.81
Val3	HN		8.25	8.3	8.7	8.68	8.62	8.66	8.69	8.66	8.73	8.78	8.73	8.76	8.77	8.78
	HA		4.14	4.17	4.33	4.31	4.29	4.29	4.32	4.29	4.34	4.34	4.34	4.35	4.36	4.34
	HB		1.98	1.99	1.99		1.98	1.98	1.97	1.97	1.99	1.99	1.99	1.98	1.99	1.98
	HG	1	0.88	0.9	0.87	0.87	0.87	0.87	0.86	0.86	0.88	0.86	0.87	0.87	0.87	0.85
		2														
Glu4	HN		8.46	8.5	8.59	8.6	8.58	8.59	8.59	8.59	8.6	8.62	8.59	8.6	8.6	8.62
	HA		4.56	4.58	4.8	4.77	4.78	4.77	4.8	4.77	4.84	4.87	4.88	4.86	4.88	4.86
	HB	1		2.08			2.05	2.06	2.04	2.06		2.05	2.03		2.04	2.04
		2		1.93			1.89	1.89	1.88	1.87					1.88	

	**HG**	**1**		2.35	2.31		2.3	2.29	2.36	2.34		2.36	2.3		2.35	2.33
		**2**							2.24	2.23					2.25	
**Val5**	**HN**		8.31	8.36	8.58	8.56	8.54	8.56	8.51	8.49	8.62	8.64	8.59	8.62	8.63	8.64
	**HA**		4.13	4.15	4.17	4.17	4.16	4.15	4.09	4.1	4.18	4.16	4.17	4.16	4.16	4.17
	**HB**		2	2	1.93		1.95	1.94	1.9	1.91	1.93	1.89	1.95	1.92	1.93	1.88
	**HG**	**1**	0.92	0.93	0.91		0.91	0.91	0.89	0.88	0.92	0.9	0.91	0.89	0.91	0.9
		**2**														
**Xaa6**	**HN**		8.49	8.58	8.9	8.9	8.85	8.87	8.71	8.71	8.92	8.98	8.88	8.95	8.96	8.98
	**HA**		4.75	4.77	4.75	4.83	4.85	4.79	4.54	4.54	4.76	4.76	4.78	4.77	4.76	4.78
	**HB**		1.36	1.38	1.36	1.39	1.4	1.38		0.91	1.35	1.38	1.37	1.37	1.38	1.38
**Xaa7**	**NMe**		3.09	3.1	3.15	3.2	3.28	3.09	3.05	3.03	3.13	3.08	3.08	3.09	3.08	3.08
	**HA**		4.79	4.73	5.58	5.32	5.14	5.19	5.64	5.58	5.61	5.33	5.34	5.27	5.3	5.27
	**HB**	**1**	2.05	2.23	3.01	1.55	4.47	1.73	3.41	3.31	3.06	1.98	1.74	1.78	2	2.04
		**2**			2.65				2.95	2.89	2.68		1.43			1.75
	**HG**	**1**		0.84			1.2	1.32			3	2.32				1.5
		**2**														
	**HD**	**1**						1.16					0.92			3.18
		**2**											0.81			
**Lys8**	**HN**		7.99	8	7.77	7.89	7.85	7.74	7.68	7.68	7.76	7.72	7.69	7.71	7.73	7.74
	**HA**		4.29	4.32	4.5	4.5	4.5	4.49	4.55	4.53	4.52	4.54	4.52	4.45	4.53	4.53
	**HB**	**1**	1.74	1.77	1.8	1.79	1.8	1.79	1.8	1.81	1.8	1.79	1.82	1.78	1.82	1.81
		**2**										1.69				1.7
	**HG**															

	**HD**		1.38	1.41	1.4	1.42	1.41	1.41	1.43	1.4	1.41	1.41	1.42	1.41	1.41	1.4
	**HE**															
**Lys9**	**HN**		8.39	8.43	8.4	8.45	8.43	8.41	8.43	8.42	8.41	8.43	8.4	8.41	8.41	8.43
	**HA**		4.46	4.5	4.63	4.64	4.61	4.63	4.64	4.63	4.66	4.66	4.67	4.66	4.66	4.66
	**HB**	**1**	1.67	1.69	1.63	1.64	1.63	1.62	1.63	1.64	1.64	1.63	1.63	1.6	1.62	1.65
		**2**										1.55				1.56
	**HG**				1.18		1.17	1.16	1.17	1.17	1.17	1.14	1.14	1.13	1.13	1.14
	**HD**		1.32	1.34	1.36	1.35	1.37	1.38	1.38		1.35	1.34	1.37	1.33	1.35	1.36
	**HE**															
**Ile10**	**HN**		8.5	8.57	8.87	8.86	8.84	8.86	8.9	8.87	8.9	8.96	8.91	8.95	8.95	8.96
	**HA**		4.27	4.3	4.43	4.41	4.4	4.39	4.43	4.41	4.43	4.44	4.44	4.45	4.44	4.45
	**HB**		1.85	1.87	1.88	1.87	1.88	1.88	1.88	1.88	1.89	1.88	1.87	1.88	1.88	1.88
	**HG**	**1**	1.17	1.19		1.19	1.17	1.19	1.17	1.17		1.17	1.17		1.17	1.17
		**2**														
	**HD**		0.88	0.88	0.88	0.88	0.9	0.89	0.88	0.88	0.87	0.88	0.89	0.87	0.88	0.87
**Leu11**	**HN**		8.44	8.48	8.56	8.58	8.54	8.56	8.55	8.55	8.57	8.59	8.55	8.57	8.57	8.59
	**HA**		4.3	4.3	4.16	4.19	4.17	4.15	4.13	4.14	4.16	4.12	4.15	4.11	4.13	4.13
	**HB**	**1**	1.57	1.58	1.6	1.58	1.58	1.59	1.58	1.58	1.59	1.58	1.58	1.59	1.59	1.57
		**2**			1.44	1.48	1.45	1.44	1.42	1.43	1.43	1.41	1.41	1.41	1.41	1.4
	**HG**															
	**HD**	**1**	0.8	0.8	0.64	0.74	0.73	0.73	0.72	0.72	0.72	0.7	0.72	0.7	0.71	0.7
		**2**			0.72	0.66	0.65	0.64	0.62	0.63	0.62	0.6	0.61	0.61	0.59	0.6
**Gln12**	**HN**			8.49	8.61	8.63	8.59	8.6	8.62	8.61	8.63	8.65	8.62	8.64	8.64	8.66

	HA			4.32	4.31	4.32	4.31	4.3	4.31	4.3	4.31	4.3	4.31	4.3	4.31	4.31
	HB	1								2.06					2.03	2.05
		2								1.87					1.9	
	HG	1			2.29					2.28		2.27			2.28	2.26
		2											2.3			

Table S6. Chemical Shifts of Compound **3a-Cont – 3n-Cont.**

Compounds		3a-Cont	3b-Cont	3c-Cont	3d-Cont	3e-Cont	3f-Cont	3g-Cont	3h-Cont	3i-Cont	3j-Cont	3k-Cont	3l-Cont	3m-Cont	3n-Cont
Arg1	HN	8.2	8.2	8.2	8.21	8.21	8.19	8.2	8.19	8.21	8.22	8.22	8.23	8.2	8.21
	HA	4.18	4.18	4.21	4.24	4.22	4.2	4.19	4.19	4.2	4.27	4.2	4.2	4.19	4.2
Tyr2	HN	8.27	8.26	8.29	8.3	8.31	8.27	8.27	8.28	8.3	8.31	8.31	8.3	8.27	8.28
	HA	4.61	4.62	4.68	4.67	4.65	4.63	4.65	4.65	4.66	4.66	4.64	4.64	4.65	4.63
Val3	HN	7.94	7.93	8.05	8.04	7.99	7.96	8	7.98	8.04	8	7.97	7.98	7.97	7.95
	HA	4.01	4.01	4.07	4.06	4.04	4.03	4.05	4.04	4.06	4.06	4.04	4.03	4.04	4.02
Glu4	HN	8.4	8.37	8.42	8.43	8.42	8.4	8.39	8.38	8.43	8.43	8.43	8.43	8.4	8.4
	HA	4.33	4.3	4.4	4.38	4.31	4.35	4.35	4.31	4.38	4.37	4.32	4.31	4.36	4.31
Val5	HN	8.2	8.21	8.31	8.34	8.31	8.22	8.21	8.21	8.31	8.3	8.23	8.29	8.25	8.23
	HA	4.09	4.09	4.16	4.12	4.13	4.1	4.07	4.04	4.15	4.14	4.11	4.1	4.11	4.09
Xaa6	HN	*	8.47	8.56	8.57	8.54	8.46	8.3	8.3	8.55	8.53	8.5	8.53	8.54	8.51
	HA	*	4.33	4.7	4.77	4.86	4.8	4.65	4.68	4.74	4.79	4.8	4.75	4.74	4.76
Yaa7	HN	*	2.99	3.16	3.18	3.21	3.09	*		*	3.09	3.09	3.08	3.09	3.08
	HA	*	*	5.53	*	*	*	*	5.28	*	*	5.03	4.92	*	*
Lys8	HN	8.38	8.18	8.05	8.19	8.43	8.08	8	8	8.16	8.18	8.1	8.09	8.15	8.09

	**HA**	4.22	4.21	4.22	4.22	4.26	4.25	4.24	4.23	4.2	4.27	4.27	4.26	4.25	4.24
**Lys9**	**HN**	8.41	8.42	8.34	8.4	8.44	8.38	8.36	8.36	8.35	8.42	8.4	8.47	8.42	8.45
	**HA**	4.31	4.31	4.34	4.31	4.33	4.31	4.33	4.32	4.34	4.34	4.32	4.32	4.32	4.32
**Ile10**	**HN**	8.29	8.28	8.28	8.35	8.34	8.28	8.33	8.31	8.25	8.32	8.32	8.35	8.31	8.31
	**HA**	4.14	4.14	4.19	4.17	4.16	4.15	4.18	4.16	4.18	4.18	4.16	4.16	4.16	4.15
**Leu11**	**HN**	8.4	8.4	8.38	8.43	8.44	8.4	8.43	8.42	8.39	8.43	8.44	8.45	8.41	8.42
	**HA**	4.38	4.37	4.35	4.36	4.39	4.37	4.37	4.38	4.35	4.39	4.39	4.39	4.37	4.38
**Gln12**	**HN**	*	*	8.38	8.41	8.53	8.36	*	8.49	8.38	8.4	8.39	8.41	8.37	*
	**HA**	*	*	4.29	4.31	4.33	4.3	*	4.29	4.3	4.32	4.31	4.3	4.3	*

Note: '*' indicate the missing chemical shifts of the respective residue due to water suppression or peak overlap.

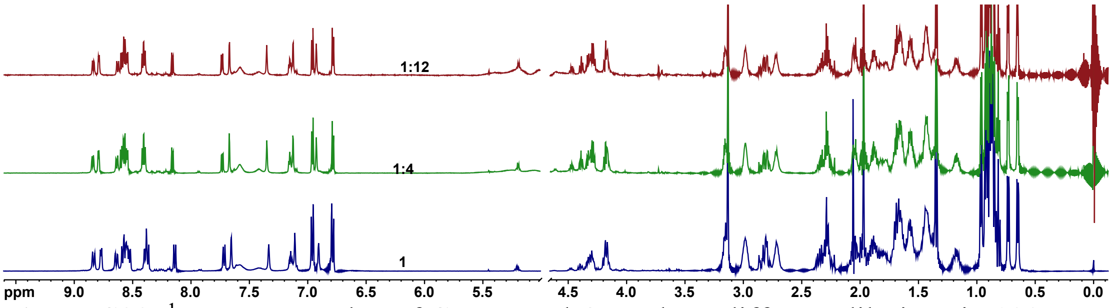

Figure S22. ^{1}H NMR overlay of Compound **4a** at three different dilutions in 100 mM Sodium Acetate buffer (pH 3.8) at 298K.

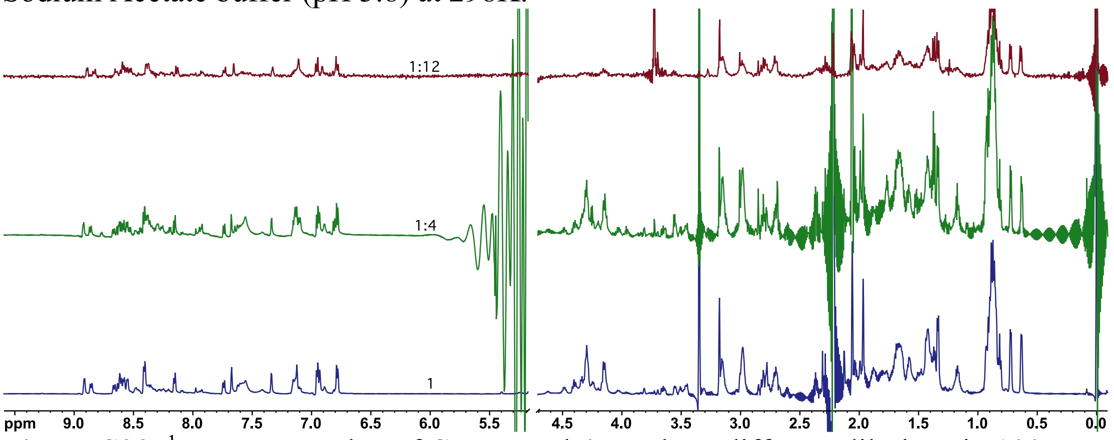

Figure S23. ^{1}H NMR overlay of Compound **4b** at three different dilutions in 100 mM Sodium Acetate buffer (pH 3.8) at 298K.

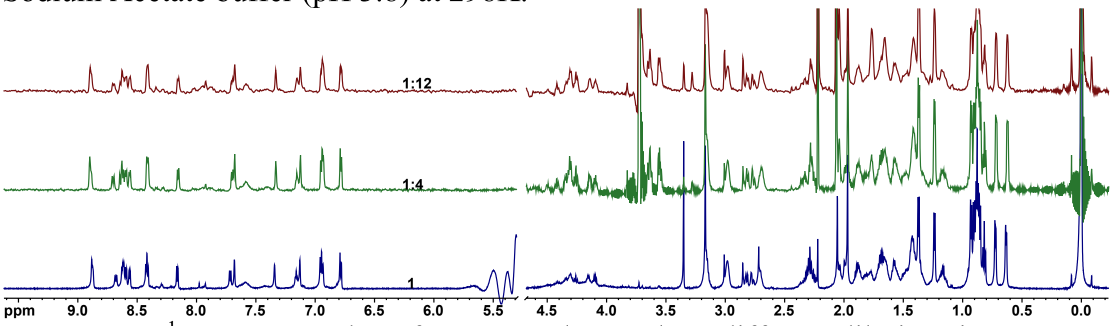

Figure S24. ^{1}H NMR overlay of Compound **4c** at three different dilutions in 100 mM Sodium Acetate buffer (pH 3.8) at 298K.

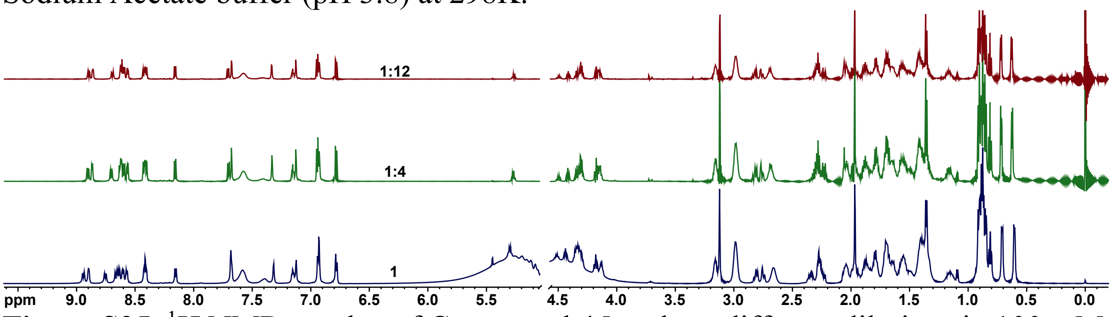

Figure S25. ^{1}H NMR overlay of Compound **4d** at three different dilutions in 100 mM Sodium Acetate buffer (pH 3.8) at 298K.

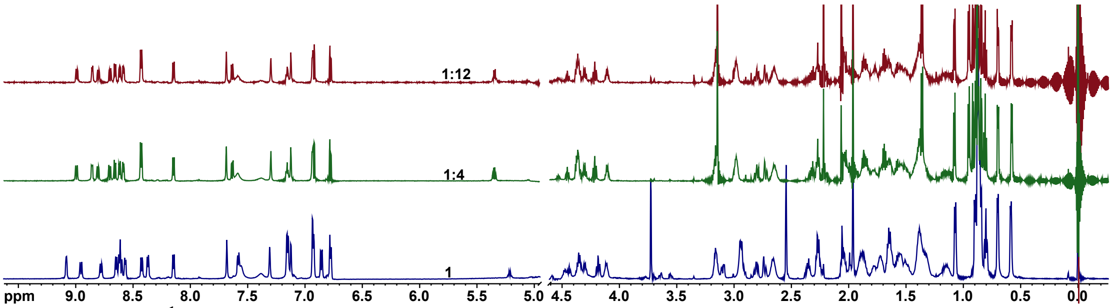

Figure S26. ^{1}H NMR overlay of Compound **4e** at three different dilutions in 100 mM Sodium Acetate buffer (pH 3.8) at 298K.

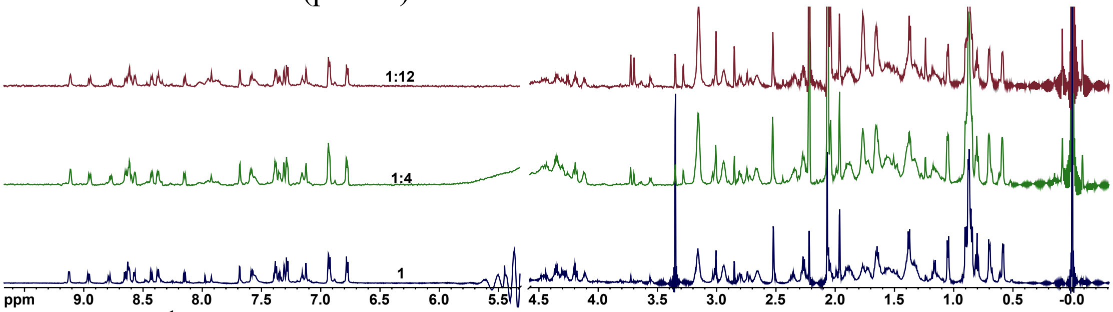

Figure S27. ^{1}H NMR overlay of Compound **4f** at three different dilutions in 100 mM Sodium Acetate buffer (pH 3.8) at 298K.

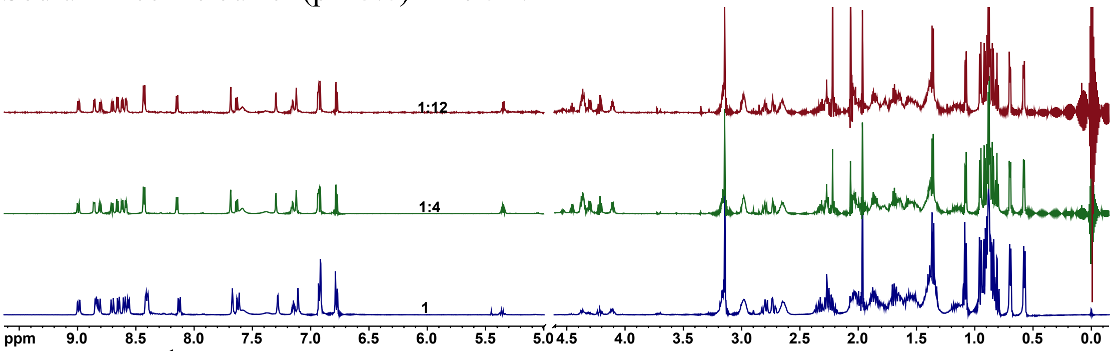

Figure S28. ^{1}H NMR overlay of Compound **4g** at three different dilutions in 100 mM Sodium Acetate buffer (pH 3.8) at 298K.

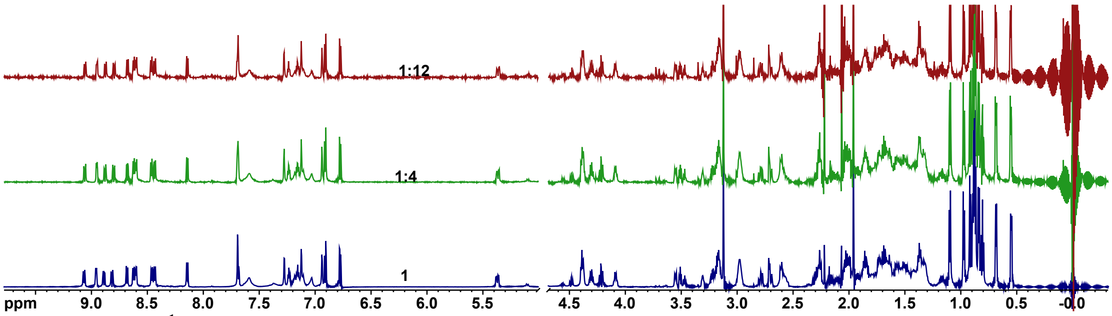

Figure S29. ^{1}H NMR overlay of Compound **5** at three different dilutions in 100 mM Sodium Acetate buffer (pH 3.8) at 298K.

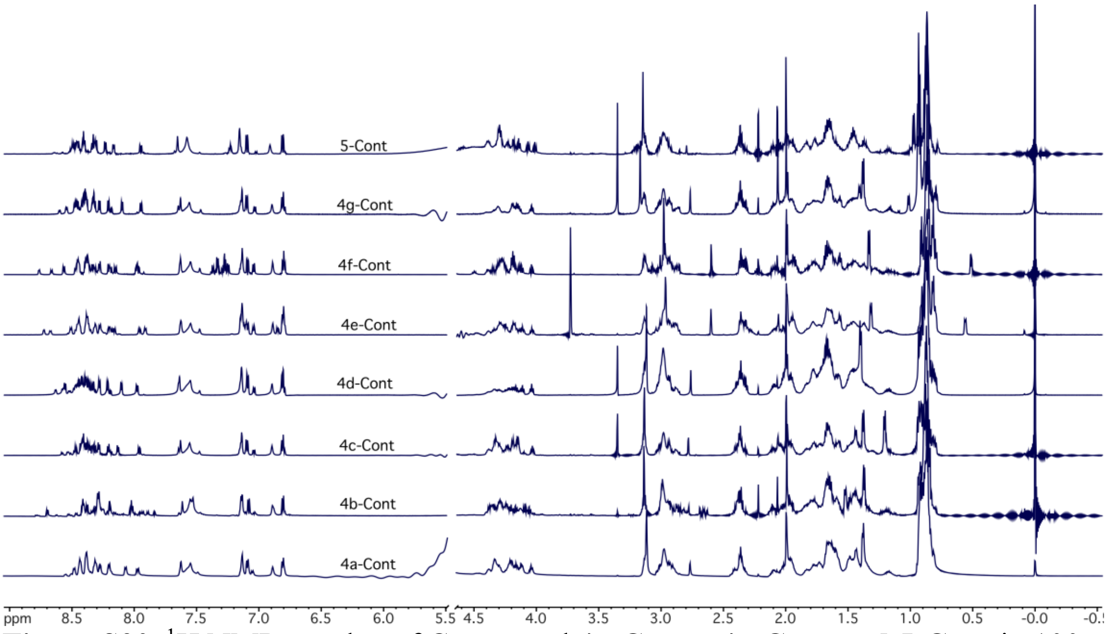

Figure S30. ^{1}H NMR overlay of Compound **4a-Cont – 4g-Cont and 5-Cont** in 100 mM Sodium Acetate buffer (pH 3.8) at 298K.

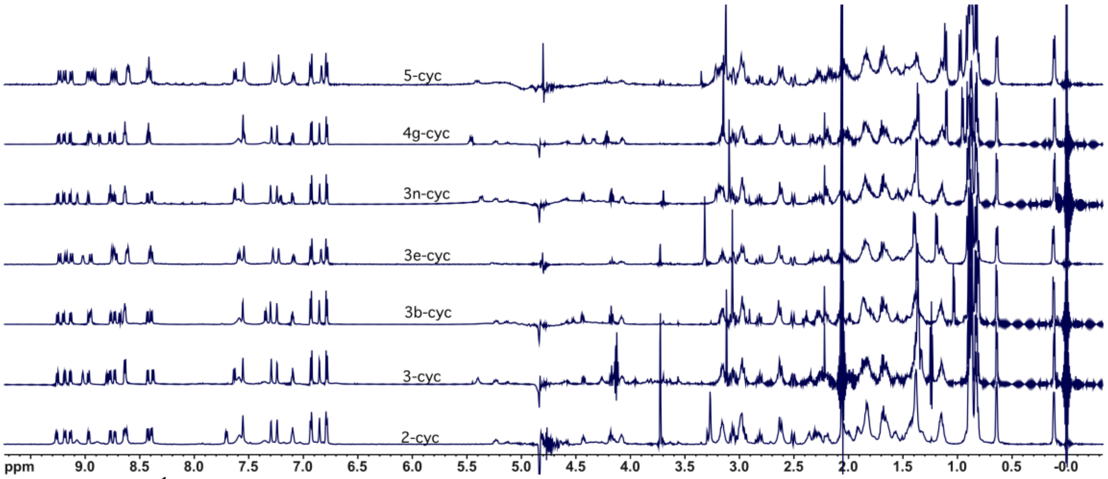

Figure S31.^{1}H NMR overlay of Compound **2-Cyc, 3-Cyc, 3b-Cyc, 3e-Cyc, 3n-Cyc, 4g-Cyc, 5-Cyc** in 100 mM Sodium Acetate buffer (pH 3.8) at 298K.

Table S7. Chemical Shifts of Compound **pG and PG-Cont**

**Residues**		**pG**	**PG-Cont**
**Arg1**	**HN**	8.13	8.19
	**HA**	4.37	4.18
**Tyr2**	**HN**	8.4	8.27
	**HA**	5.11	4.62
**Val3**	**HN**	8.87	7.95
	**HA**	4.39	4.02
**Glu4**	**HN**	8.54	8.38
	**HA**	5	4.3
**Val5**	**HN**	8.89	8.33
	**HA**	4.6	4.41
**D/L Pro6**	**HN**		
	**HA**	4.37	3.89
**Gly7**	**NMe**	8.57	8.5
	**HA**	4.00/3.78	3.95
**Lys8**	**HN**	7.92	8.38
	**HA**	4.56	4.31
**Lys9**	**HN**	8.42	8.11
	**HA**	4.65	4.33
**Ile10**	**HN**	9.03	8.28
	**HA**	4.49	4.13
**Leu11**	**HN**	8.58	8.4
	**HA**	4.11	4.38
**Gln12**	**HN**	8.65	
	**HA**	4.31	

Table S8. Chemical Shifts of Compound **4a-4g, 5a** and **4a-Cont – 4g-Cont** and **5-Cont.**

			**Chemical Shifts of Hairpin Peptides**								**Chemical Shifts of Control Peptides**							
**Residues**			**4a**	**4b**	**4c**	**4d**	**4e**	**4f**	**4g**	**5**	**4a-Cont**	**4b-Cont**	**4c-Cont**	**4d-Cont**	**4e-Cont**	**4f-Cont**	**4g-Cont**	**5-Cont**
**Arg1**	**HN**		8.14	8.15	8.16	8.15	8.14	8.15	8.11	8.13	8.19	8.18	8.2	8.2	8.2	8.19	8.2	8.2
	**HA**		4.34	4.37	4.34	4.36	4.36	4.38	4.37	4.38	4.16	4.2	4.2	4.2	4.21	4.2	4.22	4.19
	**HB**	**1**	1.69	1.68	1.68	1.69	1.68		1.66	1.73								
		**2**							1.73	1.65								
	**HG**	**1**	1.53	1.51	1.53	1.52	1.53			1.57								
		**2**								1.51								
	**HD**		3.18		3.18	3.17				3.16								
	**NAc**		1.97	2.06	1.97	1.97	1.96	1.96	1.96	1.96								
**Tyr2**	**HN**		8.38	8.4	8.41	8.4	8.42	8.43	8.39	8.43	8.26	8.28	8.28	8.27	8.27	8.27	8.28	8.28
	**HA**		4.99	5.03	5.01	5.04	5.04	5.07	5.08	5.12	4.63	4.65	4.63	4.64	4.63	4.63	4.63	4.61
	**HB**	**1**	2.82	2.83	2.83	2.79	2.77	2.79	2.81	2.79								
		**2**							2.74	2.71								
**Val3**	**HN**		8.64	8.66	8.68	8.75	8.77	8.79	8.8	8.89	7.97	8.02	7.95	7.97	7.95	7.95	7.93	7.92
	**HA**		4.29	4.32	4.31	4.34	4.34	4.35	4.37	4.41	4.04	4.06	4.03	4.04	4.03	4.03	4.06	4.02
	**HB**		1.99		1.98	1.99	1.99	1.99	1.99	2								
	**HG**	**1**	0.87		0.87	0.86	0.87	0.87	0.86	0.86								
		**2**																
**Glu4**	**HN**		8.55	8.58	8.59	8.6	8.61	8.62	8.59	8.62	8.38	8.39	8.4	8.38	8.38	8.38	8.39	8.38
	**HA**		4.79	4.78	4.76	4.84	4.84	4.86	4.89	4.95	4.33	4.31	4.33	4.32	4.32	4.31	4.34	4.31
	**HB**	**1**	2.04	2	2.04	2.05	2.06	2.07	2.04	2.03								

		**2**	1.91		1.89	1.89	1.9	1.92	1.87	1.85								
	**HG**	**1**	2.31	2.38	2.3	2.34	2.36	2.37	2.28	2.3								
		**2**				2.24		2.24		2.16								
**Val5**	**HN**		8.56	8.63	8.63	8.66	8.6	9.13	8.69	8.81	8.3	8.34	8.34	8.32	8.26	8.27	8.32	8.29
	**HA**		4.19	4.17	4.28	4.18	4.19	4.92	4.21	4.22	4.11	4.09	4.18	4.11	4.13	4.14	4.13	4.06
	**HB**		1.97		2	1.95	1.91	1.94	1.95	1.93								
	**HG**	**1**	0.92		0.93	0.91	0.88	0.9	0.91	0.91								
		**2**																
**Xaa6**	**HN**		8.77	8.37	8.88	8.89	9.08	9.13	8.83	8.95	8.48		8.36	8.55	8.5		8.4	8.46
	**HA**		4.71	4.38	4.61	4.69	4.85	4.92	4.37	4.4	4.81		4.83	4.77	5.01		4.63	4.59
	**HB**	**1**	1.67		4.09	1.8	3.09	3.18	2.01	2.02								
		**2**	1.58				2.93	3.03										
	**HG**	**1**			1.24				1.02	1.1								
		**2**								0.99								
	**HD**	**1**	0.97			1.55												
		**2**	0.93			1.38												
**Xaa7**	**NMe**		3.13	3.35	3.17	3.12	2.55	2.52	3.14	3.12	3.11	3.14	3.13	3.12	2.6	2.98	3.17	3.14
	**HA**		1.06	4.25	5.24	5.3	5.21	5.23	5.35	5.37		*	5.24	5.21	*		5.31	
	**HB**	**1**		1.4	1.37	1.36	1.07	1.74	1.38	2.11								
		**2**						1.43		1.76								
	**HG**	**1**								1.55								
		**2**								1.49								
	**HD**	**1**						0.92		3.21								
		**2**						0.81										

**Lys8**	**HN**		7.59	7.73	7.72	7.67	7.57	7.69	7.62	7.68	8.06	8.01	8.12	8.09	7.91	7.97	8.09	8.13
	**HA**		4.5	4.51	4.5	4.52	4.47	4.52	4.54	4.56	4.21	4.24	4.21	4.23	4.19	4.19	4.19	4.23
	**HB**	**1**	1.75	1.82	1.81	1.79	1.74		1.8	1.86								
		**2**						1.82		1.8								
	**HG**																	
	**HD**		1.35	1.41	1.42	1.41	1.35	1.42	1.41	1.4								
	**HE**									2.99								
**Lys9**	**HN**		8.37	8.4	8.42	8.42	8.36	8.4	8.4	8.45	8.38	8.28	8.4	8.37	8.38	8.38	8.38	8.44
	**HA**		4.67	4.65	4.64	4.65	4.66	4.67	4.69	4.7	4.33	4.32	4.32	4.33	4.32	4.33	4.33	4.3
	**HB**	**1**	1.6		1.63	1.61	1.59		1.64	1.63								
		**2**						1.63	1.568	1.52								
	**HG**		1.15		1.17	1.14	1.13	1.14	1.1	1.09								
	**HD**		1.34		1.38	1.36	1.34	1.37	1.37	1.32								
	**HE**																	
**Ile10**	**HN**		8.97	8.85	8.87	8.94	8.94	8.91	8.98	9.06	8.31	8.28	8.31	8.35	8.31	8.32	8.31	8.3
	**HA**		4.45	4.45	4.42	4.44	4.43	4.44	4.46	4.49	4.16	4.17	4.15	4.17	4.17	4.17	4.16	4.14
	**HB**		1.89	1.9	1.86	1.88	1.87	1.87	1.89	1.89								
	**HG**	**1**				1.17	1.16	1.17	1.17	1.17								
		**2**																
	**HD**		0.88	0.88	0.88	0.88	0.88	0.89	0.88	0.87								
**Leu11**	**HN**		8.58	8.55	8.57	8.57	8.55	8.55	8.55	8.59	8.43	8.42	8.42	8.44	8.44	8.44	8.46	8.42
	**HA**		4.12	4.17	4.16	4.13	4.12	4.15	4.11	4.09	4.38	4.36	4.38	4.37	4.38	4.38	4.39	4.38
	**HB**	**1**	1.59	1.59	1.59	1.58	1.58	1.58	1.58	1.59								
		**2**	1.4	1.44	1.44	1.42	1.4	1.41	1.39	1.38								

	**HG**																	
	**HD**	**1**	0.7	0.72	0.72	0.7	0.7	0.72	0.69	0.68								
		**2**	0.58	0.63	0.64	0.6	0.58	0.61	0.56	0.55								
**Gln12**	**HN**		8.65		8.61	8.63	8.64	8.62	8.64	8.68	*			8.39	*		*	
	**HA**		4.32		4.3	4.3	4.3	4.31	4.31	4.29	*			4.32	*		*	
	**HB**	**1**				2.05	2.04		2.04	2.03								
		**2**				1.87	1.86		1.87	1.85								
	**HG**	**1**	2.28			2.27	2.27		2.27	2.26								
		**2**						2.3										

Table S9. Chemical Shifts of fully folded peptides.

Residues			pG-cyc	2-cyc	3-cyc	3b-cyc	3e-cyc	3n-cyc	4g-cyc	5-cyc
Cys1	HN		8.44	8.42	8.42	8.42	8.4	8.42	8.42	8.4
	HA		5.22	5.23	5.23	5.23	5.24	5.23	5.24	5.23
	HB	1		3.05	3.04	3.05	3.05	3.05	3.05	3.03
		2		2.52	2.51	2.51	2.51	2.51	2.49	2.5
	NAc			2.06	2.05	2.06	2.06	2.06	2.06	2.06
Arg2	HN		8.74	8.73	8.72	8.72	8.71	8.73	8.73	8.72
	HA		4.60	4.6	4.59	4.59	4.59	4.59	4.59	4.57
	HB	1		1.83	1.6	1.6	1.84	1.6	1.6	
		2		1.65						
	HG	1		1.46	1.34	1.33		1.34	1.34	
		2								
	HD									
Tyr3	HN		8.78	8.77	8.76	8.76	8.74	8.77	8.77	8.75
	HA		5.17	5.13	5.13	5.14	5.13	5.13	5.13	5.13
	HB	1		2.82	2.81	2.82	2.82	2.81	2.81	2.81
		2		2.63	2.62	2.63	2.63	2.61	2.61	2.61
Val4	HN		9.27	9.26	9.25	9.25	9.23	9.25	9.24	9.23
	HA		4.46	4.43	4.43	4.43	4.43	4.43	4.43	4.43
	HB			2	1.99	2	1.99	1.98	1.98	1.99
	HG	1		0.86	0.85	0.84	0.85	0.85	0.85	0.85
		2								
Glu5	HN		8.57	8.62	8.63	8.63	8.61	8.63	8.63	8.6
	HA		5.05	4.93	4.93	4.99	4.95	4.93	4.91	4.94
	HB	1		2.04	2.04	2.08		2.04	2.04	
		2		1.92	1.88	1.87		1.84	1.86	
	HG	1		2.35	2.36	2.39		2.31	2.34	
		2			2.22	2.27		2.19	2.18	
Val6	HN		9.05	8.88	8.79	8.67	8.73	8.75	8.87	8.91
	HA		4.61	4.18	4.17	4.17	4.17	4.17	4.21	4.2
	HB			1.91	1.91	1.88	1.9	1.9	1.92	1.91
	HG	1		0.88	0.88	0.89	0.89	0.89	0.89	0.91
		2								
Xaa7	HN			9.07	9.02	8.95	9.01	9.08	8.95	8.98
	HA			4.71	4.74	4.73	4.84	4.78	4.33	4.36
	HB			1.39	1.36	1.36	1.4	1.37	2	2.01
	HG	1							1.1	1.11
		2							0.95	0.98
Xaa8	NMe		8.65	3.27	3.12	3.06	3.32	3.09	3.15	3.12
	HA		3.77/4.02		5.39	5.04	5.28	5.36	5.45	
	HB	1			1.35	2.21	4.55	2.09	1.36	
		2						1.75		
	HG	1				0.79	1.2	1.51		
		2								
	HD	1						3.19		

		2								
**Lys9**	**HN**		7.89	7.7	7.63	7.34	7.58	7.63	7.54	7.62
	**HA**		4.62	4.64	4.61	4.52	4.62	4.62	4.61	4.6
	**HB**	1		1.83	1.82	1.82	1.82	1.81	1.81	1.81
		2								
	**HG**			1.42	1.4	1.4	1.43	1.39	1.4	1.41
	**HD**									
	**HE**									
**Lys10**	**HN**		8.44	8.39	8.37	8.38	8.38	8.38	8.41	8.42
	**HA**		4.86	4.91	4.9	4.9	4.9	4.9	4.88	4.87
	**HB**	1		1.62	1.83	1.83	1.61	1.82	1.83	1.62
		2								
	**HG**			1.16	1.45	1.45	1.14	1.45	1.45	1.14
	**HD**			1.34	1.65	1.65	1.36	1.65	1.64	1.35
	**HE**									
**Ile11**	**HN**		9.22	9.18	9.18	9.19	9.17	9.19	9.19	9.18
	**HA**		4.59	4.57	4.57	4.58	4.57	4.58	4.56	4.56
	**HB**			1.87	1.86	1.87	1.86	1.87	1.86	1.86
	**HG**	1		1.16	1.15	1.16	1.16	1.16	1.16	1.16
		2								
	**HD**			0.89	0.88	0.88	0.88	0.88	0.88	0.87
**Leu12**	**HN**		8.65	8.64	8.63	8.63	8.6	8.63	8.63	8.61
	**HA**		4.09	4.08	4.07	4.09	4.09	4.07	4.07	4.07
	**HB**	1		1.68	1.67	1.67	1.66	1.67	1.67	1.67
		2		1.15	1.13	1.14	1.13	1.13	1.13	1.13
	**HG**									
	**HD**	1		0.64	0.63	0.64	0.63	0.64	0.63	0.63
		2		0.12	0.11	0.11	0.12	0.11	0.11	0.11
**Gln13**	**HN**		9.14	9.13	9.13	9.13	9.12	9.13	9.13	9.13
	**HA**		4.62	4.62	4.62	4.62	4.61	4.62	4.62	4.61
	**HB**	1		2.06	2.06	2.07		2.07	2.07	
		2		1.85	1.84	1.85		1.85	1.85	
	**HG**	1		2.23	2.23	2.23		2.23	2.22	
		2								
**Cys14**	**HN**		8.98	8.96	8.96	8.96	8.94	8.97	8.97	8.94
	**HA**		5.09	5.05	5.05	5.06	5.05	5.07	5.06	5.05
	**HB**	1		3.08	3.07	2.82	3.06	3.07	3.07	3
		2		2.95	2.95	2.63		2.95	2.96	

Table S10. Coupling constants of **3n, 4g and 5.**

	Arg1	Tyr2	Val3	Glu4	Val5	Xaa6	Lys8	Lys9	Ile10	Leu11	Gln12
**3n**	8.2	8.2	9.6	9			9.1			7	9.1
**4g**	8.2		9.9	8.3	9.7	5.5	9.7		9.5	5.5	8.4
**5**	8.3	8.3	9.7	8.3	9.7	5.5	8.2	8.1	8.3	6.8	8.3

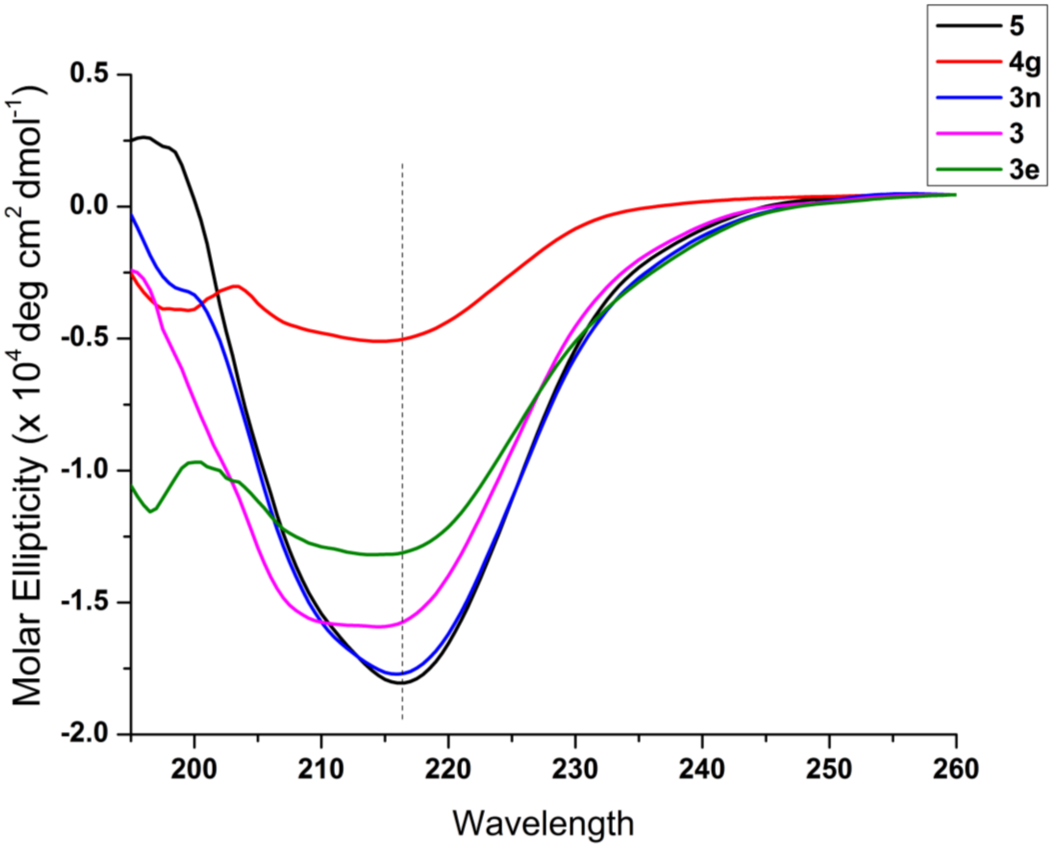

Figure S32. Far-UV CD spectra of compounds **3**, **3e**, **3n**, **4g** and **5** in 100 mM sodium acetate buffer (pH=3.8) at 25°C. The dotted lines indicate the red-shift in the minima of compounds **5** and **3n** in comparison to compounds **3**, **3e** and **4g**.

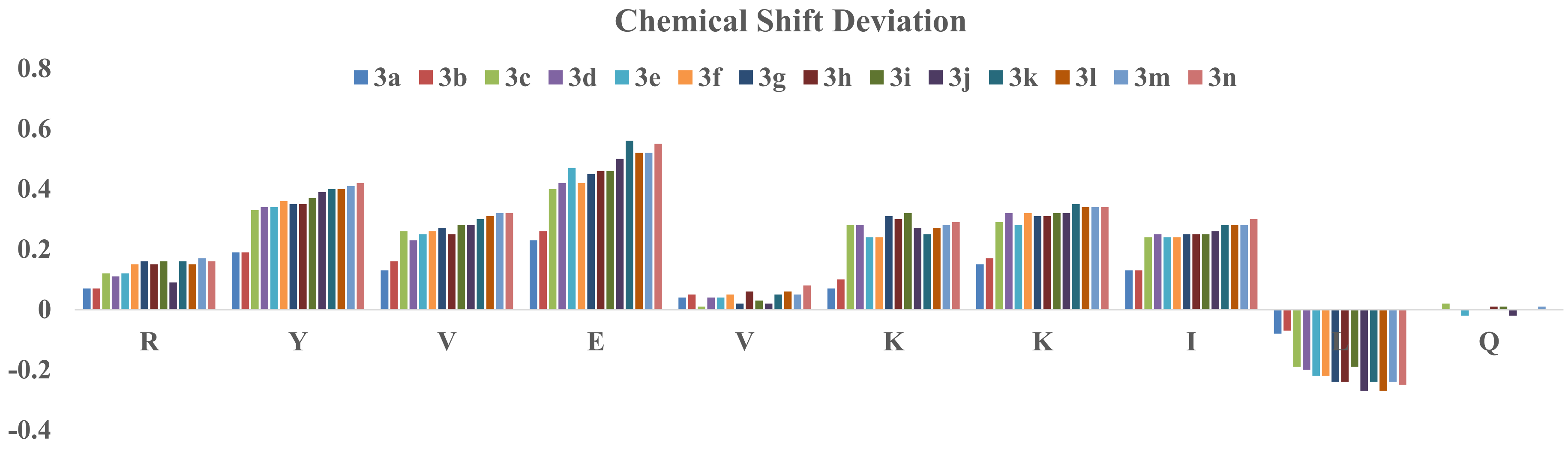

Figure S33. Chemical Shift deviation of hairpin peptides from **3a-3n**.

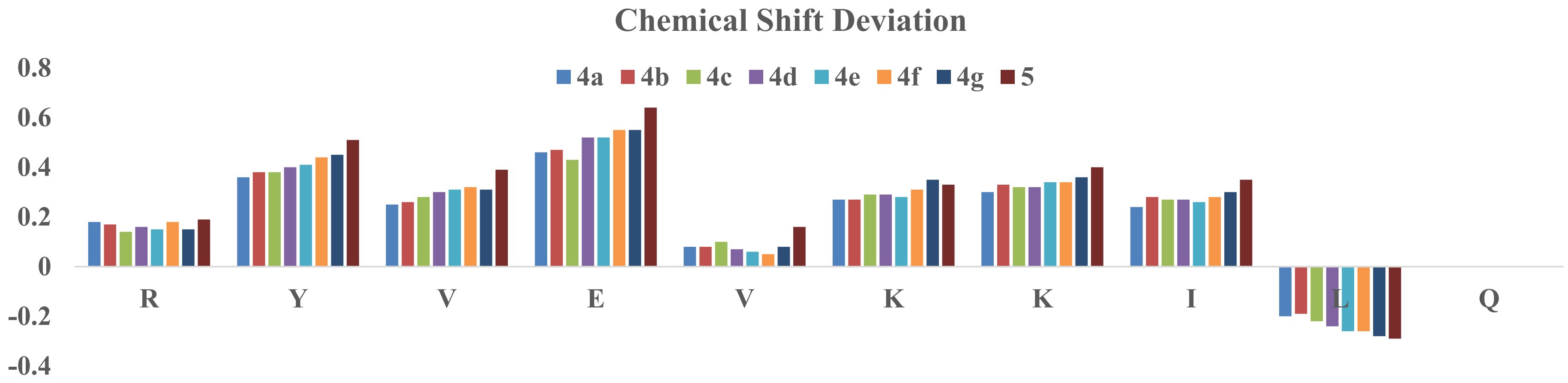

Figure S34. Chemical Shift deviation of hairpin peptides from **4a-4g** and **5**.

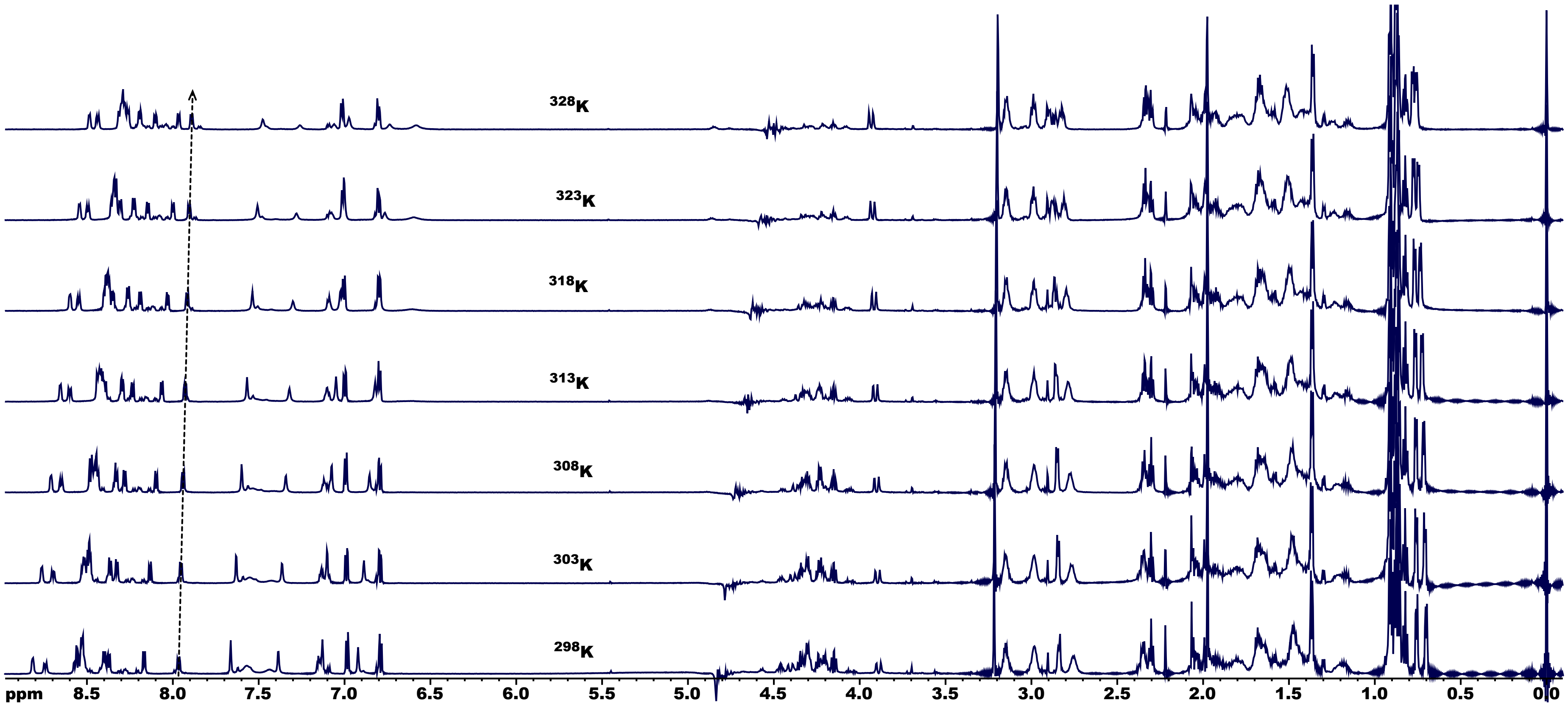

Figure S35. ^{1}H NMR overlay of Compound **2** acquired at different temperatures.

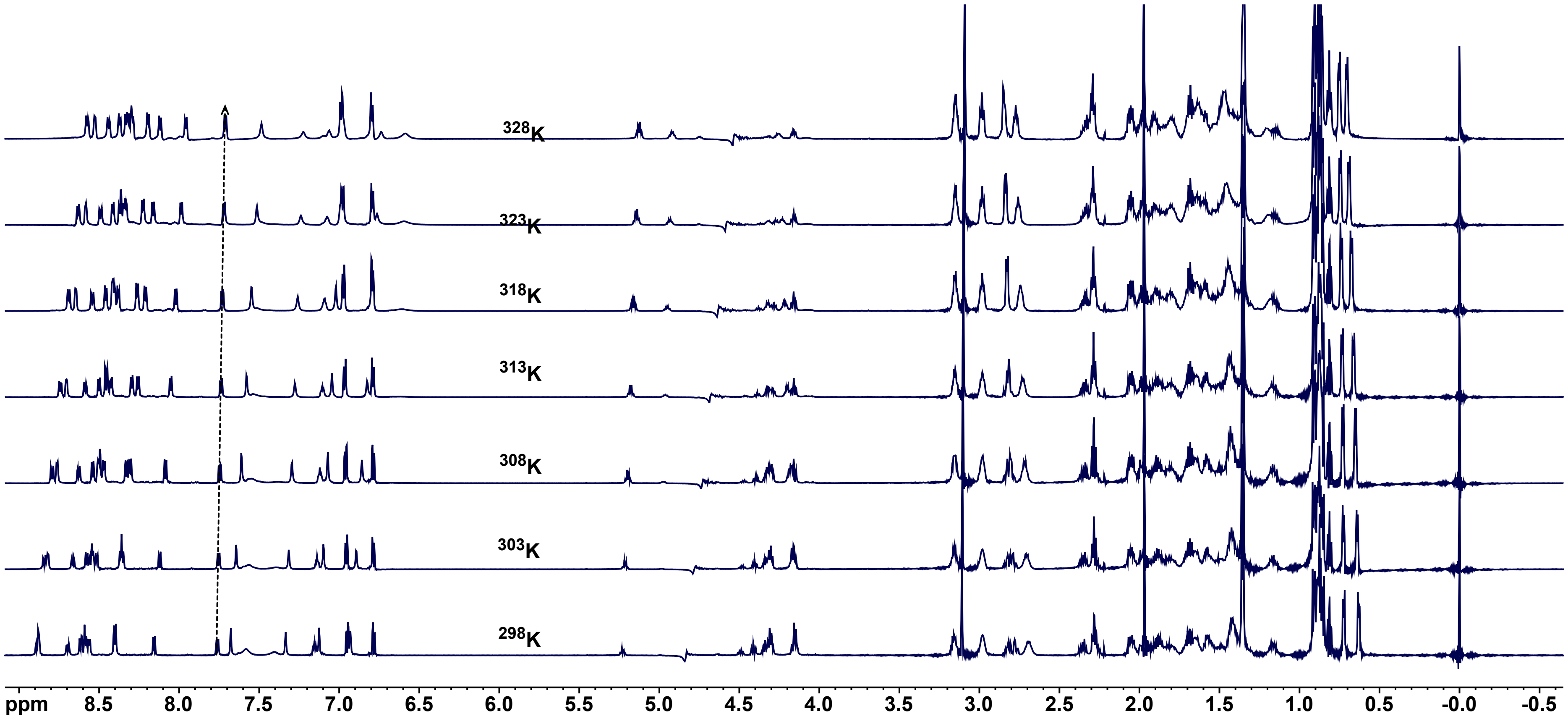

Figure S36. ^{1}H NMR overlay of Compound **3** acquired at different temperatures.

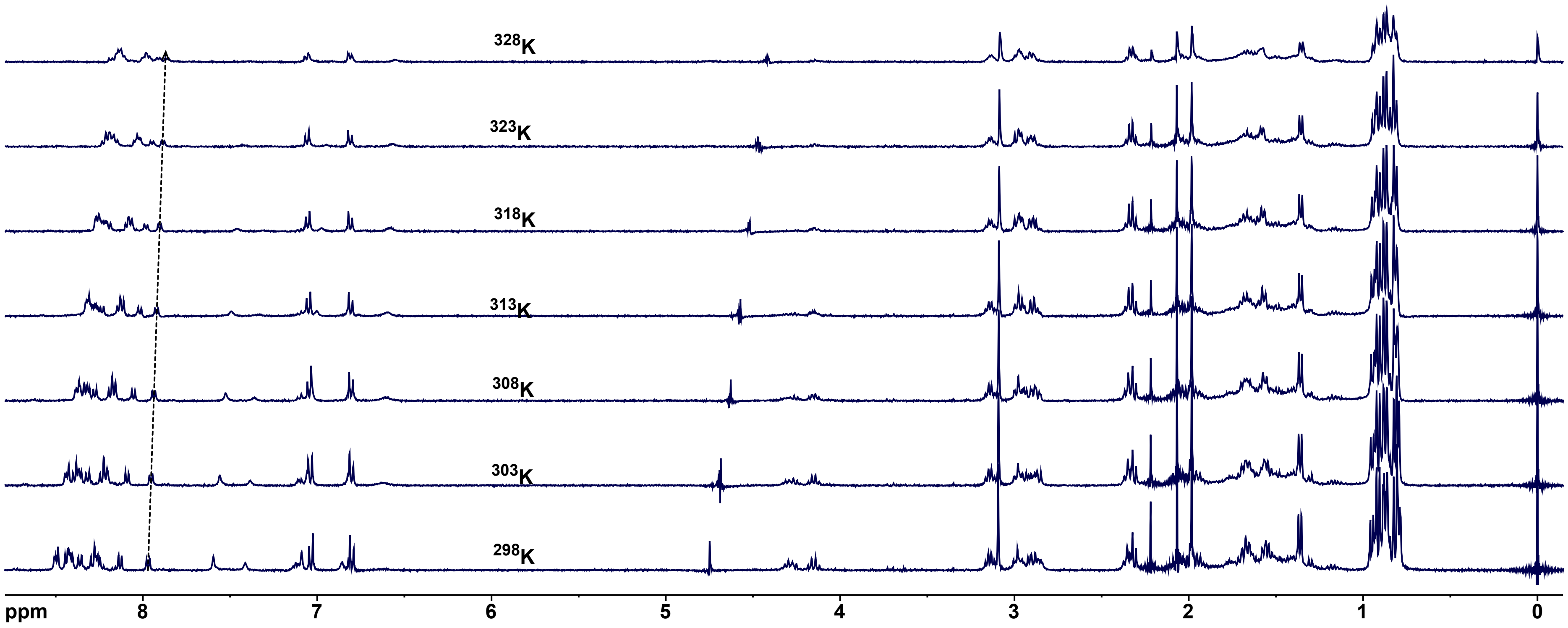

Figure S37. ^{1}H NMR overlay of Compound **3b** acquired at different temperatures.

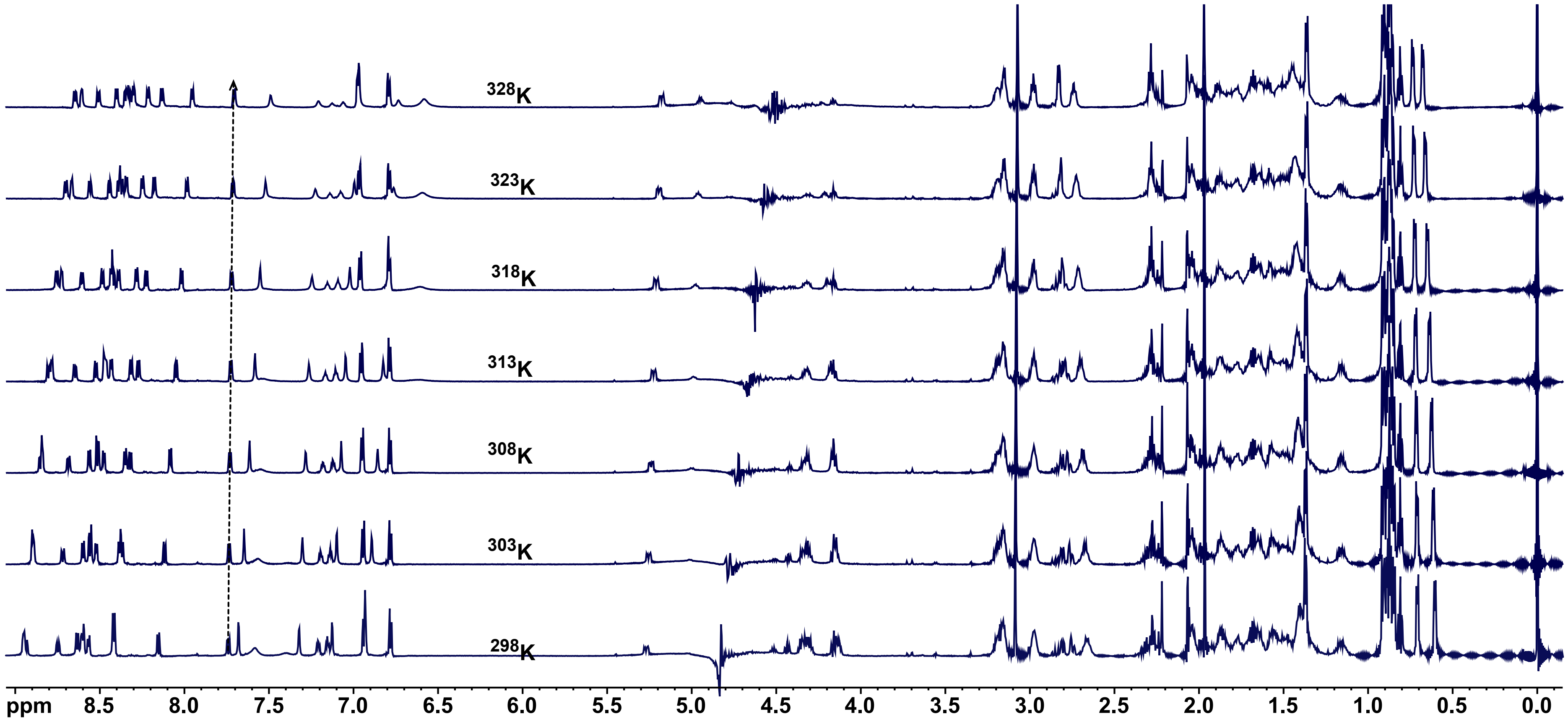

Figure S38. ^{1}H NMR overlay of Compound **3n** acquired at different temperatures.

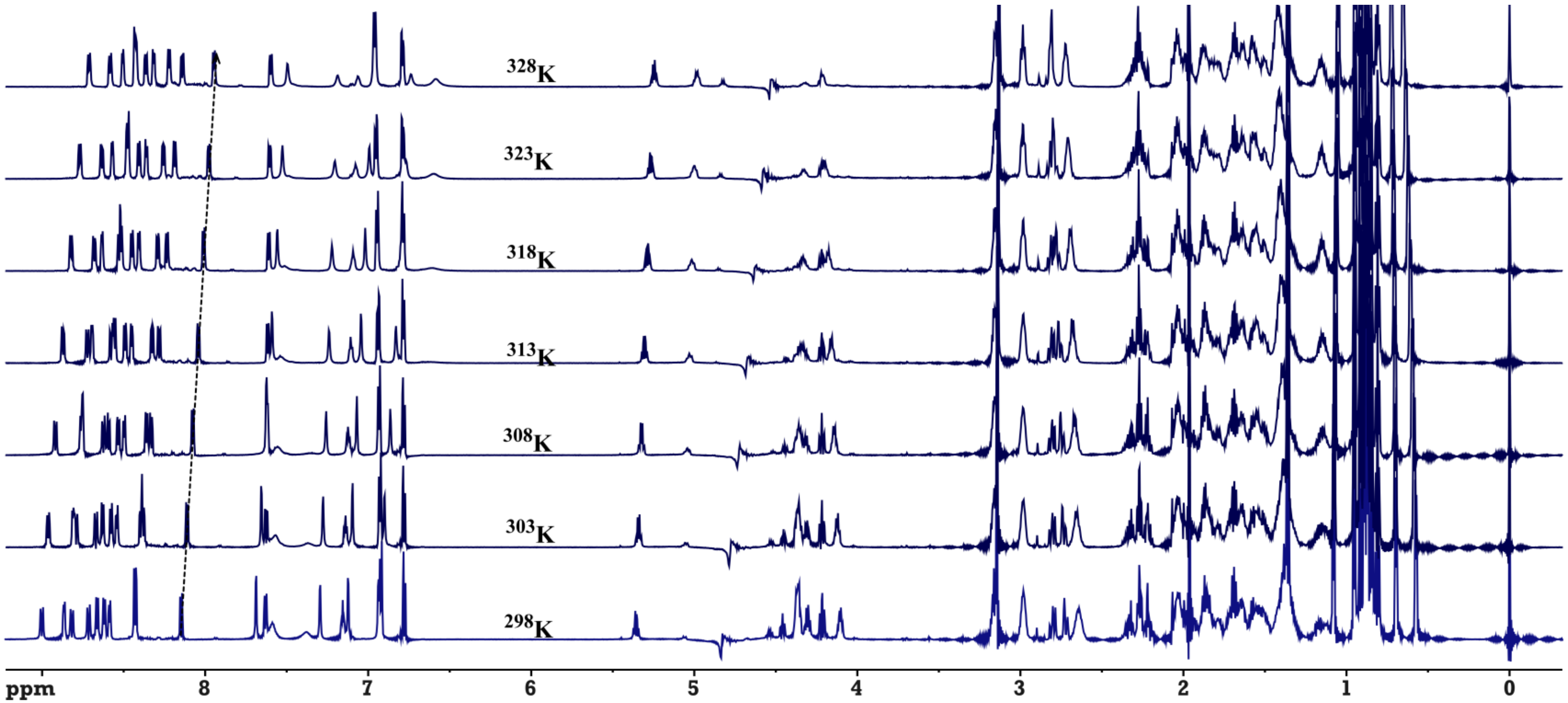

Figure S39. ^{1}H NMR overlay of Compound **4g** acquired at different temperatures.

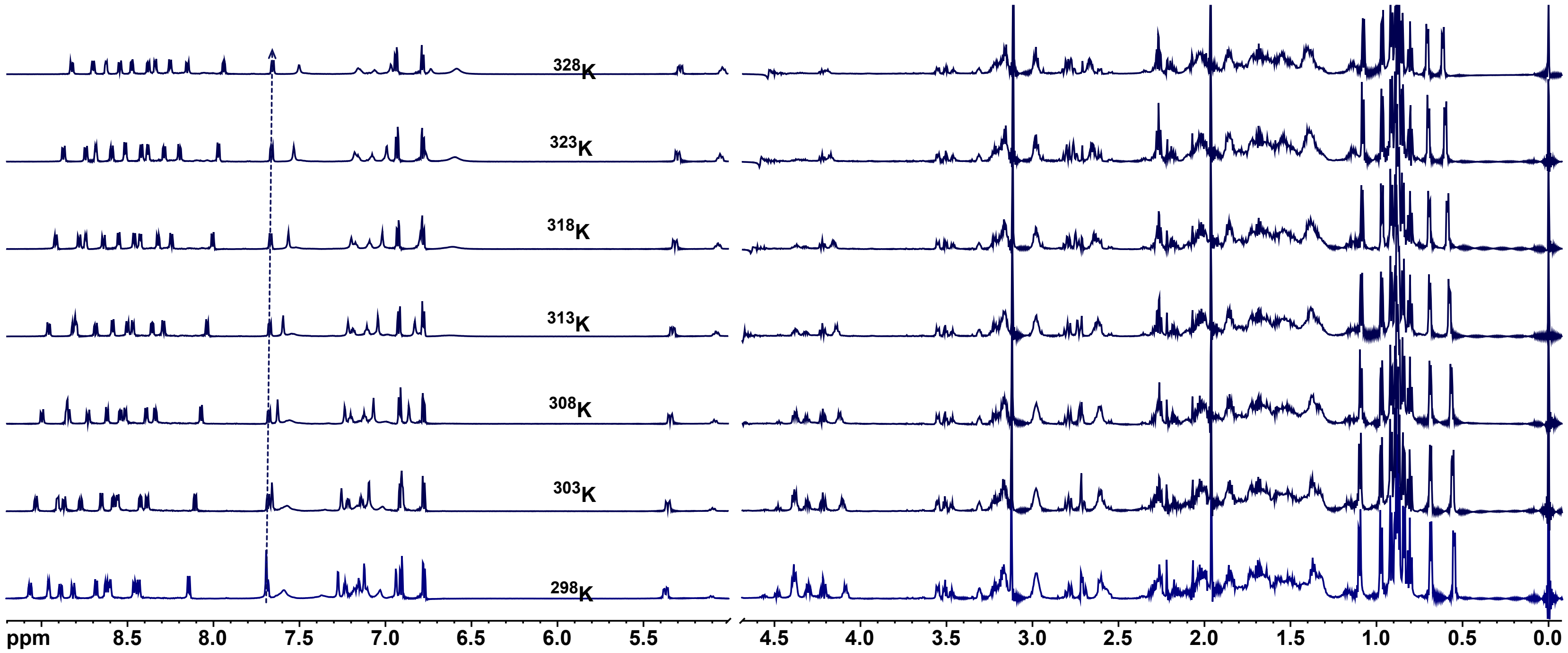

Figure S40. ^{1}H NMR overlay of Compound **5** acquired at different temperatures.

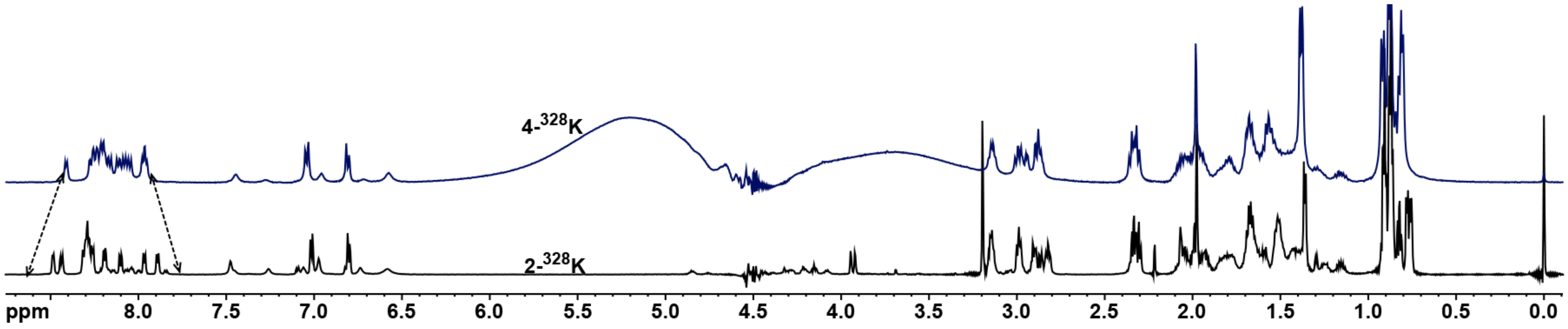

Figure S41. ^{1}H NMR overlay of Compound **2** and **4** at acquired at 328K.

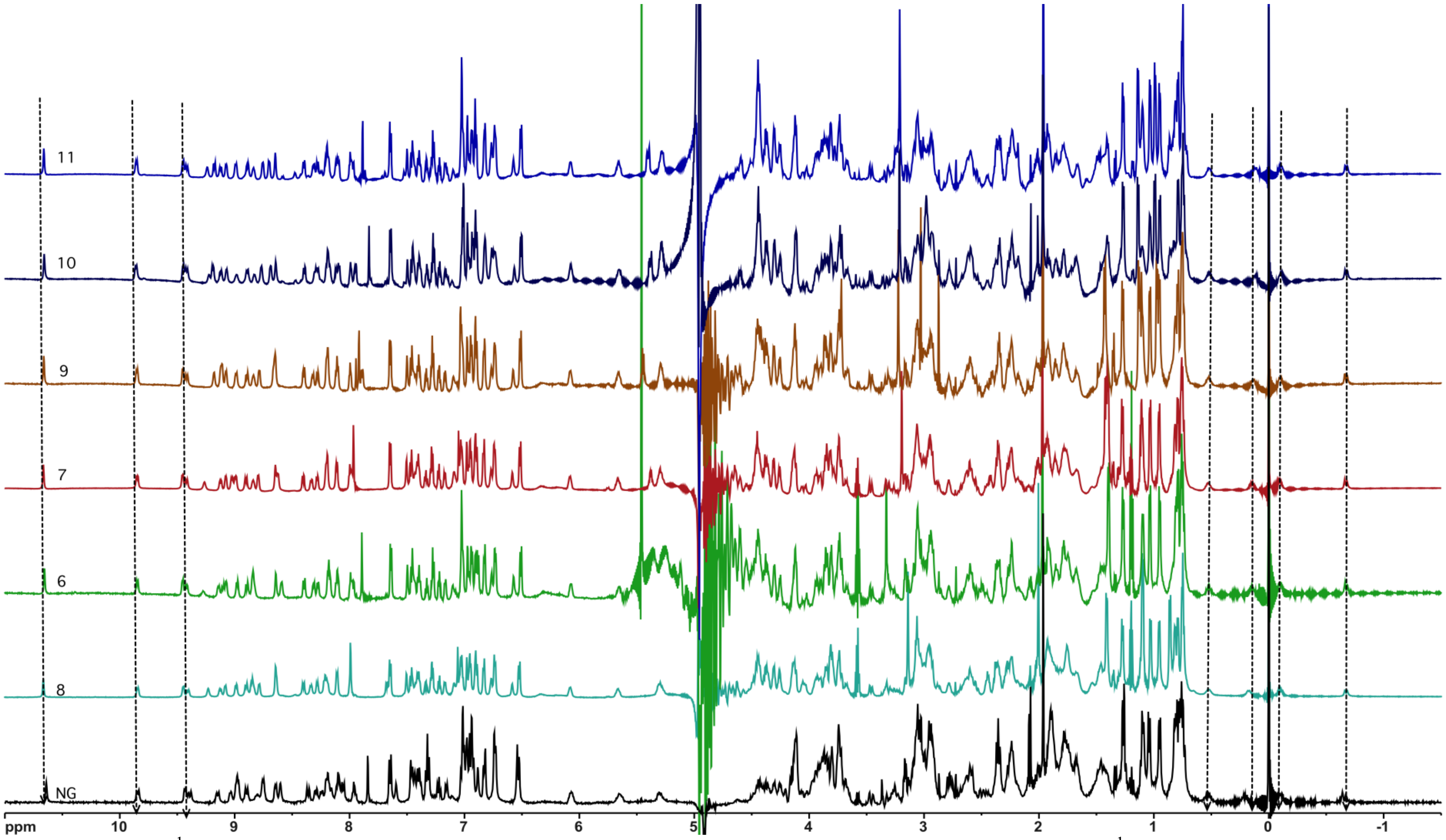

Figure S42. ^{1}H NMR overlay of Pin1-WW variants **NG**, **6-11** showing the characteristic upfield shifted ^{1}H resonances.

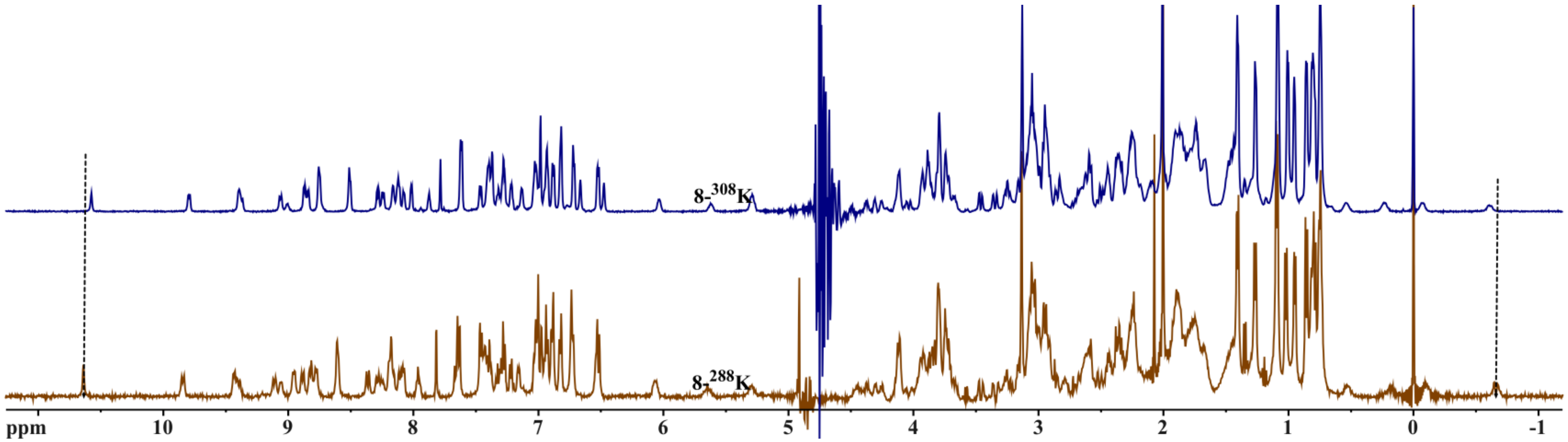

Figure S43. ^{1}H NMR overlay of Pin1 variant **8** acquired at 288 K and 308 K in 20 mM sodium phosphate buffer, pH=7.4.

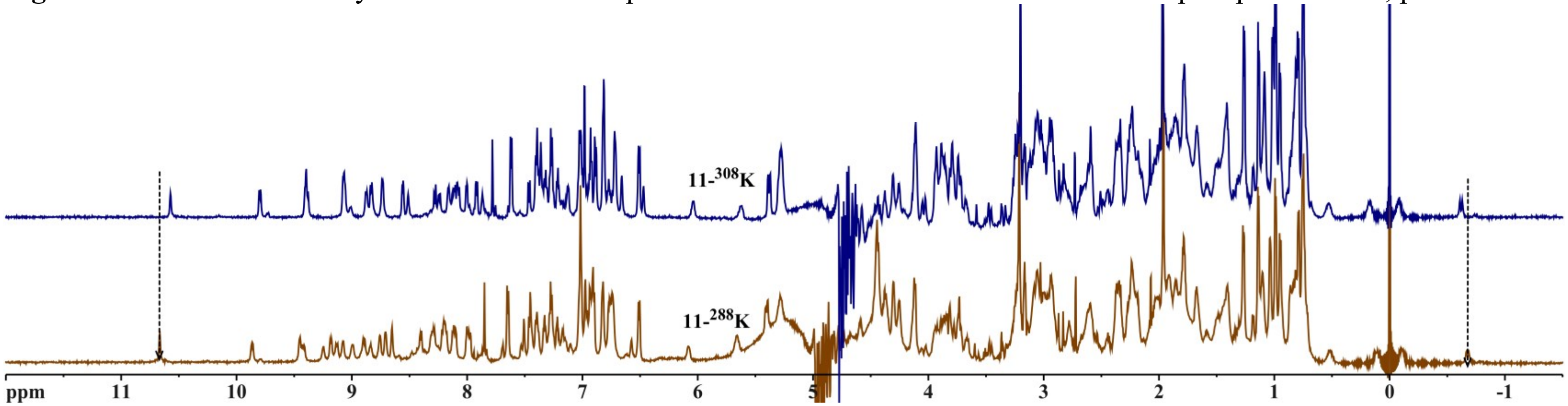

Figure S44. ^{1}H NMR overlay of Pin1 variant **11** acquired at 288 K and 308 K in 20 mM sodium phosphate buffer, pH=7.4.

Table S11. Chemical shifts of Pin1-WW analogs.

Residues		Compounds						
		**NG**	**6**	**7**	**8**	**9**	**10**	**11**
**Lys1**	HN							
	HA				4.02			
**Leu2**	HN				8.82	8.89		
	HA			4.64	4.64	4.63	4.64	4.62
**Pro3**	HN							
	HA	4.88	4.89	4.88	4.88	4.88	4.89	4.88
**Pro4**	HN							
	HA	4.38	4.39	4.38	4.38	4.39	4.39	4.38
**Gly5**	HN	8.81	8.83	8.83	8.84	8.83	8.82	8.83
	HA	4.04/3.35	3.35/4.05	4.04/3.31	4.06/3.33	3.33/4.05	4.04/3.34	4.03/3.32
**Trp6**	HN	7.4	7.4	7.39	7.39	7.38	7.37	7.38
	HA	5.28	5.3	5.27	5.29	5.29	5.29	5.28
**Glu7**	HN	9.84	9.86	9.83	9.86	9.85	9.85	9.85
	HA	4.85	4.87	4.85	4.86	4.86	4.86	4.86
**Lys8**	HN	8.97	9.02	8.98	8.98	8.98	8.98	8.98
	HA	4.40	4.42	4.42	4.43	4.42	4.41	4.44
**Arg9**	HN	8.89	8.94	8.9	8.9	8.9	8.88	8.89
	HA	4.49	4.51	4.46	4.48	4.47	4.46	4.46
**Met10**	HN	8.19	8.2	8.19	8.22	8.2	8.18	8.2
	HA	5.33	5.3	5.31	5.32	5.29	5.3	5.28
**Ser11**	HN	8.98	9.05	9.02	8.85	9.11	9.18	9.17
	HA	4.73	4.82	4.78	4.78	4.85	4.84	4.87
**Xaa12**	HN			9.26	9.23	9.18	9.22	9.23
	HA			4.96	4.97	4.42	4.96	4.43
**Yaa13**	NMe	8.75(HN)	3.34	3.19	3.12	3.23	3.22	3.2
	HA	4.19/3.71		5.38	5.02	5.44	5.38	5.4
**Arg14**	HN	8.06	7.92	7.98	7.68	7.89	7.94	7.97
	HA	4.78	4.72	4.72	4.75	4.72	4.71	4.72
**Val15**	HN	8.64	8.73	8.62	8.64	8.66	8.68	8.7
	HA	4.62	4.63	4.64	4.66	4.62	4.6	4.59
**Tyr16**	HN	8.75	8.63	8.79	8.79	8.78	8.76	8.75
	HA	4.80	4.81	4.79	4.79	4.79	4.8	4.79
**Tyr17**	HN	9.15	9.18	9.12	9.13	9.12	9.11	9.11
	HA	5.29	5.31	5.3	5.3	5.29	5.28	5.28
**Phe18**	HN	9.38	9.43	9.4	9.4	9.41	9.41	9.41
	HA	5.66	5.68	5.66	5.67	5.66	5.66	5.66
**Asn19**	HN	8.16	8.19	8.22	8.2	8.19	8.17	8.18
	HA	4.45	4.47	4.44	4.46	4.46	4.47	4.46
**His20**	HN	8.19	8.21	8.19	8.2	8.19	8.18	8.18

	HA	4.11	4.14	4.15	4.14	4.13	4.13	4.13
**Ile21**	HN	8.34	8.33	8.34	8.32	8.31	8.29	8.32
	HA	3.86	3.87	3.85	3.86	3.85	3.84	3.85
**Thr22**	HN	7.42	7.43	7.4	7.43	7.43	7.43	7.43
	HA	4.12	4.13	4.12	4.11	4.11	4.11	4.11
**Asn23**	HN	8.10	8.13	8.1	8.12	8.11	8.11	8.11
	HA	4.14	4.15	4.16	4.15	4.14	4.14	4.14
**Ala24**	HN	7.16	7.17	7.16	7.17	7.16	7.16	7.16
	HA	4.44	4.46	4.46	4.45	4.45	4.45	4.45
**Ser25**	HN	8.36	8.4	8.4	8.4	8.39	8.38	8.39
	HA	6.09	6.09	6.08	6.08	6.09	6.07	6.08
**Gln26**	HN	9.42	9.43	9.44	9.43	9.44	9.44	9.43
	HA	4.95	4.94	4.92	4.93	4.92	4.93	4.93
**Phe27**	HN	9.03	9.07	9.07	9.08	9.07	9.19	9.07
	HA	4.85	4.84	4.84	4.85	4.84	4.85	4.83
**Glu28**	HN	8.09	8.14	8.1	8.1	8.1	8.09	8.09
	HA	4.36	4.36	4.36	4.36	4.36	4.35	4.36
**Arg29**	HN	8.60	8.63	8.64	8.65	8.63	8.63	8.64
	HA	2.78	2.8	2.78	2.79	2.79	2.79	2.78
**Pro30**	HN							
	HA	3.91	3.92	3.91	3.92	3.91	3.9	3.89
**Ser31**	HN	8.24	8.28	8.28	8.29	8.28	8.27	8.28
	HA	4.30	4.32	4.3	4.31	4.31	4.3	4.31
**Gly32**	HN	7.96	7.99	7.99	8	7.99	7.99	7.98
	HA	3.77	3.82/3.74	3.73/3.81	3.74/3.82	3.81/3.73	3.72/3.80	3.80/3.73

Note: The chemical shifts of apo-Pin1 WW domain with –RSSG- turn motif has been obtained from BMRB with accession id: BMR5248

Table S12. Characteristic NOEs of **2, 3, 3a-3n**.

	CHARACTERISTIC NOEs																				
	TURN NOEs								STRAND NOEs												
	Val5HA-xaa6HN	xaa6HN-HA	xaa6HN-HB	xaa6HA-Yaa7NMe	xaa6HA-Lys8HN	Yaa7NMeLys8HN	Yaa7NMe-Yaa7HB	Yaa7HA-Lys8HN	Arg1NAc-HN	Arg1HN-Gln12HN	Tyr2HA-Leu11HA	Tyr2HA-Val3HN	Val3HN-Ile10HN	Leu11HA-Gln12HN	Ile10HN-Lys9HA	Glu4HA-Val5HN	Val5HN-Lys8HN	Tyr2HA-Gln12HN	Val5HN-Lys9HA	Leu11HA-Tyr2HD/Tyr2HE	Ile10HN-Glu4HA
**2**	✓	✓	✓	✓		✓	✓	✓	✓			✓		✓	✓	✓	✓			✓	
**3**	✓	✓	✓	✓		✓	✓	✓	✓	✓	✓	✓		✓	✓	✓	✓	✓			✓
**3a**	✓	✓	✓	✓		✓	✓	✓	✓		✓	✓			✓	✓	✓			✓	
**3b**	✓	✓	✓	✓		✓	✓	✓	✓		✓	✓		✓	✓	✓		✓		✓	
**3c**	✓	✓	✓	✓		✓	✓	✓	✓		✓	✓		✓	✓	✓		✓		✓	✓
**3d**	✓	✓	✓	✓	✓	✓	✓	✓	✓		✓	✓	✓	✓	✓	✓				✓	
**3e**	✓	✓	✓	✓	✓	✓	✓	✓	✓		✓	✓		✓	✓	✓	✓			✓	
**3f**	✓	✓	✓	✓	✓	✓	✓	✓	✓		✓	✓		✓	✓	✓	✓	✓		✓	✓
**3g**	✓	✓	✓	✓		✓		✓	✓		✓	✓		✓	✓	✓		✓	✓	✓	✓
**3h**	✓	✓	✓	✓	✓	✓	✓	✓	✓		✓	✓		✓	✓	✓	✓	✓		✓	
**3i**	✓	✓	✓	✓		✓	✓	✓	✓		✓	✓		✓	✓	✓				✓	✓
**3j**	✓	✓	✓	✓		✓	✓	✓	✓		✓	✓		✓	✓	✓	✓	✓		✓	✓
**3k**	✓		✓	✓		✓	✓	✓	✓		✓	✓		✓	✓	✓	✓	✓		✓	✓

**3l**	✓	✓	✓	✓		✓	✓	✓	✓		✓	✓		✓	✓	✓	✓	✓		✓	✓
**3m**	✓	✓	✓	✓	✓	✓	✓	✓	✓		✓	✓	✓	✓	✓	✓	✓	✓	✓	✓	✓
**3n**	✓	✓	✓	✓	✓	✓	✓	✓	✓		✓	✓		✓	✓	✓	✓	✓		✓	✓

Table S13. Characteristic NOEs of **4a-4g** and **5**.

	**CHARACTERISTIC NOEs**																				
	**TURN NOEs**								**STRAND NOEs**												
	Val5HA-xaa6HN	xaa6HN-HA	xaa6HN-HB	xaa6HA-Yaa7NMe	xaa6HA-Lys8HN	Yaa7NMe-Lys8HN	Yaa7NMe-Yaa7HB	Yaa7HA-Lys8HN	Arg1NAc-HN	Arg1HN-Gln12HN	Tyr2HA-Leu11HA	Tyr2HA-Val3HN	Val3HN-Ile10HN	Leu11HA-Gln12HN	Ile10HN-Lys9HA	Glu4HA-Val5HN	Val5HN-Lys8HN	Tyr2HA-Gln12HN	Val5HN-Lys9HA	Leu11HA-Tyr2HD/HE	Ile10HN-Glu4HA
**4a**	✓	✓	✓	✓	✓	✓	✓	✓	✓		✓	✓		✓	✓	✓	✓	✓		✓	✓
**4b**	✓	✓	✓			✓	✓		✓		✓	✓		✓	✓	✓					
**4c**	✓	✓		✓		✓	✓	✓	✓		✓	✓		✓	✓	✓		✓		✓	
**4d**	✓	✓	✓	✓		✓	✓	✓	✓		✓	✓	✓	✓	✓	✓	✓	✓		✓	✓
**4e**	✓	✓	✓	✓		✓	✓	✓	✓		✓	✓		✓	✓	✓	✓	✓		✓	
**4f**	✓	✓	✓	✓		✓	✓	✓	✓		✓	✓		✓	✓	✓	✓	✓			
**4g**	✓	✓	✓	✓		✓	✓	✓	✓	✓	✓	✓		✓	✓	✓	✓	✓		✓	✓
**5**	✓	✓	✓	✓	✓	✓	✓	✓	✓	✓	✓	✓		✓	✓	✓	✓	✓		✓	

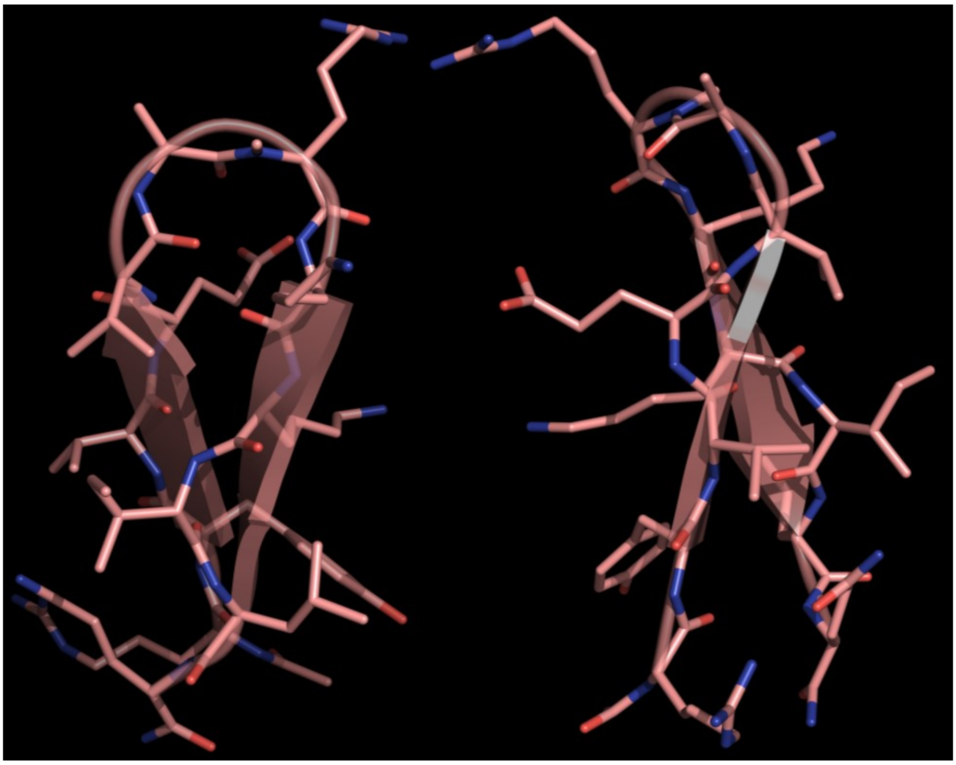

Figure S45. Front view and side view of average solution structure of compound **3n**.

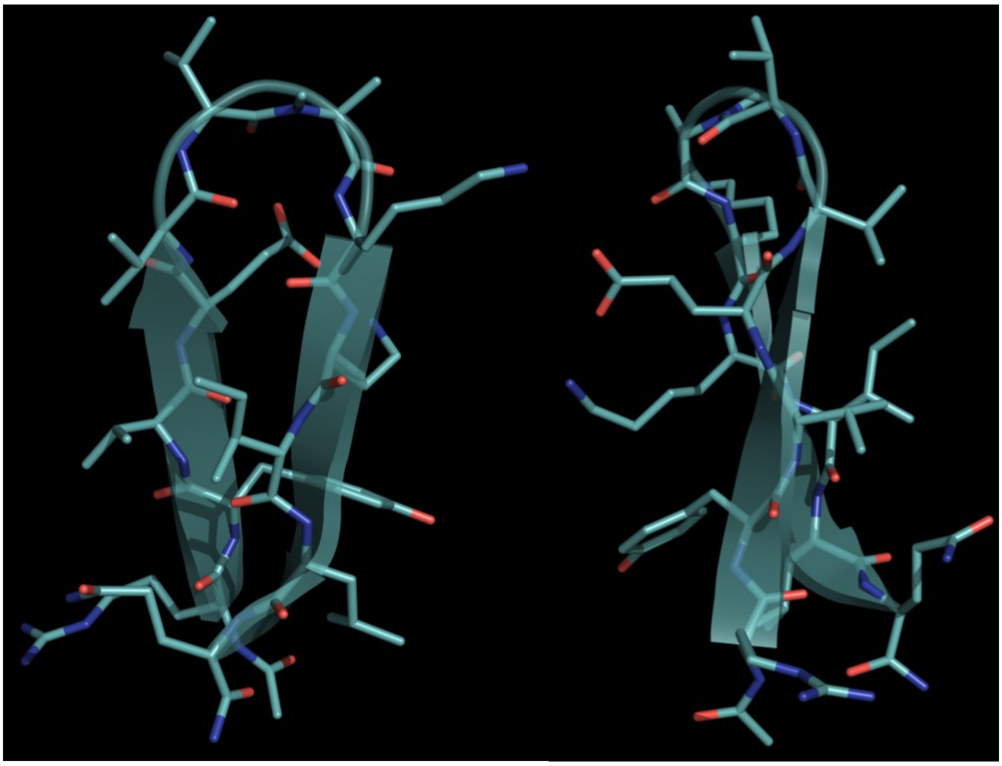

Figure S46. Front view and side view of average solution structure of compound **4g**.

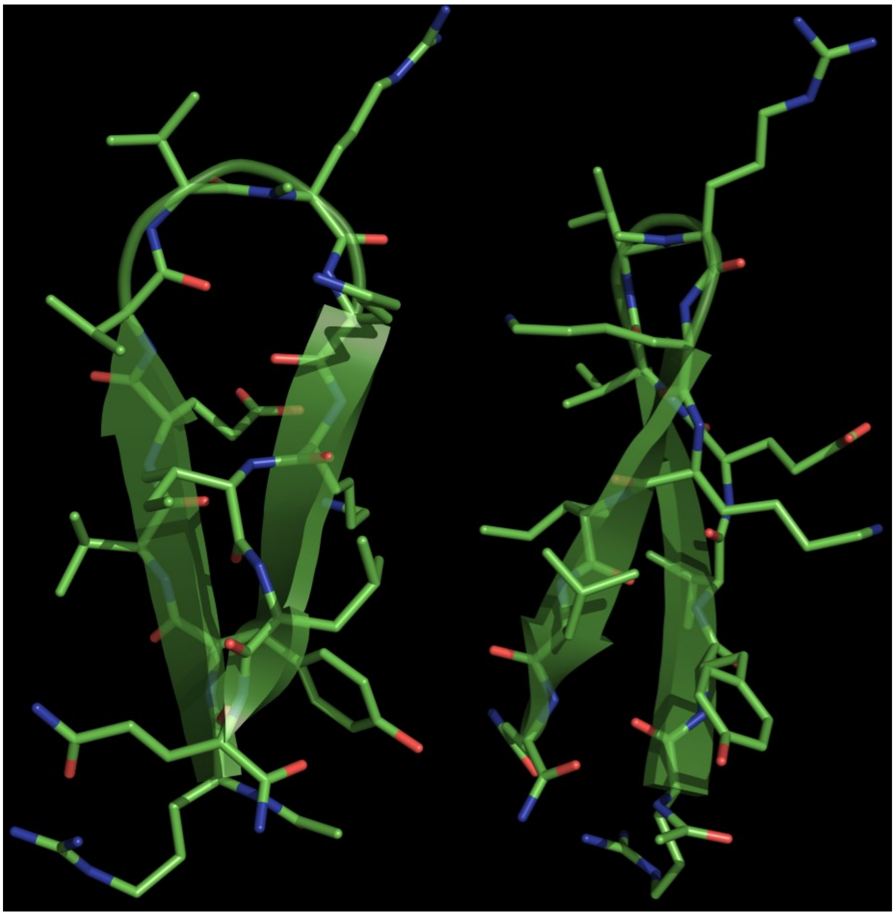

Figure S47. Front view and side view of average solution structure of compound **5**.

Table S14. Distance list of NOEs for compound **3n,** considered for structure calculation and violation observed.

NOEs	Min. Distance	Max. Distance	Observed Distance	Violations
Arg1HN-HA	2.01	2.45	2.91	0.46
Tyr2HN-Arg1HA	1.82	2.23	2.32	0.10
Arg1HA-HB1	1.72	2.10	2.79	0.70
Arg1HN-Gln12HN	2.76	3.38	3.61	0.23
Arg1HN-NAc	1.35	2.54	2.83	0.29
Tyr2HA-HB2	1.64	2.00	2.41	0.41
Tyr2HA-HD1	1.73	2.12	2.38	0.26
Tyr2HA-HN	1.96	2.40	2.93	0.54
Tyr2HA-Val3HN	1.61	1.97	2.28	0.30
Tyr2HA-Leu11HA	1.74	2.13	2.54	0.42
Tyr2HA-Gln12HN	2.06	2.52	2.89	0.37
Tyr2HA-HB1	1.75	2.14	3.03	0.89
Tyr2HB1-HB2	1.70	2.08	1.75	0.00
Tyr2HD1-HB2	1.80	2.20	2.24	0.04
Tyr2HD1-Lys9HB1	2.51	3.06	3.47	0.41
Tyr2HD1-Lys9HB2	2.23	2.72	2.25	0.00
Val3HN-HA	1.96	2.40	2.91	0.51
Glu4HN-Val3HA	1.75	2.13	2.30	0.17
Val3HB-HA	1.92	2.34	2.51	0.17
Val3HA-HG1	1.54	2.77	2.88	0.11

Glu4HA-Val5HN	1.46	1.78	2.03	0.25
Ala6HN-Val5HA	1.59	1.94	2.18	0.23
Val5HA-HB	1.87	2.29	2.52	0.23
Val5HA-HG1	1.28	2.46	2.85	0.39
Ala6HN-HB	1.73	2.12	2.71	0.59
Arg7HA-NMe	1.57	2.81	3.75	0.94
Arg7HA-HB1	1.99	2.43	2.98	0.55
Arg7HA-HB2	2.21	2.71	2.39	0.00
Arg7HA-HG1	1.92	2.35	2.50	0.15
Lys8HN-Arg7NMe	1.41	2.61	2.82	0.21
Lys8HN-HA	1.81	2.21	2.90	0.69
Lys9HN-Lys8HA	2.12	2.59	2.40	0.00
Lys8HA-HB1	1.68	2.06	2.40	0.34
Lys9HN-Lys8HB1	2.16	2.64	2.36	0.00
Lys8HN-Val5HN	2.47	3.02	3.34	0.32
Lys9HA-HB1	1.86	2.27	3.01	0.73
Lys9HA-HB2	1.86	2.28	2.44	0.17
Lys9HA-HG1	2.04	2.49	2.74	0.25
Lys9HA-HN	2.10	2.56	2.90	0.34
Lys9HA-Ile10HN	1.51	1.85	2.11	0.26
Lys9HB2-Ile10HN	2.19	2.67	2.77	0.09
Tyr2HD1-Lys9HG1	2.24	2.74	3.27	0.53
Ile10HN-HA	2.00	2.44	2.93	0.49
Leu11HN-Ile10HA	1.94	2.38	2.43	0.05
Ile10HA-HB	2.30	2.82	3.03	0.21
Ile10HN-HB	1.91	2.33	2.46	0.13
Ile10HA-HD	1.98	3.30	3.02	0.00
Leu11HA-Tyr2HE1	2.39	2.92	2.83	0.00
Leu11HA-HN	1.85	2.26	2.96	0.70
Gln12HN-Leu11HA	1.52	1.86	2.16	0.30
Leu11HA-HB1	1.97	2.41	2.93	0.52
Leu11HB1-HB2	1.57	1.91	1.69	0.00
Tyr2HE1-Leu11HB2	2.01	2.46	2.08	0.00
Leu11HA-HB2	1.83	2.24	2.25	0.01
Leu11HA-HD1	1.54	2.77	4.60	1.84
Tyr2HD1-Leu11HD2	2.67	4.15	3.05	0.00
Tyr2HE1-Leu11HD2	2.59	4.05	2.93	0.00
Leu11HA-HD2	1.63	2.89	3.32	0.44
Gln12HA-HB1	1.86	2.27	3.00	0.73
Gln12HA-HG1	1.88	2.30	2.52	0.22
Val3HN-Tyr2HB1	2.39	2.93	3.44	0.51

Table S15. Distance list of NOEs for compound **4g,** considered for structure calculation and violation observed.

NOEs	Min. Distance	Max. Distance	Observed Distance	Violations
Arg1HA-HB2	1.79	2.18	3.02	0.84
Arg1HA-HN	2.03	2.48	2.90	0.42
Tyr2HN-Arg1HA	1.49	1.83	2.19	0.37
Arg1HB1-HN	2.28	2.78	3.12	0.33

Arg1HA-HB1	1.88	2.30	2.49	0.20
Arg1HN-HB2	1.90	2.32	2.62	0.29
Arg1HN-Gln12HN	2.68	3.27	3.27	0.00
Arg1HN-NAc	1.36	2.56	2.75	0.19
Tyr2HA-HB1	1.89	2.31	3.00	0.69
Tyr2HA-HB2	1.88	2.29	2.39	0.10
Tyr2HA-HN	2.01	2.45	2.82	0.37
Tyr2HA-Val3HN	1.55	1.89	2.22	0.33
Tyr2HA-Leu11HA	1.76	2.15	2.25	0.09
Tyr2HA-Gln12HN	2.21	2.70	2.74	0.03
Tyr2HN-HB1	2.00	2.45	2.47	0.03
Val3HN-Tyr2HB1	2.21	2.70	3.40	0.70
Tyr2HB1-HB2	1.62	1.98	1.73	0.00
Tyr2HD1-HB2	1.80	2.20	2.38	0.17
Tyr2HE1-Leu11HD2	2.56	4.02	3.67	0.00
Tyr2HN-HB2	2.13	2.61	3.49	0.89
Glu4HN-Val3HA	1.45	1.77	2.29	0.52
Val3HA-HB	1.94	2.38	2.50	0.12
Val3HG1-HA	1.48	2.70	2.89	0.20
Val3HN-Tyr2HB2	2.54	3.10	2.35	-0.19
Glu4HA-HN	1.69	2.07	2.91	0.84
Glu4HA-Val5HN	1.51	1.85	2.11	0.27
Glu4HN-HB1	1.76	2.15	2.81	0.66
Glu4HN-HB2	1.72	2.10	2.56	0.46
Val5HA-HN	1.92	2.34	2.93	0.58
Val5HA-Val6HN	1.75	2.13	2.17	0.03
Val5HB-HA	2.03	2.49	3.00	0.52
Val5HG1-HA	1.49	2.70	3.01	0.31
Val5HN-HB	1.74	2.13	2.58	0.46
Val6HA-HG1	1.44	2.64	2.89	0.25
Val6HN-HA	2.27	2.77	2.81	0.04
Val6HN-HB	1.70	2.08	2.34	0.26
Lys8HN-Ala7HA	1.94	2.37	3.01	0.65
Ala7HA-HB	1.43	2.63	2.47	0.00
Ala7NMe-HB	0.87	2.85	3.29	0.44
Val6HA-Ala7NMe	1.08	2.20	2.52	0.32
Ala7HA-NMe	1.54	2.78	3.72	0.94
Lys8HN-Ala7NMe	1.42	2.62	2.83	0.21
Lys8HA-HD1	1.84	2.25	2.44	0.19
Lys8HA-HN	1.90	2.32	2.98	0.66
Lys8HA-Lys9HN	1.42	1.74	2.07	0.34
Lys8HN-HB1	1.83	2.24	2.52	0.28
Val5HN-Lys8HN	2.66	3.25	3.46	0.22
Lys9HA-Ile10HN	1.56	1.90	2.21	0.31
Lys9HN-HB1	1.87	2.29	2.16	0.00
Lys9HB2-Tyr2HD1	2.01	2.46	3.10	0.64
Lys9HG1-Tyr2HD1	2.45	3.00	3.06	0.06
Lys9HN-HB2	1.97	2.41	3.15	0.74
Ile10HA-HN	2.14	2.61	2.92	0.31
Leu11HN-Ile10HA	1.49	1.82	2.12	0.31

Ile10HB-HA	2.55	3.11	3.04	0.00
Ile10HN-HB	2.01	2.45	2.79	0.33
Tyr2HD1-Leu11HA	2.14	2.62	2.40	0.00
Leu11HA-Tyr2HE1	2.43	2.97	3.41	0.43
Leu11HA-HD2	1.62	2.87	4.42	1.55
Leu11HA-HN	1.98	2.41	2.90	0.49
Gln12HN-Leu11HA	1.52	1.86	2.20	0.34
Leu11HA-HB1	1.95	2.38	2.29	0.00
Leu11HA-HB2	1.82	2.23	2.93	0.70
Tyr2HE1-Leu11HD1	2.03	3.37	3.78	0.41
Leu11HA-HD1	1.46	2.68	2.80	0.12
Gln12HN-HA	2.12	2.59	2.90	0.31
Gln12HB1-HA	2.10	2.56	3.05	0.48
Gln12HB1-HN	2.30	2.82	2.45	0.00
Gln12HB2-HA	2.50	3.06	2.55	0.00
Gln12HA-HG1	1.86	2.28	2.76	0.48
Gln12HN-HG1	2.12	2.59	3.06	0.47

Table S16. Distance list of NOEs for compound **5,** considered for structure calculation and violation observed.

NOEs	Min. Distance	Max. Distance	Observed Distance	Violations
Arg1HA-HB1	1.99	2.43	3.00	0.57
Arg1HA-HB2	1.99	2.43	2.57	0.14
Arg1HA-HN	2.16	2.64	2.92	0.28
Tyr2HN-Arg1HA	2.09	2.55	2.39	0.00
Arg1HN-NAc	1.47	2.69	2.72	0.03
Tyr2HA-HB1	1.94	2.37	3.03	0.65
Tyr2HA-HB2	1.74	2.13	2.37	0.24
Tyr2HA-HN	2.15	2.62	2.92	0.30
Tyr2HA-Val3HN	1.68	2.06	2.24	0.18
Tyr2HA-Gln12HN	2.33	2.85	3.03	0.18
Tyr2HB1-HB2	1.74	2.12	1.75	0.00
Val3HN-HA	2.22	2.72	2.94	0.23
Glu4HN-Val3HA	2.04	2.50	2.49	0.00
Val3HA-HB	2.12	2.59	2.77	0.19
Val3HN-Tyr2HB2	2.39	2.92	2.85	0.00
Glu4HA-Val5HN	1.67	2.04	2.09	0.05
Val5HN-HA	2.03	2.48	2.87	0.39
Val5HA-Val6HN	1.65	2.01	2.18	0.17
Val5HA-HB	2.09	2.55	2.54	0.00
Val6HN-Val5HA	1.85	2.26	2.18	0.00
Val6HA-HN	2.16	2.64	2.86	0.22
Val6HA-HG1	1.50	2.72	2.99	0.27
Val6HA-HG2	1.53	2.75	2.94	0.19
Arg7NMe-Val6HG2	1.31	3.37	3.65	0.28
Arg7HA-HG1	2.20	2.69	2.76	0.08
Lys8HN-Arg7HA	2.07	2.53	3.02	0.49
Arg7HA-HB1	1.83	2.24	2.59	0.35
Arg7HA-HB2	2.33	2.84	2.97	0.12

Arg7HA-HG2	2.10	2.57	2.48	0.00
Val6HA-Arg7NMe	1.48	2.70	2.57	0.00
Lys8HN-Arg7NMe	1.49	2.71	2.91	0.20
Lys8HA-HB2	1.96	2.40	2.46	0.06
Lys8HA-HN	2.03	2.48	2.92	0.44
Lys9HN-Lys8HA	1.57	1.91	2.23	0.32
Lys8HB1-Lys9HN	2.17	2.66	2.50	0.00
Lys8HD1-HE1	1.89	2.31	2.49	0.18
Lys9HA-HB1	2.02	2.47	3.01	0.54
Lys9HA-HB2	1.57	1.92	2.36	0.44
Lys9HA-HN	2.22	2.71	2.91	0.20
Lys9HA-Ile10HN	1.68	2.05	2.17	0.12
Ile10HA-HB	1.95	2.39	2.40	0.02
Ile10HA-HN	2.20	2.69	2.94	0.24
Leu11HN-Ile10HD	1.63	2.89	2.86	0.00
Tyr2HA-Leu11HA	1.81	2.21	2.63	0.42
Leu11HA-HN	2.03	2.48	2.93	0.45
Gln12HN-Leu11HA	1.70	2.08	2.21	0.14
Leu11HA-HB1	2.05	2.51	2.96	0.45
Leu11HA-HB2	2.02	2.47	2.25	0.00
Leu11HB1-HB2	1.71	2.09	1.73	0.00
Leu11HD1-Tyr2HE1	1.94	3.26	3.16	0.00
Leu11HA-HD1	1.69	2.95	3.31	0.36
Leu11HA-HD2	1.85	3.15	4.23	1.08
Gln12HB1-HA	2.31	2.83	3.05	0.22
Gln12HN-HA	2.08	2.54	2.92	0.38
Gln12HA-HB2	2.07	2.53	2.50	0.00
Arg1HN-Gln12HN	2.51	3.07	3.15	0.08
Lys8HN-Val5HN	2.92	3.56	3.53	0.00
Arg1HA-HG1	2.12	2.59	2.53	0.00
Arg1HA-HG2	2.23	2.73	2.96	0.24
Tyr2HA-HD1	1.89	2.31	2.58	0.27
Tyr2HD1-HB2	1.82	2.22	2.33	0.11
Leu11HA-Tyr2HE1	2.47	3.02	3.38	0.36
Leu11HB2-Tyr2HD1	2.12	2.59	3.03	0.44
Leu11HB2-Tyr2HE1	1.96	2.40	2.24	0.00
Gln12HA-HB2	2.07	2.53	2.50	0.00
Gln12HA-HG1	1.96	2.40	2.58	0.18
Tyr2HD1-Lys9HB1	2.76	3.38	3.60	0.22
Tyr2HD1-Lys9HB2	2.37	2.89	3.05	0.16

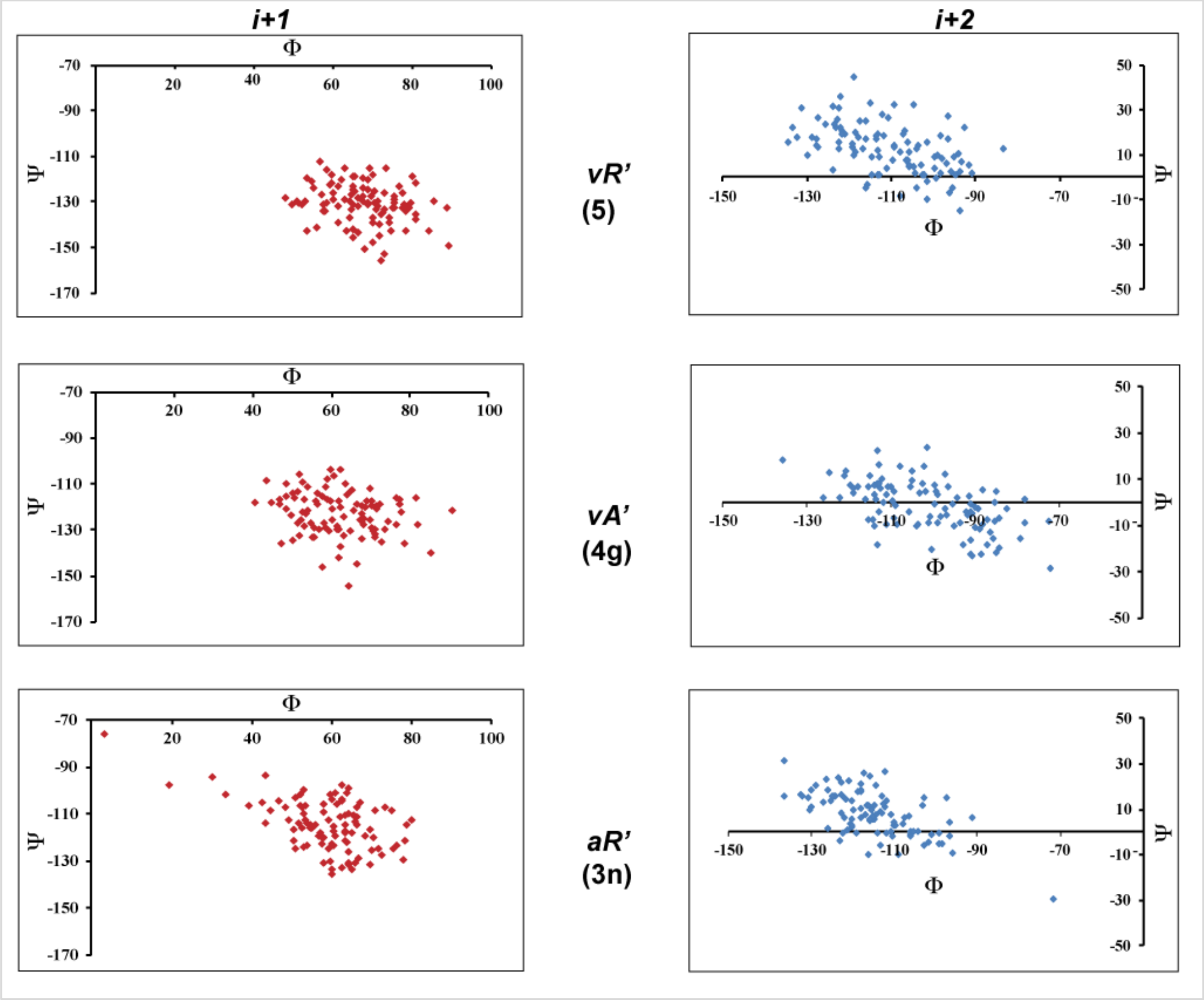

Figure S48. Ramachandran plot of *i+1* and *i+2* residues of 100 minimum energy conformations of compounds **5**, **4g** and **3n**.

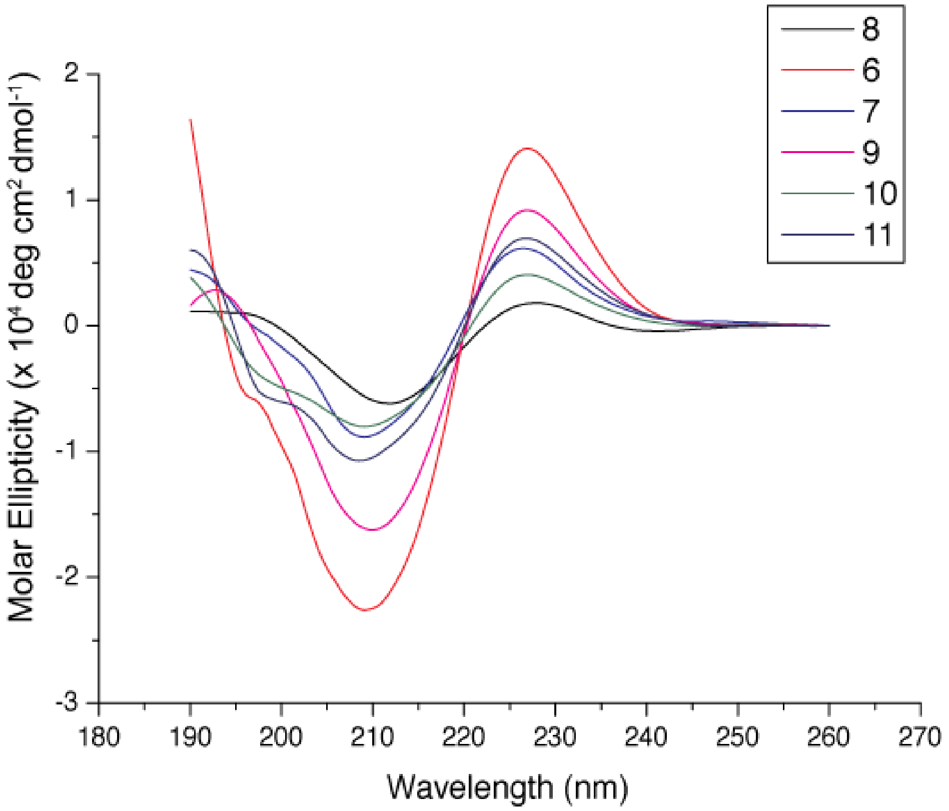

Figure S49. Overlayed Circular Dichroism spectra of **6-11.**

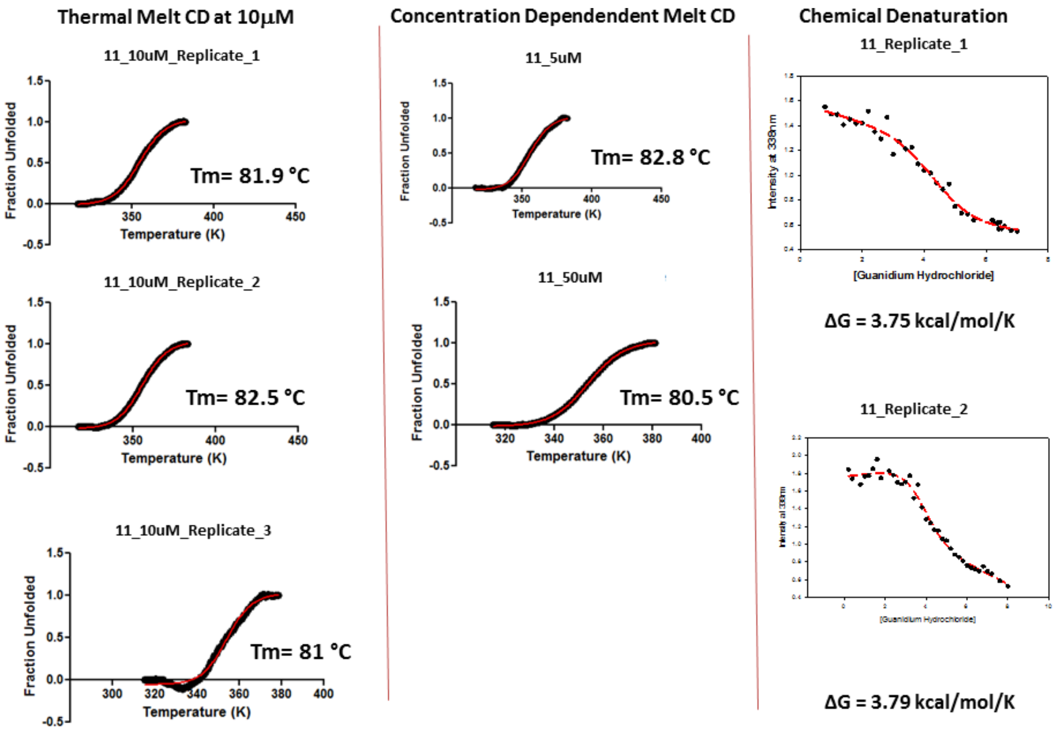

Figure S50. Thermal, concentration dependent denaturation CD and fluorescence monitored chemical denaturation of Compound **11.**

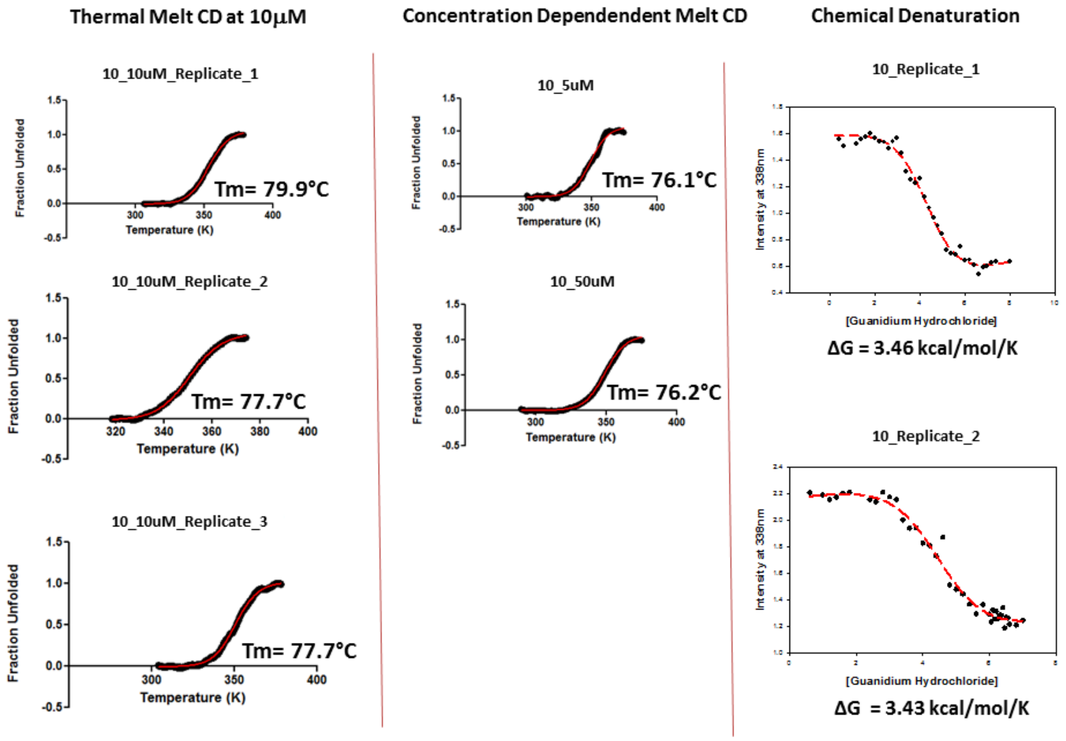

Figure S51. Thermal, concentration dependent denaturation CD and fluorescence monitored chemical denaturation of Compound **10.**

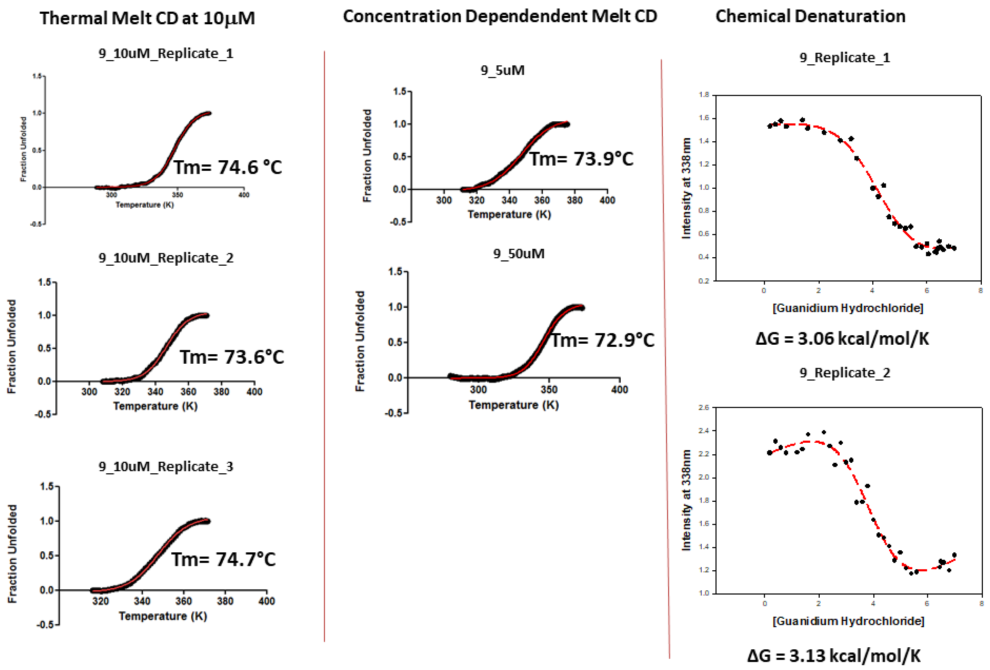

Figure S52. Thermal, concentration dependent denaturation CD and fluorescence monitored chemical denaturation of Compound **9.**

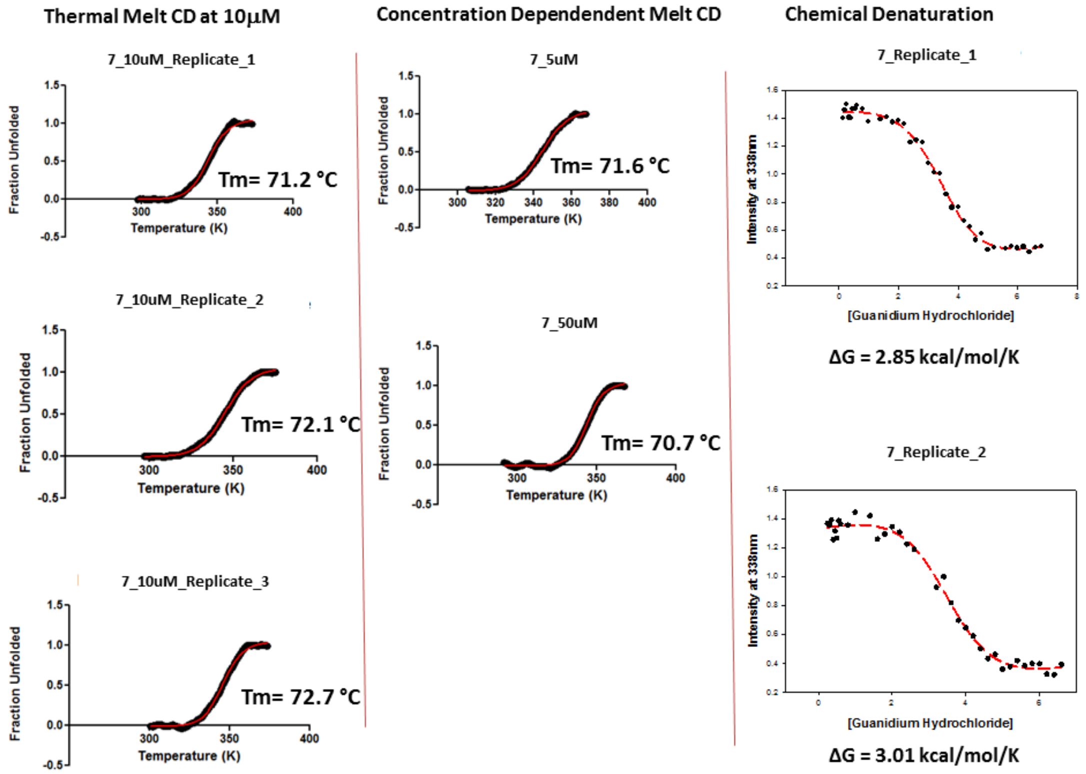

Figure S53. Thermal, concentration dependent denaturation CD and fluorescence monitored chemical denaturation of Compound **7.**

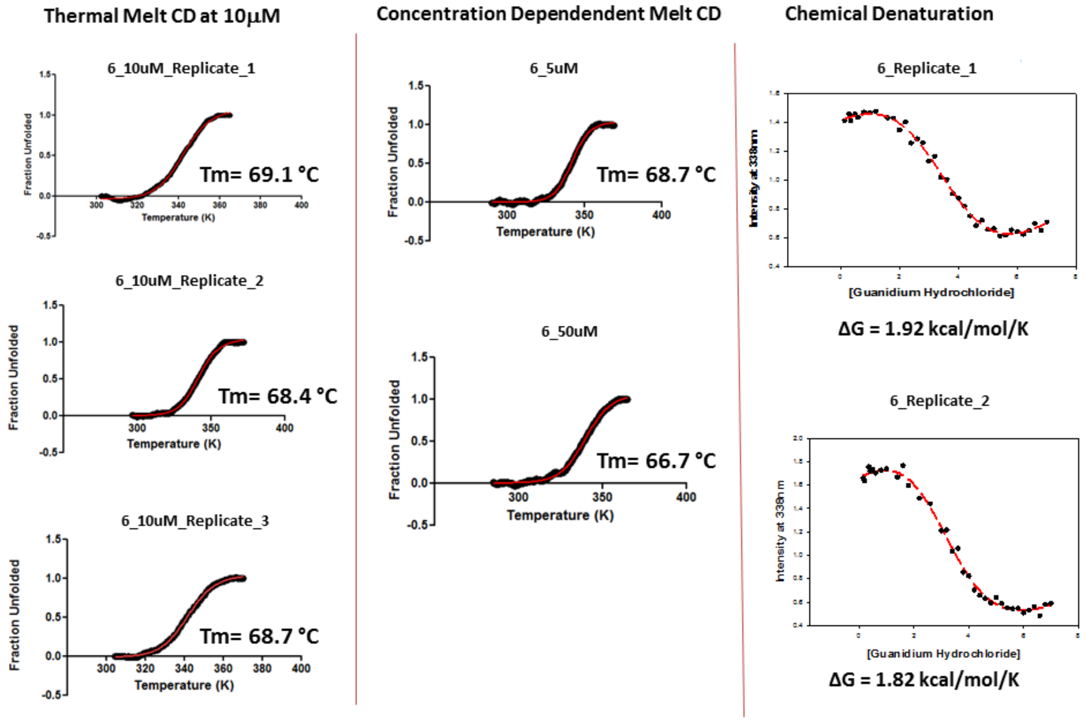

Figure S54. Thermal, concentration dependent denaturation CD and fluorescence monitored chemical denaturation of Compound **6.**

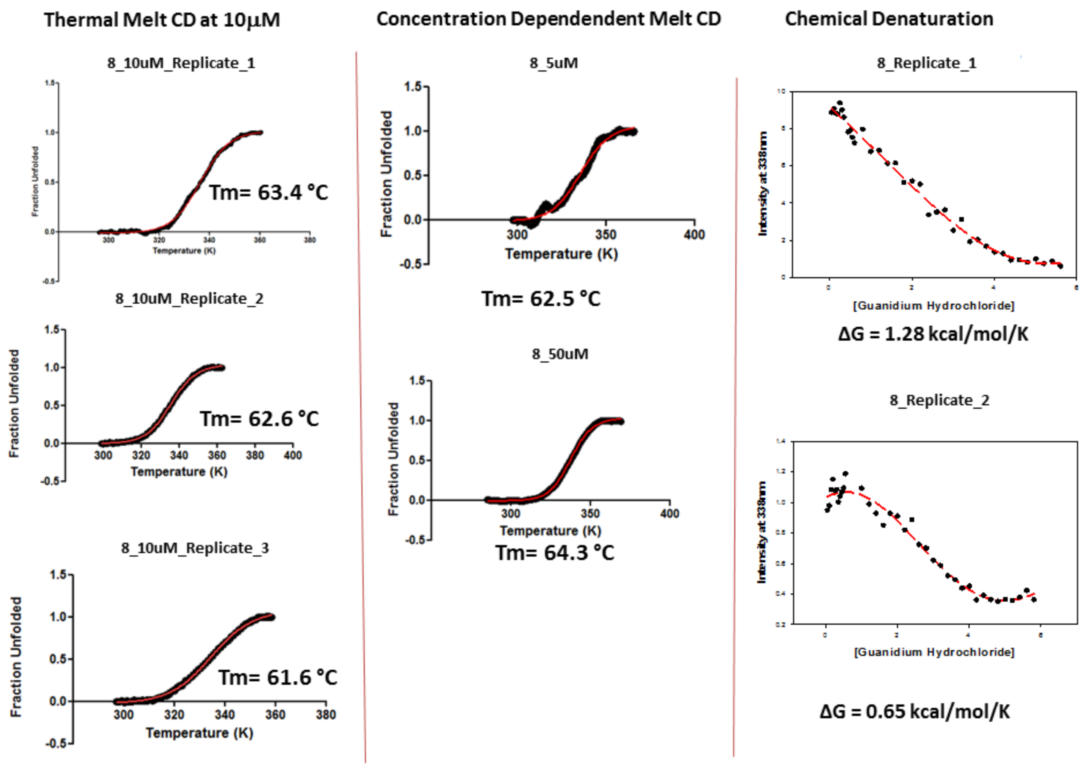

Figure S55. Thermal, concentration dependent denaturation CD and fluorescence monitored chemical denaturation of Compound **8**.

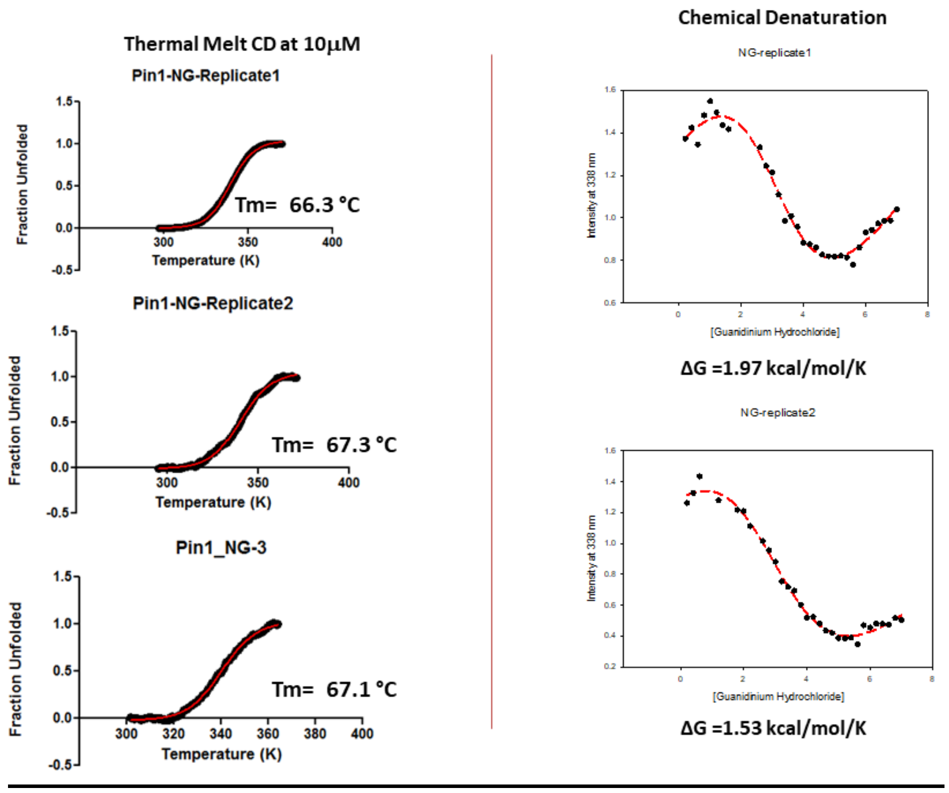

Figure S56. Thermal denaturation CD and fluorescence monitored chemical denaturation of Compound **NG**.

Figure S57. Characteristic NOEs of Pin1-WW analogs.

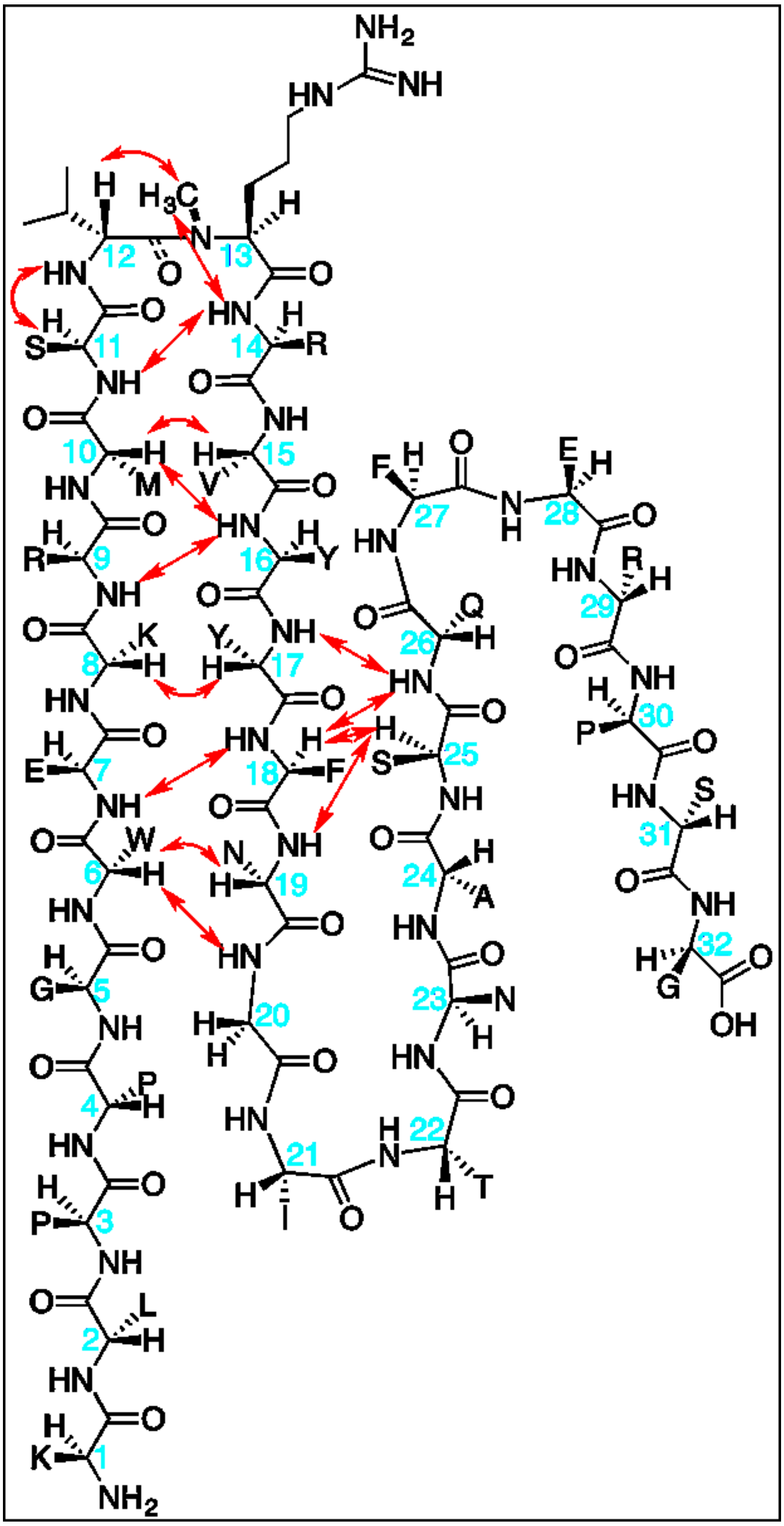

(a). Strand NOEs

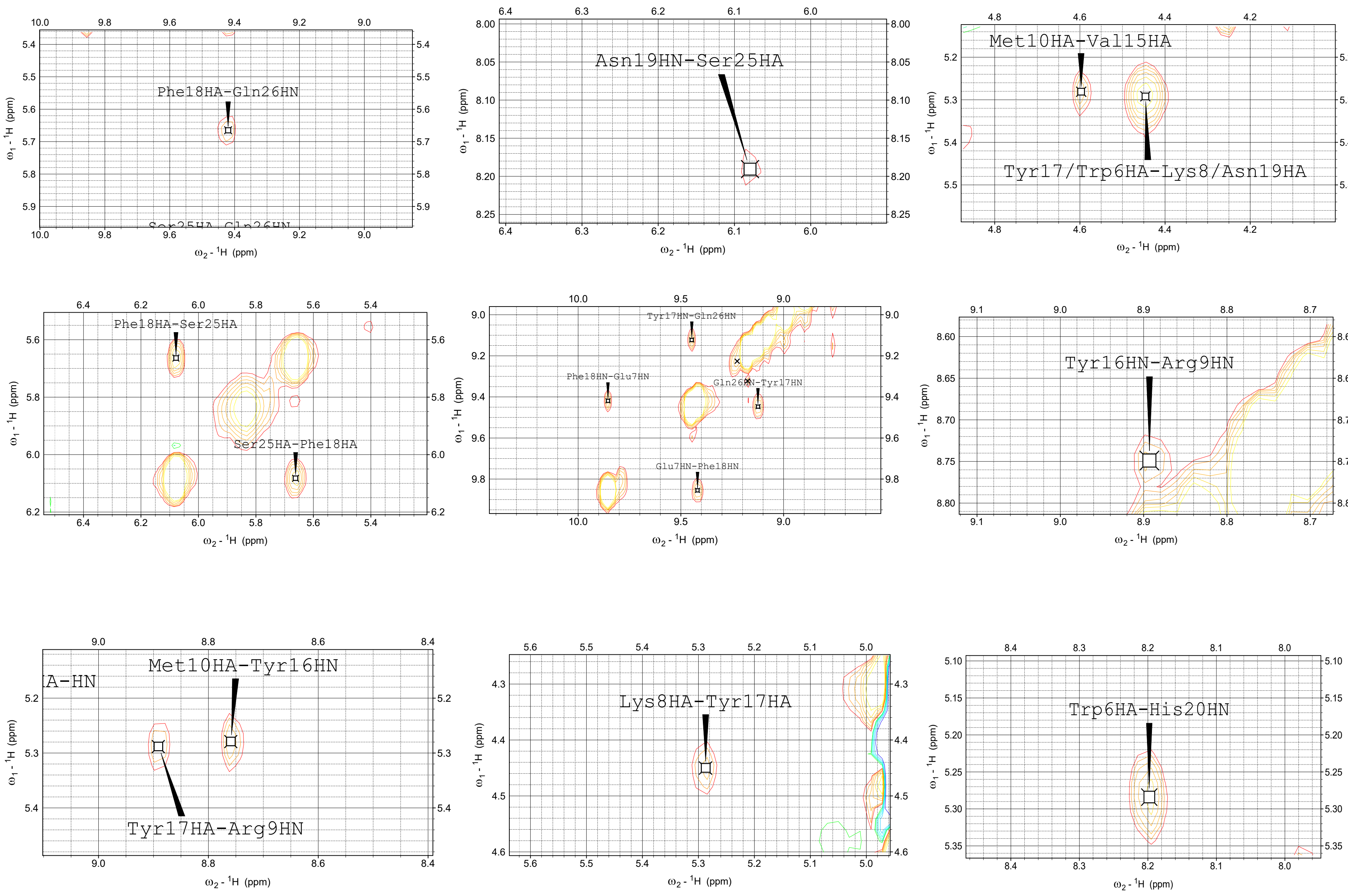

(b). Turn NOEs

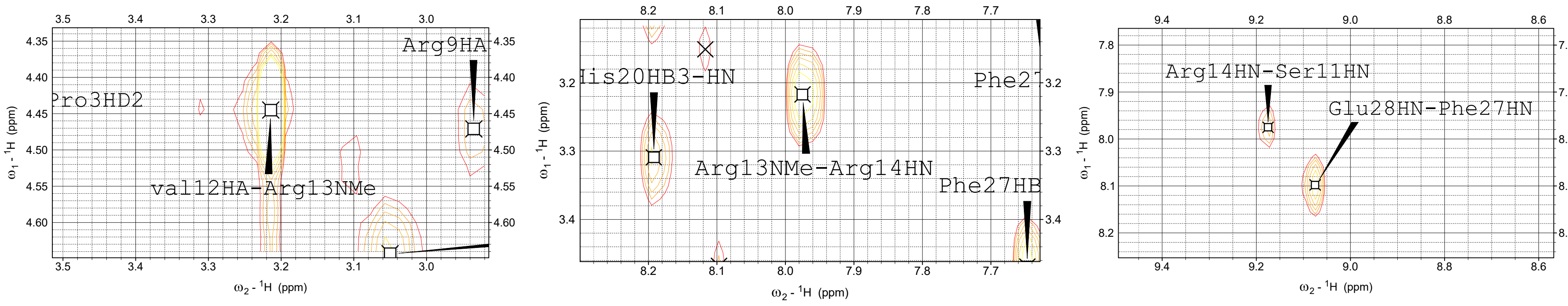

Figure S58. Representative characteristic NOEs observed in **11**.

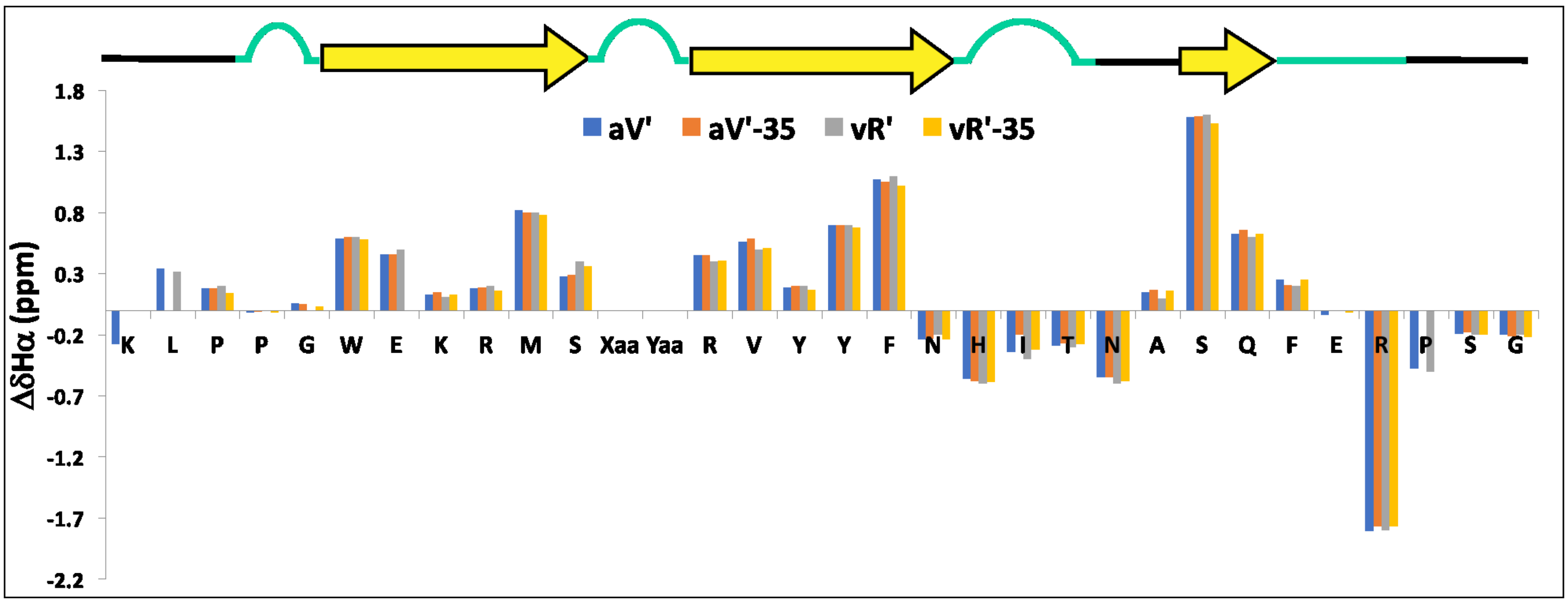

Figure S59. Secondary chemical shifts of Pin1 compounds **8 (aV')** and **11 (vR')** obtained at 288 K and 308 K.

Table S17. Amide temperature coefficients of Pin1 analogs **8** and **11**.

	**8** (aV')	**11** (vR')
**K**	-	-
**L**	-	-
**P**	-	-
**P**	-	-
**G**	5.5	5
**W**	0	0
**E**	3	-
**K**	5.5	5
**R**	3	2.5
**M**	5	5
**S**	5	4
**Xaa**	-	-
**Yaa**	-	-
**R**	3	2
**V**	6.5	7
**Y**	1.5	0.5
**Y**	3.5	2
**F**	1	0.5
**N**	3.5	2.5
**H**	2	0.5
**I**	2	4
**T**	0.5	1
**N**	2	1.5
**A**	1.5	1.5
**S**	5.5	5.5
**Q**	1.5	1
**F**	1	0
**E**	4	4
**R**	7	6
**P**	-	-
**S**	6.5	6
**G**	6	5.5

References.

1. J. Chatterjee, B. Laufer, H. Kessler, *Nat. Protoc.* 2012**,** **7**, 432.
2. G. N. Ramachandran, V. Sasisekharan, *Adv. Protein Chem.* 1968**,** **23**, 283.
3. S. Zhang, S. Prabpai, P. Kongsaeree, P. I. Arvidsson, *Chem. Commun.* 2006, 497.
4. R. Kaul, A. R. Angeles, M. Jager, E. T. Powers, J. W. Kelly, *J. Am. Chem. Soc.* 2001, **123**, 5206.
5. R. F. Greene, C. N. Pace, *J. Biol. Chem.* 1974, **249**, 5388.
6. F. A. Syud, J. F. Espinosa, S. H. Gellman, *J. Am. Chem. Soc.* 1999, **121**, 11577.
7. T. D. Goddard and D. G. Kneller, SPARKY 3, University of California, San Francisco.
8. R. R. Brooks, R. E. Bruccoleri, B. D. Olafson, D. J. States, S. Swaminathan, M. Karplus, *J. Comput. Chem*. 1983, **4**, 187.
9. C. Mas-Moruno, J. G. Beck, L. Doedens, A. O. Frank, L. Marinelli, S. Cosconati, E. Novellino, H. Kessler, *Angew. Chem. Int. Ed.* 2011, **50**, 9496.
10. V. F. Bystrov, V. T. Ivanov, S. L. Portnova, T. A. Balashova, Y. A. Ovchinnikov, *Tetrahedron* 1973, **29**, 873.
11. T. Cierpicki, J. Otlewski, *J. Biomol. NMR* 2001, **21**, 249.
